# Comparative histopathologic and viral immunohistochemical studies on CeMV infection among Western Mediterranean, Northeast-Central, and Southwestern Atlantic cetaceans

**DOI:** 10.1371/journal.pone.0213363

**Published:** 2019-03-20

**Authors:** Josué Díaz-Delgado, Kátia R. Groch, Eva Sierra, Simona Sacchini, Daniele Zucca, Óscar Quesada-Canales, Manuel Arbelo, Antonio Fernández, Elitieri Santos, Joana Ikeda, Rafael Carvalho, Alexandre F. Azevedo, Jose Lailson-Brito, Leonardo Flach, Rodrigo Ressio, Cristina T. Kanamura, Marcelo Sansone, Cíntia Favero, Brian F. Porter, Cinzia Centelleghe, Sandro Mazzariol, Ludovica Di Renzo, Gabriella Di Francesco, Giovanni Di Guardo, José Luiz Catão-Dias

**Affiliations:** 1 Laboratory of Wildlife Comparative Pathology, School of Veterinary Medicine and Animal Science, University of São Paulo, São Paulo, SP, Brazil; 2 Institute for Animal Health and Food Safety, School of Veterinary Medicine, University of Las Palmas of Gran Canaria, Arucas, Gran Canaria, Spain; 3 Laboratory of Aquatic Mammals and Bioindicators: Profa Izabel M. G. do N. Gurgel’(MAQUA), Faculty of Oceanography, Rio de Janeiro State University, Maracanã, Rio de Janeiro, RJ, Brazil; 4 Projeto Boto cinza, Mangaratiba, Rio de Janeiro, RJ, Brazil; 5 Adolfo Lutz Institute (IAL)–Pathology Center, Pacaembú, São Paulo, SP, Brazil; 6 Department of Veterinary Pathobiology, College of Veterinary Medicine & Biomedical Sciences, Texas A&M University, College Station, TX, USA; 7 Department of Comparative Biomedicine and Food Hygiene (BCA), University of Padova, Agripolis, Legnaro, Padova, Italy; 8 Istituto Zooprofilattico Sperimentale dell’Abruzzo e del Molise “G.Caporale”, Teramo, Italy; 9 Faculty of Veterinary Medicine, Località Piano d'Accio, University of Teramo, Teramo, Italy; Universidad de los Andes, COLOMBIA

## Abstract

Cetacean morbillivirus (CeMV) is a major natural cause of morbidity and mortality in cetaceans worldwide and results in epidemic and endemic fatalities. The pathogenesis of CeMV has not been fully elucidated, and questions remain regarding tissue tropism and the mechanisms of immunosuppression. We compared the histopathologic and viral immunohistochemical features in molecularly confirmed CeMV-infected Guiana dolphins (*Sotalia guianensis*) from the Southwestern Atlantic (Brazil) and striped dolphins (*Stenella coeruleoalba*) and bottlenose dolphins (*Tursiops truncatus*) from the Northeast-Central Atlantic (Canary Islands, Spain) and the Western Mediterranean Sea (Italy). Major emphasis was placed on the central nervous system (CNS), including neuroanatomical distribution of lesions, and the lymphoid system and lung were also examined. Eleven Guiana dolphins, 13 striped dolphins, and 3 bottlenose dolphins were selected by defined criteria. CeMV infections showed a remarkable neurotropism in striped dolphins and bottlenose dolphins, while this was a rare feature in CeMV-infected Guiana dolphins. Neuroanatomical distribution of lesions in dolphins stranded in the Canary Islands revealed a consistent involvement of the cerebrum, thalamus, and cerebellum, followed by caudal brainstem and spinal cord. In most cases, Guiana dolphins had more severe lung lesions. The lymphoid system was involved in all three species, with consistent lymphoid depletion. Multinucleate giant cells/syncytia and characteristic viral inclusion bodies were variably observed in these organs. Overall, there was widespread lymphohistiocytic, epithelial, and neuronal/neuroglial viral antigen immunolabeling with some individual, host species, and CeMV strain differences. Preexisting and opportunistic infections were common, particularly endoparasitism, followed by bacterial, fungal, and viral infections. These results contribute to understanding CeMV infections in susceptible cetacean hosts in relation to factors such as CeMV strains and geographic locations, thereby establishing the basis for future neuro- and immunopathological comparative investigations.

## Introduction

Cetacean morbillivirus (CeMV; genus *Morbillivirus*, family Paramyxoviridae) is the most significant natural cause of morbidity and mortality in cetaceans worldwide, having caused multiple outbreaks of lethal disease in odontocetes and mysticetes [1]. Interepizootic, generally endemic fatalities have also been recorded [2,3,4,5]. CeMV includes three well characterized strains: porpoise morbillivirus, dolphin morbillivirus (DMV), and pilot whale morbillivirus, all of which have been reported primarily in the Northern Hemisphere. Three new strains have been reported, one of them detected in Brazil and considered the first description in South America, namely Guiana dolphin (*Sotalia guianensis*) (GD)-CeMV [6]. CeMV may cause severe respiratory, lymphoid, and neurologic disease in susceptible species, leading to strandings and death [1]. Studies suggest that some species are more susceptible to CeMV infection, including striped dolphins (*Stenella coeruleoalba*) and bottlenose dolphins (*Tursiops truncatus*) [1,7]. The high susceptibility of Guiana dolphins to GD-CeMV was recently demonstrated in the first unusual mortality event (UME) in South American cetaceans, in which approximately 250 Guiana dolphins died [8].

As in the case of other highly contagious and infectious morbilliviruses, such as measles virus (MeV) and canine distemper virus (CDV), CeMV is lymphotrophic and epitheliotropic with common central nervous system (CNS) involvement [1]. Pathogenetic similarities exist among morbilliviruses [9,10,11], and much knowledge of CeMV has been gained by extrapolating from studies of MeV and CDV. Knowledge gathered since its first description in the late 1980’s [12] has led to distinction of four major disease presentations: acute (AS), subacute (SS), chronic systemic (CS), and chronic localized encephalitis “brain only form of DMV infection” (BOFDI) [1,3,5,13,14,15,16,17]. These presentations are based on distribution of histologic lesions, antigen detection, and molecular analysis [1], yet pathologic findings often overlap, and suboptimal sample preservation, limited tissue sampling, and restricted laboratory analyses inherent to wildlife postmortem investigations hinder this classification. Many questions about the pathogenesis of CeMV infection remain unanswered, particularly those concerning pulmonary disease, CNS disease, and the mechanism of viral-induced immunosuppression.

Since ethical issues preclude experimental infections, detailed investigations on naturally occurring CeMV cases are warranted. For a better understanding of CeMV infection in highly susceptible delphinids, we compared the histopathologic and immunohistochemical features of CeMV-infected Guiana dolphins from Brazil and striped dolphins and bottlenose dolphins from the Canary Islands (Spain) and Italy.

## Materials and methods

### Data and sample collection

The research institutions participating in the study were the Laboratory of Wildlife Comparative Pathology–LAPCOM (São Paulo, Brazil), the Laboratory of Aquatic Mammals and Bioindicators “Profa. Izabel M. G. do N. Gurgel”—MAQUA (Rio de Janeiro, Brazil), the Institute for Animal Health and Food Safety–IUSA (Canary Islands, Spain), the Department of Comparative Biomedicine and Food Science of the Faculty of Veterinary Medicine of the University of Padua (Legnaro, Italy), and the Laboratories of Histopathology and Immunohistochemistry of Istituto Zooprofilattico Sperimentale dell’Abruzzo e Molise “G. Caporale” as well as of the Faculty of Veterinary Medicine of the University of Teramo (Teramo, Italy). The marine mammal databases and tissue banks of these institutions were queried based upon the following criteria: ‘*Sotalia guianensis*’, ‘*Stenella coeruleoalba*’, ‘*Tursiops truncatus*’, ‘CeMV reverse transcription polymerase chain reaction (RT-PCR)-positive’ ‘*Toxoplasma gondii* PCR-negative.’ Complete standard necropsies were performed, and only individuals in a ‘fresh’ (code 2) post mortem preservation status, or in a ‘moderate post mortem autolysis’ (code 3) condition [18] were included. Another inclusion criterion was the presence of a sufficient amount of formalin-fixed, paraffin-embedded (FFPE) and frozen tissues for immunohistochemical and cytokine gene expression investigations (*manuscripts published elsewhere*) on target organs (brain, lymph nodes, spleen, lung). Epidemiologic and biologic data, necropsy reports, photographic material, and ancillary diagnostic techniques were retrieved and further analyzed. Required permissions for the management of tissues from cetaceans found stranded along the coasts of Brazil, the Canarian archipelago, and Italy were issued by the respective official authorities. No experiments were performed on live animals. A few specimens in this study were included in previously published studies [3,8,19,20,21]. The materials used in this study are deposited in the different tissue banks of the collaborative research institutions.

### Systematic histopathologic analyses

For histopathologic analysis, tissues were sectioned at 5 μm thickness and stained with hematoxylin-eosin (H&E). Tissue sections from the CNS, lung, spleen, and lymph nodes (mediastinal, pulmonary, prescapular, mesenteric) were evaluated systematically through designed templates (S1, S2 and S3 Tables), and a large variety of microscopic findings were recorded, including subjective evaluations of lesion severity and extension as minimal (-/+), mild (+), moderate (++), and severe (+++). Additional histochemical techniques, including periodic-acid Schiff (PAS, for basement membranes and fungi), Grocott (for fungi), and Luxol fast blue (LFB, for myelin) were carried out to better characterize microscopic changes in selected tissue sections. Systematic morphologic diagnoses were recorded for each animal and related to the cause of stranding and/or death (S4 Table).

### Neuroanatomical distribution of CeMV-associated microscopic findings

An analysis of neuroanatomical distribution of microscopic lesions was conducted on the brains of striped dolphins (n = 7) and bottlenose dolphin (n = 1) from the Canary Islands due to whole brain availability for broad and detailed sampling. Neuroanatomical areas consistently sampled in these animals included the frontal and temporo-parietal cerebrum, diencephalon including the thalamus, pons, medulla oblongata, cerebellum, and spinal cord (S1 Fig). One striped dolphin (case 18) also had the mesencephalon represented.

### Immunohistochemical analysis

Immunohistochemical (IHC) analyses employed the following primary antibodies (Abs): a monoclonal IgG2B (kappa light chain) Ab against the nucleoprotein (N) antigen of CDV (1:100 dilution; VMRD Inc, Pullman, WA, USA) that was used on FFPE brain, lymphoid tissue, and lung [8], a monoclonal Ab against an anti-cytokeratin cocktail (AE1/AE3; 1:2,000 dilution; Biocare Medical, CA, USA) that was used on selected lung sections, a polyclonal anti-S100 (1:100 dilution; Dako, Glostrup, Denmark) Ab, and a polyclonal anti-glial fibrillary acidic protein (GFAP) Ab (1:150 dilution; Eurodiagnostics, Appeldoorn, The Netherlands) for astrocytes. Dual IHC was performed in case 26 by means of the Envision Double Staining System protocol (K-1395, Dako), following manufacturer’s instructions, and included the anti-CDV Ab, anti-S100 Ab, and anti-GFAP Ab, in order to better define the origin of CeMV-positive neuroglia. Positive control tissue consisted of lung from a Guiana dolphin infected with GD-CeMV [8]. As a negative control, the primary antibody was eliminated and replaced by nonimmune homologous serum.

## Results

We analyzed 27 CeMV-positive dolphins, including 11 Guiana dolphins, 13 striped dolphins, and 3 bottlenose dolphins. Guiana dolphins were infected by GD-CeMV [6,8], while striped dolphins and bottlenose dolphins were infected by DMV [3,19,20,21]. Epidemiologic and biologic data, as well as CeMV-RT-PCR-positive tissues, are recorded in Table 1. Animals were of different ages: calves (n = 2), juveniles (n = 11), and adults (n = 14). Only one animal (case 14) had evidence of entanglement. Four animals were visually confirmed to live-strand, while 23 were found stranded dead. A summary of main lesions, morbilliviral antigen distribution, PCR-positive tissues, and suspected infection chronicity by geographic area and species are recorded in Table 2. Detailed pathologic findings with most probable cause(s) of stranding and/or death are recorded in S4 Table.

**Table 1:** Epidemiologic and biologic data on Guiana dolphins (*Sotalia guianensis*), striped dolphins (*Stenella coeruleoalba*), and bottlenose dolphins (*Tursiops truncatus*). All animals were cetacean morbillivirus (CeMV) positive by conventional and/or real time RT-PCR.

No	Species	Stranding	Country	BL (cm)	Age	Sex	NS	DC	SC
1	*S*. *guianensis*^a^	09-Nov-2017	BR	177	Ad	Fe	Po	2	D
2	*S*. *guianensis*^a^	14-Nov-2017	BR	94	Ca	Ma	Mo	2	D
3	*S*. *guianensis*	17-Dec-2017	BR	164	Ju	Ma	Mo	3	D
4	*S*. *guianensis*	17-Dec-2017	BR	93	Ca	Fe	Go	3	D
5	*S*. *guianensis*	23-Dec-2017	BR	149	Ju	Ma	Mo	2	D
6	*S*. *guianensis*	25-Dec-2017	BR	125	Ju	Ma	Po	3	D
7	*S*. *guianensis*	26-Dec-2017	BR	188	Ad	Fe	ND	3	D
8	*S*. *guianensis*	27-Dec-2017	BR	176	Ad	Ma	Mo	3	D
9	*S*. *guianensis*	27-Dec-2017	BR	183	Ad	Ma	Mo	3	D
10	*S*. *guianensis*	27-Dec-2017	BR	186	Ad	Ma	Po	3	D
11	*S*. *guianensis*	15-Jan-2018	BR	130	Ju	Fe	Po	2	D
12	*S*. *coeruleoalba*^*b*^	13-Nov-2002	SP	224	Ad	Ma	Go	2	A
13	*T*. *truncatus*^*c*^	18-Jul-2005	SP	250	Ju	Fe	Mo	2	A
14	*S*. *coeruleoalba*	16-Aug-2005	SP	168	Ju	Fe	Go	2	A
15	*S*. *coeruleoalba*^*b*^	16-Apr-2007	SP	195	Ju	Ma	Po	2	D
16	*S*. *coeruleoalba*^*b*^	02-May-2008	SP	194	Ju	Fe	Po	3	D
17	*S*. *coeruleoalba*^*b*^	22-Jan-2009	SP	212	Ad	Fe	Go	2	D
18	*S*. *coeruleoalba*^*b*^	10-Feb-2011	SP	215	Ad	Fe	Go	2	D
19	*S*. *coeruleoalba*	28-Apr-2012	SP	203	Ju	Ma	Mo	2	D
20	*S*. *coeruleoalba*	04-Jul-2011	IT	205	Ad	Ma	Mo	3	D
21	*S*. *coeruleoalba*	20-Oct-2013	IT	NR	Ad	Ma	ND	3	D
22	*S*. *coeruleoalba*	02-Feb-2013	IT	NR	Ad	Fe	ND	3	D
23	*T*. *truncatus*	20-Mar-2013	IT	203	Ju	Ma	ND	2	D
24	*S*. *coeruleoalba*	05-Feb-2013	IT	202	Ad	Ma	ND	2	D
25	*T*. *truncatus*^*d*^	30-Jun-2011	IT	297	Ad	Ma	Mo	2	A
26	*S*. *coeruleoalba*	12-Oct-2017	IT	200	Ad	Fe	Mo	2	D
27	*S*. *coeruleoalba*	10-Nov-2017	IT	188	Ju	Fe	Mo	3	D

NR, not recorded; Ca, calf; Ju, juvenile; Ad, adult; Fe, female; Ma, male; NS, nutritional status; Po, poor; Mo, moderate; G, good; DC, decomposition code (2, fresh; 3, moderate autolysis); SC, stranding condition (A: alive; D: dead). Partial results for these animals have been published (^a^, [8]; ^b^, [20]; ^c^, [21], ^d^, [3]).

**Table 2:** Summary of main lesions, morbilliviral antigen distribution and PCR-positive tissues (lung, lymph nodes, spleen, central nervous system), and suspected infection chronicity by geographic area and species.

No	Species	Main histopathologic findings	CeMV antigen distribution ^a^	CeMV PCR/RT-PCR positive tissue(s)	Chronicity
1	GD (BR)	Generalized lymphoid depletion. Bronchointerstitial pneumonia (overlapped with *Halocercus brasiliensis* pneumonia).	Lung, spleen, mesenteric LN, cerebrum, cerebellum, spinal cord	Lung, spleen, cerebrum^†^	AS
2	GD (BR)		Lung, trachea, spleen, mediastinal LN, cerebrum	Lung^†^	AS
3	GD (BR)		Lung, mediastinal LN, spleen	Lung^†^	SS
4	GD (BR)		Lung	Spleen, cerebrum ^†^	AS
5	GD (BR)		Lung, spleen	Lung, cerebrum ^†^	AS
6	GD (BR)		Lung	Lung, cerebrum ^†^	AS
7	GD (BR)		Lung	Lung, cerebrum ^†^	AS
8	GD (BR)		Lung, mediastinal LN	Lung^†^	AS
9	GD (BR)		Lung, mediastinal LN, cerebrum	Lung, cerebrum ^†^	AS
10	GD (BR)		Lung	Lung^†^	AS
11	GD (BR)		Lung, mediastinal LN, cerebrum, cerebellum	Lung, pulmonary LN, cerebrum^†^	SS
12	SD (CI)	Lymphoplasmacytic meningoencephalomyelitis. Multicentric lymphoid depletion with concomitant regenerative phenomena (hyperplasia). Bronchointerstitial pneumonia.	Lung, cerebrum, brainstem, spinal cord	Lung, cerebrum^‡^ [20,22]	CS
13	BD (CI)		Lung, spleen, LNs, cerebrum, cerebellum, brainstem, spinal cord	Lung, spleen, cerebrum^§^ [21]	SS
14	SD (CI)		Cerebrum	Lung, cerebrum^‡^ [20,22]	AS
15	SD (CI)		Cerebrum, cerebellum, brainstem, spinal cord	Cerebrum ^‡^ [20,22]	AS
16	SD (CI)		Cerebrum	Cerebrum ^‡^ [20,22]	BOFDI
17	SD (CI)		Cerebrum	Cerebrum ^‡^ [20,22]	BOFDI
18	SD (CI)		Lung, cerebrum, cerebellum, brainstem, spinal cord	Lung, cerebrum ^‡^ [20,22]	CS
19	SD (CI)		Cerebrum, cerebellum, brainstem	Lung, cerebrum ^‡^ [20,22]	AS
20	SD (IT)	Lymphoplasmacytic meningoencephalitis. Multicentric lymphoid depletion with concomitant regenerative phenomena (hyperplasia). Bronchointerstitial pneumonia.	Cerebrum	Cerebrum ^¶^ [23]	BOFDI
21	SD (IT)		Neg	Spleen ^¶^ [23]	AS
22	SD (IT)		Neg	Lung, cerebrum ^±^ [24]	CS
23	BD (IT)		Cerebrum	Lung, cerebrum ^±^ [24]	AS
24	SD (IT)		Neg	Lung ^¶^ [23]	CS
25	BD (IT)		Lung, cerebrum	Cerebrum * [19]	SS
26	SD (IT)		Lung, cerebrum, spinal cord	Lung, mediastinal LN, cerebrum, spinal cord ^‡^ [20,22]	AS
27	SD (IT)		Neg	Lung, mediastinal LN, cerebrum, spinal cord ^‡^ [20,22]	SS

GD, Guiana dolphin (*Sotalia guianensis*); SD, striped dolphin (*Stenella coeruleoalba*); BD, bottlenose dolphin (*Tursiops truncatus*); BR, Brazil; CI, Canary Islands (Spain); IT, Italy; LN, lymph node; NE, not evaluated; Neg, negative; AS, acute systemic; SS, subacute systemic, CS, chronic systemic; BOFDI, brain only form of DMV infection.

^a^ All Guiana dolphins had detectable morbilliviral antigen in additional tissues, accounting for systemic presentation (a detailed and complete list including cell types is published elsewhere).

† RT-PCR

‡Conventional and/or real time RT-PCR.

§ RT-PCR.

¶ RT-PCR.

± Real Time RT-PCR.

* Nested RT-PCR.

### Central nervous system

**Fig 1:**
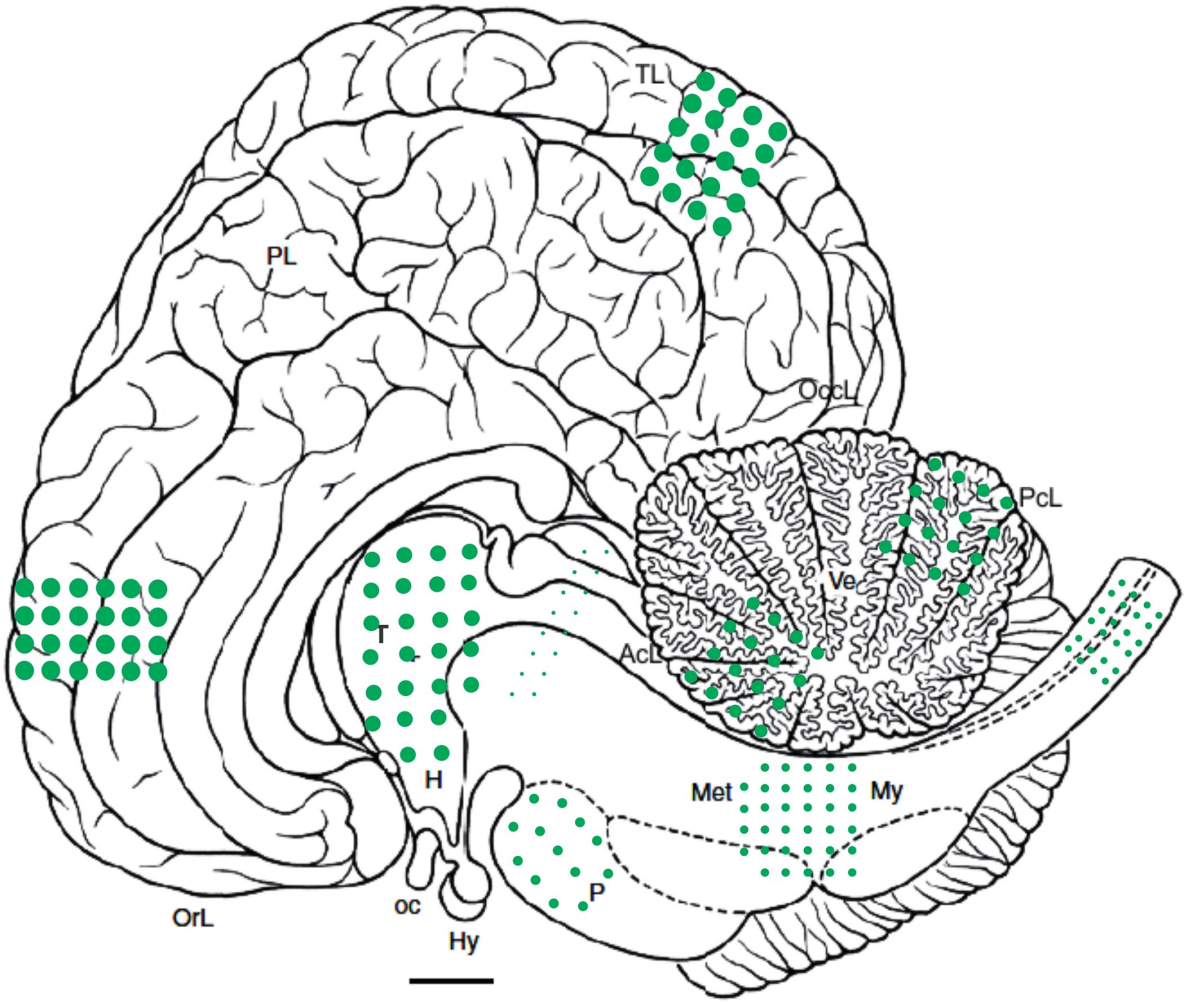
Neuroanatomical distribution and severity of dolphin morbillivirus-associated lesions in striped dolphins (*Stenella coeruleoalba*) and bottlenose dolphin (*Tursiops truncatus*) from the Canary Islands (Spain). The size and density of dots indicates the severity and extent of the lesions. Bar: 1 cm. AcL, anterior cerebellar lobe; H, hypothalamus; Hy, hypophysis; Met, metencephalon; My, myelencephalon; oc, optic chiasm; OccL, occipital lobe; OrL, orbital lobe; PcL, posterior cerebellar lobe T; PL, paralimbic lobe; thalamus; TL, temporal lobe; Ve, vermis. Brain diagram adapted from Oelschlager, H. & Oelschlager, J. (2009).

**Fig 2:**
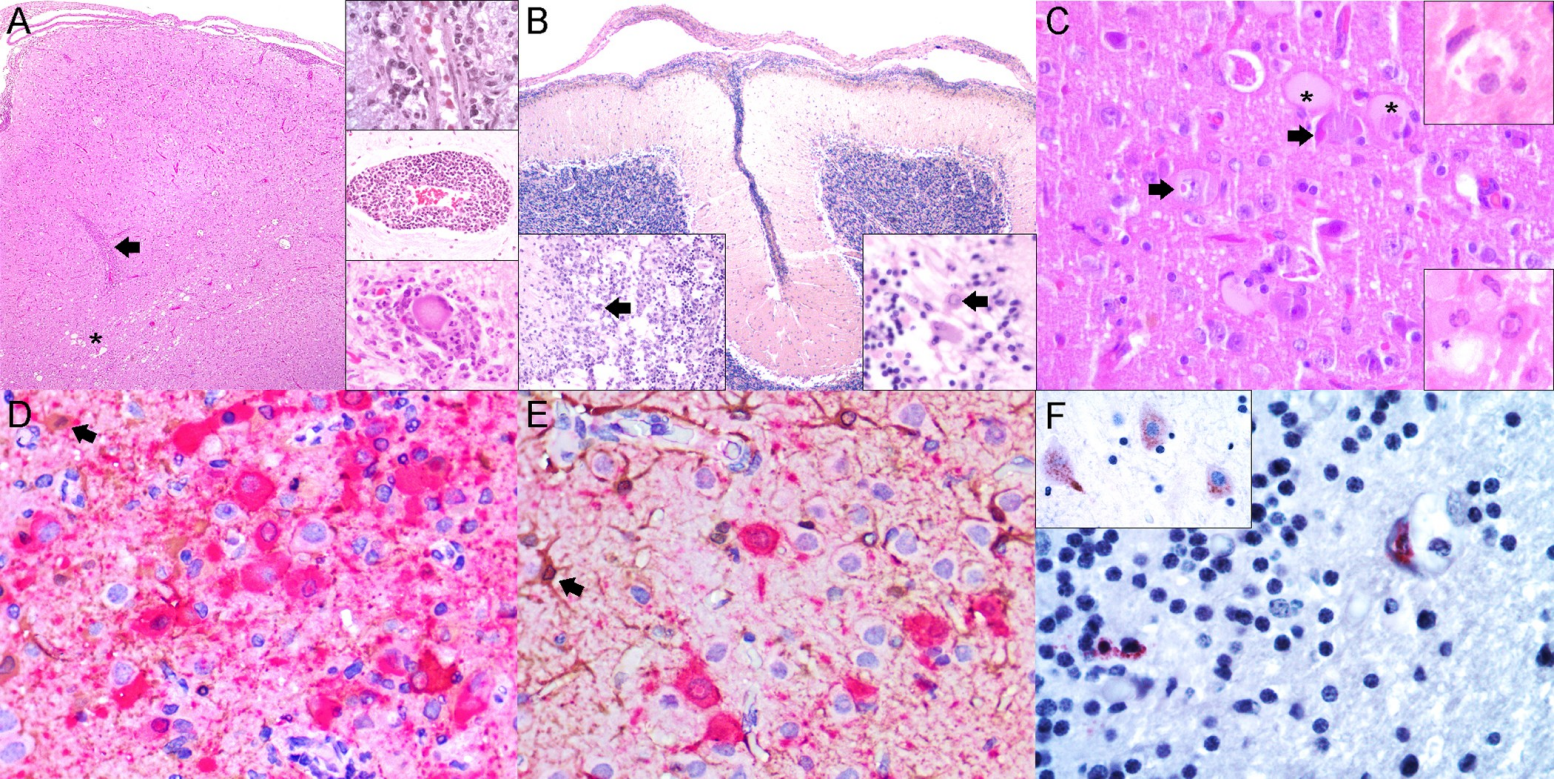
Cetacean-morbillivirus (CeMV)-associated lesions in central nervous system of striped dolphins (*Stenella coeruleoalba*), bottlenose dolphins (*Tursiops truncatus*), and Guiana dolphins (*Sotalia guianensis*). (A) Cerebral meningoencephalitis (case 14). The leptomeninges are inflamed and there is increased cellularity in the underlying cortex and subcortical white matter. A large perivascular cuff (arrow) and a few spongy foci (asterisk) are noted. 20x. H&E. Right upper inset: small lymphocytic and plasmacytic perivascular cuff in the cerebral cortex (case 14). 400x. H&E. Right middle inset: thick perivascular cuff at the gray/white matter interface (case 18). 200x. H&E. Right lower inset: degenerating neuron cell body with microglial neuronophagic nodule (case 19). 400x. H&E. (B) Cerebellar meningoencephalitis (case 13). Diffusely, the pia mater, arachnoid, and dura matter are infiltrated by mononuclear inflammatory cells. 40x. H&E. Left inset: mononuclear inflammation at molecular, Purkinje cell, and inner granular cell layer interface with edema, rarefaction, and focal multinucleate giant cell/syncytial cell (MGCS; arrow) (case 19). 200x. H&E. Right inset: Less severely inflamed cerebellar interface area with three degenerating Purkinje cells, one of which displays a viral intranuclear inclusion body (arrow) (case 19). 400x. H&E. (C) Cerebral cortex (case 26). Degenerating swollen neurons and necrotic neurons with intranuclear and intracytoplasmic viral inclusion bodies. 400x. H&E. Right upper inset: Degenerating swollen astrocyte with intracytoplasmic inclusions. 400x. H&E. Right lower inset: Degenerating neuron with intranuclear inclusion body and adjacent necrotic neuron. 400x. H&E. (D) Cerebral cortex (case 26). Marked positive labeling for CeMV-antigen (red) in degenerating neurons and fewer astrocytes (GFAP-brown) (arrow). There is loss of astrocytic processes and cell bodies in this focus. 400x. Double IHC for CeMV and GFAP. (E) Cerebral cortex (case 26). Moderate labeling for CeMV-antigen in neurons and less so in astrocytes (arrow). There is relative preservation of astrocytes in this focus compared to figure (D) but those present have plump nuclei, cytoplasm, and processes. Note astrocytic processes reaching the perivascular space show occasional positivity for CeMV as well. 400x. Double IHC for CeMV and GFAP. (F) Cerebellum (case 1). There is positive labeling for CeMV-antigen in endothelium focally and adjacent neuroglia. 400x. IHC for CeMV. Inset: CeMV positive labeling in neuronal soma and axon hillock of frontal cerebral cortex (case 9). 400x. IHC for CeMV.

Consistent CNS involvement was detected in striped dolphins and bottlenose dolphins stranded in the Canary Islands and Italy. Only two Guiana dolphins (cases 9 and 10) had lesions suggestive of CeMV infection, namely focal lymphocytic leptomeningitis. DMV-associated lesions in striped dolphins and bottlenose dolphins were of varying severity and extent. There was a widespread neuroanatomical distribution of lesions (Fig 1) by decreasing frequency and severity: cerebral cortex (Fig 2A), thalamus, cerebellum (Fig 2B), caudal brainstem, and spinal cord. Randomly sampled spinal nerves and ganglia were rarely affected. The meninges were consistently involved in all locations. The mesencephalon was underrepresented in the sample set (n = 1; case 18).

The main microscopic findings in the CNS are summarized in S5 Table. Inflammatory lesions, namely leptomeningeal infiltrates and perivascular cuffing, were more pronounced at the gray/white matter interface and were largely composed of lymphocytes with fewer plasma cells and macrophages. Degenerative changes occurred typically in gray matter and along the gray/white matter interface and involved neurons and neuroglia, with satellitosis, nuclear margination, neuronal necrosis (Fig 2C), neuronophagic nodules (Inset in Fig 2A), neuronal chromatolysis, axonal swelling, astrocytosis/astrogliosis, oligodendrogliosis, spongiosus/myelinic edema, and microglial hyperplasia and hypertrophy. Characteristic intranuclear and/or intracytoplasmic inclusion bodies (INCIBs) were readily evident in some animals within neurons (cortical, subcortical, and deep nuclei, Purkinje cells, and rare spinal neurons), astrocytes, and oligodendrocytes. Multinucleate giant cells/syncytia (MGCS) were seen in some animals. Reactive changes were associated with the aforementioned degenerative phenomena or spanned the underlying and occasionally distant white matter. Mild spongiosis was a common feature at the gray/white matter interface and in the white matter; however, LFB failed to demonstrate significant demyelination in these areas.

Some variations among AS cases and between AS and CS cases were readily evident. Cases 14 (AS) and 26 (AS) had severe neurodegenerative changes in the frontal and temporo-parietal cerebral cortical gray matter, while inflammatory and reactive changes predominated in the white matter. Case 19 (AS) had mild neurodegenerative and reactive changes in the cortical gray matter and thalamus, with absent to minimal inflammation; however, there was severe neurodegenerative and reactive changes with extensive necrosis, neuronal/neuroglial loss, and scattered foci of demyelination in metencephalic and myelencephalic sections. In these areas, reactive changes and inflammation differed from all other cases, with marked and extensive microgliosis, glial nodules, vasculitis, syncytia, edema, and frequent INCIBs. Case 18 (CS) had nearly identical severe inflammatory and reactive changes throughout the cerebrum, thalamus, mesencephalon, caudal brainstem, spinal cord, and cerebellum, mainly in the gray matter and at the gray/white matter interface; however, neurodegenerative changes were mild. All dolphins stranded in Italy had similar findings, but further comparisons were limited by the fact that only the cerebrum was consistently represented in the sample set.

Two bottlenose dolphins (cases 13 and 25) had CNS lesions diverging from but overlapping with classical CeMV-associated CNS pathology. Case 13 had pleocellular lymphohistiocytic to granulomatous inflammation widely affecting the meninges and neuroparenchyma of the cerebrum, thalamus, medulla oblongata, pons, cerebellum, neurohypophysis, spinal cord, the ventricular system with their respective choroid plexuses, and multiple spinal nerves and ganglia. Vasculitis and chronic endarteritis with marked intimal hyperplasia were common in metencephalon and myelencephalon. This was a suspected case of *Brucella* and CeMV co-infection based on cellular inflammatory components and neuroanatomical distribution of lesions. Case 25 had concurrent fibrinosuppurative meningoencephalitis and a positive culture of coagulase-positive *Staphylococcus aureus* [3].

Dolphins stranded in the Canary Islands and Italy showed morbilliviral antigen immunolabeling in neurons (perykaria, dendrites, axons, and axonal hillocks) (Fig 2D and 2E), including Purkinje cells, as well as in neuroglia, mostly in astrocytes and oligodendrocytes, throughout most neuroanatomic locations. Double IHC labeling with the anti-CDV, anti-GFAP, and anti-S100 Abs proved the neuroglial origin (astrocytes and oligodendrocytes) of morbilliviral antigen-bearing cells. The abundance and intensity of the immunostaining reaction mostly paralleled the severity and extent of the neurodegenerative, inflammatory, and reactive changes in the cerebrum, thalamus, cerebellum, caudal brainstem, and spinal cord. Animals with more pronounced neurodegenerative changes (AS and CS cases) had a greater amount and more widespread distribution of morbilliviral antigen in neurons, neuroglia, and MGSCs. By contrast, CS and BOFDI cases had a lesser amount and more restricted morbilliviral antigen distribution. Only 4 Guiana dolphins (cases 1, 2, 9 and 11) exhibited viral antigen in the CNS, and the degree of staining was limited. Specifically, cases 1 and 11 had a few positive neurons (axon hillocks and soma), astrocytes, endothelial cells, pericytes, arteriolar smooth muscle cells, and rare circulating leukocytes in the cervical spinal cord, cerebellum, and cerebrum (Fig 2F). Case 9 had moderate labeling of cortical neurons underlying inflamed meninges with scattered labeling of lymphocytes and histiocytes. Case 2 had only scattered positive endothelial cells.

### Lymphoid system

**Fig 3:**
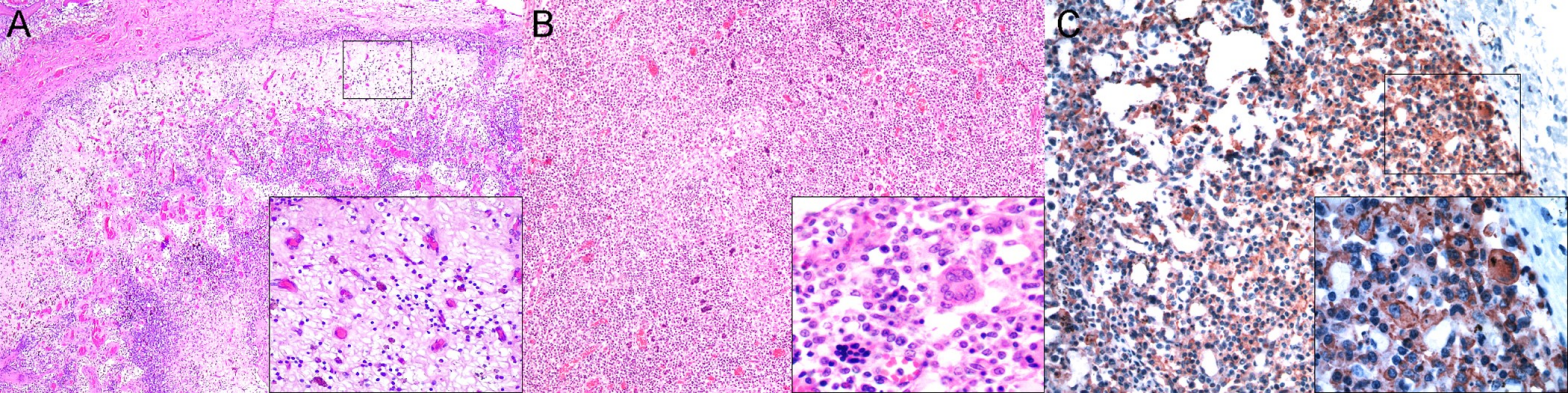
Cetacean morbillivirus (CeMV)-associated lesions in lymph nodes and spleen of striped dolphins (*Stenella coeruleoalba*), bottlenose dolphins (*Tursiops truncatus*), and Guiana dolphins (*Sotalia guianensis*). (A) Pulmonary lymph node (case 27). Severe cortical and paracortical lymphoid depletion. 40x. H&E. Inset: Higher magnification of the squared area in 3A depicting severe cortical lymphoid depletion. 200x. H&E. (B) Mediastinal lymph node (case 13). Paracortical expansion and numerous multinucleate giant cells/syncytia (MGCS). 40x. H&E. (C) Mediastinal lymph node (case 2). There is diffuse morbilliviral antigen in depleted cortex, paracortex, and sinusoidal leukocytes. 20x. IHC for CeMV. Inset: Diffuse morbilliviral labeling, including MGCS, at squared area in 3E. 400x. IHC for CeMV.

Most lymphoid tissues consistently evaluated (spleen, mediastinal, pulmonary, prescapular, and mesenteric lymph nodes) and those randomly sampled (paravertebral, pancreatic, gastrohepatic, and retroperitoneal/perirenal lymph nodes) had some CeMV-associated histopathologic findings (S6 and S7 Tables). In the lymph nodes, there was consistent lymphoid depletion. The cortex, primary and secondary follicles, and paracortex were more severely depleted than the medullary cords (Fig 3A), often with readily evident lymphocytolysis. Expansion or reactive hyperplasia of cortex and paracortex (Fig 3B and 3C), with or without a ‘starry-sky pattern’ and medullary cord hyperplasia and/or plasmacytosis, was seen in some cases from the Canary Islands and Italy, mainly with chronic infections. MGCS of various morphologies [25] were common, particularly in Guiana dolphins and one bottlenose dolphin (case 13), including those morphologically compatible with a follicular B-cell origin and those with an interfollicular and T cell-origin [25]. Eosinophilic lymphadenitis, characterized by nodules with necrotic centers and compatible with tracks made by migrating nematode larvae and fibrosis/fibrosclerosis were common findings, particularly in Guiana dolphins, but also in dolphins stranded in the Canary Islands and Italy.

In the spleen, lymphoid depletion was a consistent finding, particularly in Guiana dolphins. In general, the lymphoid follicles were more severely depleted than the periarteriolar lymphoid sheaths, often with readily evident lymphocytolysis. Sinus histiocytosis was a common finding in these cases, often accompanied by hemosiderosis, and less frequently, by erythrophagocytosis. A few cases displayed lymphoid reactive hyperplasia, mainly involving follicles, and fewer cases had splenitis.

CeMV-labeling was typically detected in follicular lymphocytes and follicular and interfollicular dendritic cells in lymph nodes and spleen, as well as in circulating lymphocytes and histiocytes in sinuses (Fig 3D, 3E and 3F). The viral antigen’s labeling abundance and intensity paralleled the severity and extent of lymphoid depletion. A few animals also had endothelial cell, pericyte, and/or smooth muscle labeling in occasional lymph node and splenic arterioles, more pronounced in GD-CeMV.

### Respiratory system

**Fig 4:**
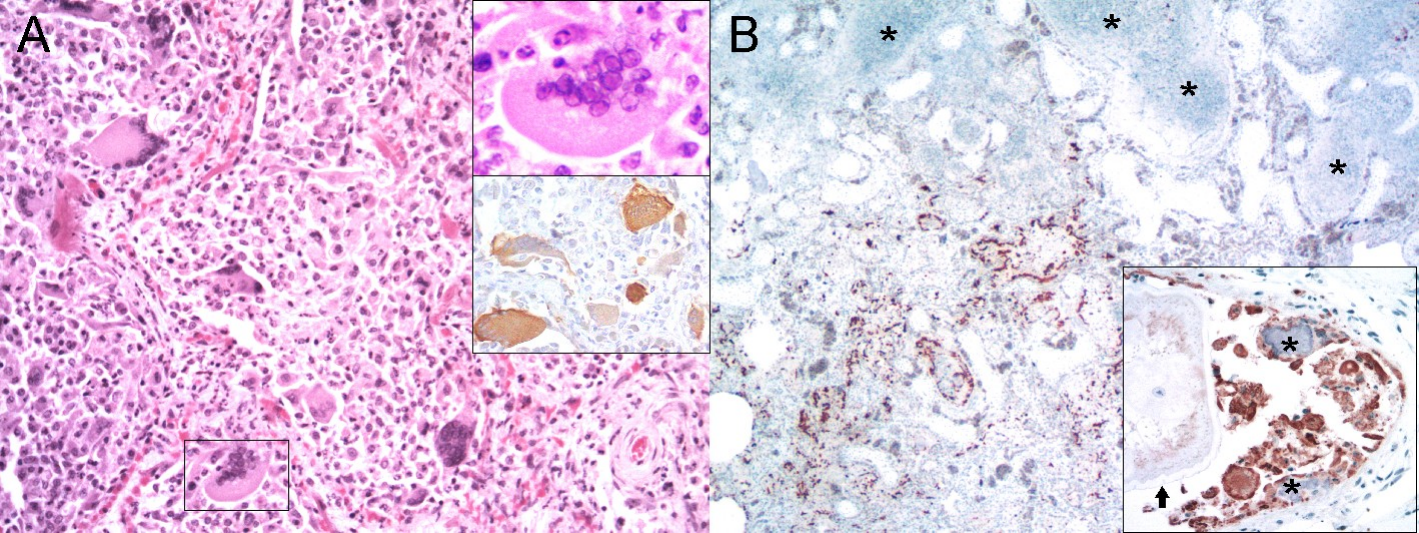
Cetacean morbillivirus-associated lesions and comorbidities in lung of striped dolphins (*Stenella coeruleoalba*), bottlenose dolphins (*Tursiops truncatus*) and Guiana dolphins (*Sotalia guianensis*). (A) Lung (Case 19). Detail of severe inflammation of alveolar spaces and septa with numerous multinucleate giant cells/syncytia (MGCS). Right upper inset: MGCS lining an alveolar septum. 400x. H&E. Right lower inset: pancytokeratin-positive MGCSs lining alveolar septa. 200x. IHC for AE1/AE3. (B) Lung (case 3). Morbilliviral labeling in areas adjacent to occlusive bronchiolar fungal masses (asterisks). 40x. IHC for CeMV. Inset: Epithelial and MGCS labeling in a bronchiole with lamina propria mineralization (asterisks) and luminal adult *H*. *brasiliensis* (arrow). 400x. IHC for CeMV.

There were CeMV-associated lung lesions in all dolphins but one striped dolphin (case 24), characterized by interstitial to bronchointerstitial pneumonia of varying severity and extent (Fig 4A, 4B and 4C). Guiana dolphins tended to have more severe lung lesions (S8 Table). Degenerative epithelial changes were common in bronchi, bronchioles, and alveoli. Inflammatory infiltrates composed of lymphocytes, plasma cells, and macrophages were common in alveolar septa, submucosae, and interstitium, together with fibrosis and varying degrees of parenchymal remodeling (scarring) and sclerosis of airways (Fig 4C). The latter were particularly severe in Guiana dolphins. Alveolar exudates were mainly composed of macrophages and neutrophils, which varied depending on concurrent etiologies. MGCS of various morphologies [25] and viral INCIBs were seen in some animals and typically coexisted. Most lung MGCS were AE1/AE3-positive (Inset in Fig 4A). Case 19 recapitulated features of ‘giant cell pneumonia’ (Fig 4A) [26]. Preexisting pulmonary disease and opportunistic infections were common. The former included verminous pneumonia by *H*. *brasiliensis* in Guiana dolphins and *Stenurus* spp. and *Halocercus* spp. in striped dolphins and bottlenose dolphins. Secondary lung infections included unidentified bacteria, mainly in striped dolphins stranded in Italy and the Canary Islands. In case 25, bacteriological analyses identified coagulase-positive *Staphylococcus aureus* in lung, liver, urine, and brain, confirming septicemia [3]. Pulmonary coinfections by hyphate fungal agents suggestive of *Mucorales* were found in two Guiana dolphins (case 3 and 11) and one striped dolphin (case 27). Additional non-specific findings were patchy atelectasia and emphysema, as well as bronchial/bronchiolar sphincter constriction with/without smooth muscle hypertrophy/hyperplasia, submucosal fibrosis, and dystrophic mineralization of the lamina propria.

Morbilliviral antigen immunolabeling was observed in type I and II pneumocytes and MGCS (Fig 4D and 4E) of most cases, as well as in alveolar and interstitial macrophages, and to a lesser extent in intravascular/circulating monocytes and lymphocytes. In some cases, viral antigen was also detected in endothelial cells. Although the trachea was underrepresented in the sample set, viral antigen immunolabeling was noted in case 2 (Fig 4F).

### Causes of stranding and/or death

In four animals known to have live-stranded (cases 12, 13, 14, and 25), ‘stranding stress response (SSR)’ or ‘capture myopathy-like’-associated vascular, muscular, and presumed acute renal dysfunction likely contributed to death. Eight of 23 animals found dead had gross and/or histopathologic findings suggestive of SSR, so this could have played a role in these cases as well. Taking all gross and microscopic lesions together, the most plausible cause of stranding and/or death in most animals was considered to be systemic/localized CeMV infection and comorbidities, with major involvement of the CNS, lung, and/or lymphoid system. Impairment of vital CNS functions and/or cardio-respiratory failure could reasonably explain stranding and/or death in most animals [1,2,3,14,15]. Entanglement was likely a major contributor to death in case 14.

## Discussion

We have provided a comprehensive and comparative analysis of the results of histopathologic and viral immunohistochemical investigations on CeMV-associated pathology in GD-CeMV-infected Guiana dolphins from Southwestern Atlantic (Brazil) and DMV-infected striped dolphins and bottlenose dolphins from the Northeast-Central Atlantic (Canary Islands, Spain) and Western Mediterranean Sea (Italy). There are remarkable pathologic similarities between infections by terrestrial mammal morbilliviruses and CeMV, and much of the current knowledge on CeMV originated from elegant comparative studies of MeV and CDV [3,14,15,27,28,29], the two most studied morbilliviruses in humans and dogs, respectively. Rinderpest virus (RPV) and Peste des petits ruminants virus (PPRV) infections are similar to CeMV infection, but they typically lack neurotropism [30].

DMV showed remarkable neurotropism in striped dolphins and bottlenose dolphins, in contrast to GD-CeMV in Guiana dolphins. Only two Guiana dolphins had findings suggestive of CeMV infection in the brain, while 4 exhibited some morbilliviral antigen labeling in the CNS. In these cases, there were scattered positive neurons and rare positive astrocytes and arteriolar endothelial cells, pericytes, and/or smooth myocytes in the cervical spinal cord, cerebellum, and cerebrum, as well as rare circulating leukocytes. These findings suggest leukocyte trafficking and blood brain barrier circumvention as a possible route of entry for CeMV into the brain, a phenomenon that remains largely unknown in cetaceans. Lack of neurodegenerative, reactive, or inflammatory changes in most Guiana dolphins suggests low neurovirulence of this strain in this cohort of animals; however, it is conceivable that CNS involvement occurs at a later stage of infection and that these animals succumbed too early for CNS pathology to be observed. Host-related and pathogen-related determinants, along with environmental factors, could influence GD-CeMV neurotropism in Guiana dolphins. It is possible that all morbilliviruses transiently infect the CNS in their natural hosts, but development of disease is dependent on the efficiency of the immune response, abundance of receptors, and viral replication and perpetuation mechanisms [30].

We detected CNS lesions as part of the currently proposed AS, SS, CS, and BOFDI forms [1] in striped dolphins and bottlenose dolphins infected with DMV [2,3,19,20,21]. ‘Canonical’ AS infections in striped dolphins and bottlenose dolphins (cases 14, 15, 19, 21, 23, and 26) included degenerative and reactive changes of neurons and neuroglia, along with varying degrees of inflammation in both the gray and white matter. Current ‘canonical’ SS cases comprised those with some evident CeMV-identifiable lesions complicated by secondary infections, namely suspected brucellosis (case 13) [31], and confirmed coagulase-positive *Staphylococcus aureus* infection (case 25), and localized or disseminated mycosis (cases 3, 11, and 27). In these cases, CeMV-pulmonary, -lymphoid, and -CNS attributable lesions did not differ from systemic acute cases, although they were variably obscured by the inflammatory response to the concomitant pathogens [1]. No demyelinating meningoencephalitis was noted in any of these cases, which differs from previous studies [1]. We believe that the SS disease category remains poorly delineated, as most if not all of its features may overlap with acute and chronic presentations [1]. A better definition of subacute cases will require deeper knowledge of the disease, specifically of the host immune response, including viremia and seroconversion profiling.

There is also some controversy in the classification of CS disease cases. Histopathologic, IHC, and/or PCR findings in cases that were argued as prototypical of chronic systemic CeMV infections [16,32,33] did not differ from suggested subacute cases [1]. In our study, we believe cases 12, 18, 22, and 24 represent CS cases based on histopathologic, immunohistochemical, and/or PCR results [1]. Specifically, there was scattered morbilliviral labeling by IHC in lung (cases 12 and 18), as well as restricted molecular positivity in lung (cases 12, 18, 22, and 24) and brain (cases 12, 18, and 22).

Presumably less controversy exists regarding BOFDI cases, where by definition there are no extraneural lesions nor viral antigen and/or genome attributable to CeMV [4]. This is probably the less prevalent form of CeMV infection worldwide, apart from the still cryptic ‘subclinical’ cases [34,35,36] and those involving ‘CeMV non-susceptible’ species [37]. The chronology of lesions in reported cetacean BOFDI-affected striped dolphins is not clear, yet the various reports indicate perivascular cuffing, diffuse gliosis, and glial nodules with neuronophagia as the most consistent findings [2,3,4,5]. A recent study has identified a prominent colonization of calbindin (CALB)-immunoreactive (IR) neurons and, to a lesser extent, also of nitric/nitrous oxide synthase (NOS)-IR neurons in the brain tissue from BOFDI-affected striped dolphins [38]. That study also showed a very limited involvement of astrocytes, despite the presence of astrogliosis/astrocytosis. In the present study, cases 16, 17, and 20 had CNS histopathologic findings and extraneural IHC/PCR results that support a BOFDI diagnosis. We observed chiefly lymphoplasmacytic perivascular cuffing with occasional meningitis, subjective neuron loss, and reactive neurogliosis in these cases. In case 17, however, metencephalic and myelencephalic lesions were more severe and extensive than prosencephalic ones, which diverges from previous BOFDI reports [4]. Cases 16 and 20 had only the cerebrum and thalamus evaluated, so further conclusions cannot be drawn. In acute CeMV cases, gray and white matter involvement predominated, whereas chronic cases tended to have most changes in the gray matter. For the most part, these results are largely in agreement with previous observations in striped dolphins and bottlenose dolphins [1].

The pathology of CNS infection by both MeV and CDV is quite variable [30]. In humans, three MeV-CNS complications with relatively low incidence are acute disseminated encephalomyelitis (ADEM), measles inclusion body encephalitis (MIBE), and subacute sclerosing panencephalitis (SSPE) [39,40]. The pathogenesis of these entities is variable, including viral invasion of brain via infected lymphocytes, autoimmune molecular mimicry, T-cell dysfunction, and persistent slow virus infection with mutated virus. These conditions may have neuroanatomical predilection sites, and there is considerable overlap in histopathology between them. In most instances, the pathologic picture is conditioned by epidemiologic information. Thus, these neuropathologic findings have clinical connotations that are difficult if not impossible to ascertain in CeMV-infected free-ranging cetaceans.

In dogs, CDV-CNS disease may show distinctive manifestations with variable incidences: 1) a major gray matter tropism as acute fulminant encephalopathy and encephalitis (AFE-E) often targeting the cerebellar and cerebral cortex in experimentally infected, young, and immunologically naïve dogs; 2) post-vaccinal encephalitis (PVE), largely a necrotizing polioencephalitis involving the cerebral cortex and caudal brainstem and characterized by neuronal necrosis, mononuclear perivascular cuffs, and occasional neuronal INCIBs [29,41]; 3) old dog encephalitis (ODE), believed to occur in immunocompetent dogs with neuronal persistence of replication-defective virus [29,41] and involving primarily a gray matter distribution pattern [42]; 4) inclusion body polioencephalitis (IBP), which occurs in dogs that are neither aged or have a history of recent vaccination [43]; and 5) subacute to chronic CDV-CNS infection leading to demyelinating leukoencephalitis (CDV-DL) [29,44].

We observed that striped dolphins and bottlenose dolphins shared neuropathologic features with MIBE, IBP, and SSPE/ODE, but not ADEM, AFE-E, PVE, and CDV-DL. Furthermore, lesions in AS/SS and CS showed interindividual variation. AS/SS cases tended to have more neurodegenerative alterations coupled with more abundant and widely distributed morbilliviral antigen. BOFDI cases had relatively milder inflammation and little evident neurodegenerative alterations. All Guiana dolphins from Rio de Janeiro (Brazil) included in this study died during a 5-month UME that claimed more than 250 animals [8], which further supports the acute/subacute systemic nature of lesions observed. None of the animals from the Canaries belonged to an epizootic, so they may be interpreted as ‘endemic’ cases. Regarding the Italian Mediterranean specimens, only cases 22, 23, and 24 represented epidemic fatalities, while the remaining cases were thought to be interepizootic or ‘endemic’ cases.

In cetaceans, CeMV-associated lesions and antigen distribution are relatively well-characterized in prosencephalic and metencephalic regions, but less so in diencephalic and myelencephalic locations [1,3,4,14,15,45]. Morbilliviral antigen was more frequently detected in the cytoplasm and nuclei of neurons, astrocytes, and oligodendrocytes of the cerebrum, cerebellum, thalamus, caudal brainstem, and spinal cord. These results are largely in agreement with previous studies [2,3,4,5,14,15,20,21,46]. This study is the first to employ double IHC labeling to address the origin of neuroglial cells harboring morbilliviral antigen in FFPE cetacean tissues. There is a lack of data on oligodendrocytic involvement in CeMV. The role of oligodendrocytes in MeV and CDV neuropathogenesis remains unclear, as studies have shown variable results [47,48,49]. CDV is believed to cause early axonal damage followed by (secondary) demyelination and subsequent axon-myelin-glia disturbances. Also, a major role for p75 neurotrophin (NTR)-positive bipolar cells during CDV-DL has been postulated [44]. These hypotheses remain to be proven in cetaceans. This study found frequent axonal alterations, mainly swelling and some loss, suggesting a pathogenetic role in CNS-CeMV infection, as reported in early CNS-CDV infection [29]. Our findings in this cohort of animals do not support demyelination as a major pathologic feature, either in acute or chronic lesions, including BOFDI cases. A limitation in this study was the use of S-100 as a marker for oligodendrocytes; future studies may use a more specific marker, such as oligodendrocyte transcription factor (Olig 2) and dual IHC. Also, dual IHC with neuron and microglia immunomarkers may prove of value for better characterizing neuropathogenetic aspects of CeMV.

We found consistent pneumotropism of DMV and GD-CeMV. In humans, two major MeV-pneumonia patterns are recognized [26]: a) ‘giant cell or Hecht pneumonia’, and b) interstitial to bronchointerstitial pneumonia. The latter is also the most common in canine distemper [41]. In giant cell pneumonia, there is intense alveolar damage, abundant MGCS, and type I pneumocyte necrosis, while interstitial to bronchointerstitial pneumonia is characterized by septal inflammatory infiltrates, rare giant cells, and little disturbances in alveolar epithelium with only rare pneumocyte necrosis, [26]. Common additional findings include peribronchial mononuclear cell infiltrates, proliferation of type II pneumocytes, formation of hyaline membranes, and squamous metaplasia of bronchial epithelium [26]. Most animals in this study featured interstitial to bronchointerstitial pneumonia with or without INCIBs in MGCS and epithelial cells. Only case 19 had lesions that fit the description of ‘giant cell pneumonia’[50]. Interpretation of pneumonia patterns was complicated by preexisting and/or secondary infections. Almost all Guiana dolphins and most striped and bottlenose dolphins had gross and/or histologic evidence of lungworms, most likely acquired prior to CeMV infection. Verminous pneumonia by *H*. *brasiliensis* was particularly extensive and severe in Guiana dolphins, and it was likely a major contributor to morbidity and mortality in these cases.

Coinfections in CeMV are common but not well characterized [1]. Pulmonary bacteria were relatively common in our cases, but their identity was only known in a Guiana dolphin (case 11) with *Escherichia coli* isolated from bronchial exudate and a bottlenose dolphin (case 25) with confirmed systemic *S*. *aureus* [3,51]. Two Guiana dolphins and one striped dolphin had severe concomitant fungal pneumonia morphologically compatible with *Mucorales* or possibly *Entomophthorales* [52]. Many questions remain about the interactions between bacteria, viruses, fungi, and CeMV in the development of pneumonia. The consistent severe and extensive lesions with the presence of intrapulmonary morbilliviral antigen lend support to major respiratory transmission, particularly in systemic acute/subacute cases. This route of infection was likely a major factor in the recent UME in Rio de Janeiro.

MeV-lymphadenitis may occur in the course of measles infection or days to weeks after vaccination [53]. In naïve humans and nonhuman primates (NHPs), there is diffuse paracortical immunoblastic hyperplasia with relative depletion of small lymphocytes at earlier stages [53]. The most commonly infected cell type may be B lymphocytes in lymphoid follicles, rapidly followed by follicular dendritic cells [54]. There may also be acute inflammation and necrosis, and secondary lymphoid follicles may be absent or poorly developed. These lesions result in immunosuppression [55]. In all three dolphin species, we observed consistent CeMV-associated alterations in nearly all lymphoid tissues examined, including the spleen and mediastinal, pulmonary, prescapular, and mesenteric lymph nodes. The most consistent finding was mild to marked lymphoid depletion, most prominent in Guiana dolphins. In the lymph nodes, lymphoid depletion was most prominent in the cortex and the paracortex and was often associated with lymphocytolysis. Lymphoid depletion appeared less pronounced in the medullary cords. These features are similar to findings in MeV and CDV infections [29,53,56], as well as previously reported CeMV infections [14,15].

MGCS, often referred to as ‘Warthin-Finkeldey cells’ (WFCs), usually appear in the prodromal phase of measles in hyperplastic primary, secondary, and tertiary lymphoid tissues [53]. In nonhuman primates, four types of MGCS have been recognized, with chronological implications: WFC, reticular, phagocytic, and plasma cell type [57]. In this study, we observed MGCS of various morphologies, often with INCIBs [25]. WFCs bear morphologic and immunophenotypic heterogeneity [25]. A clear-cut distinction between MGCS in CeMV infection requires further IHC analysis, but we observed those cytomorphologically compatible with follicular B-cell origin and interfollicular T cell-origin [25]. Reactive hyperplasia of the paracortex, cortex, and medullary cords, including occasional plasmacytosis, was noted in lymph nodes of a few dolphins, mainly in striped dolphins from the Canary Islands. The potential implications of these findings in understanding the chronology of CeMV infection will require further studies [57].

Many questions remain unanswered regarding immunosuppression in CeMV. In distemper, the diminished immune function in the early phase of the disease is associated with viremia and lysis of lymphocytes and macrophages. Immunosuppression during the acute phase of infection is accompanied by a lack of recirculating T lymphocytes in the marginal sinus of lymph nodes, and morphological alterations of the immune system are partially reversed by repopulation and germinal center formation of lymphoid tissues in persistently infected and convalescent dogs, respectively [29]. These events remain to be demonstrated in CeMV infection. Although the chronology of CeMV is unknown, the apparent zonal distribution of depletive phenomena correlated with morbilliviral antigen abundance and labeling intensity.

In addition to CeMV-associated lymphadenitis, nodular to diffuse eosinophilic lymphadenitis, suggestive of larval nematode migration tracts was a common finding, particularly in Guiana dolphins. Parasitic lymphadenitis was also seen in striped dolphins and bottlenose dolphins. Prevalence, chronicity, and severity of parasitic lymphadenitis in these cases paralleled verminous pneumonic lesions, and most of these animals had systemic endoparasitism. Studies have shown that cytokine imbalance in TH1 and TH2 immune responses play a major role in disease susceptibility and progression in MeV- and CDV-infected individuals, and hosts with TH2 deviated immune responses have more severe disease. The comparative histopathologic results in this study lend support to the hypothesis that preexisting TH2-predominant responses associated with systemic parasitosis, most prominent in lungs and lymph nodes, could have rendered some animals, particularly Guiana dolphins, more susceptible to CeMV. This hypothesis will be better addressed by ongoing IHC and cytokine gene expression analyses.

In the spleen, the most consistent CeMV-associated finding was lymphoid depletion, which was most pronounced in Guiana dolphins. In general, the lymphoid follicles were more severely depleted than periarteriolar lymphoid sheaths. Lymphocytolysis was less common in the spleen than in lymph nodes. These changes paralleled morbilliviral antigen immunolabeling, which involved follicular lymphocytes and dendritic cells, circulating lymphocytes, and histiocytes in sinuses.

A variety of pathologic findings unrelated to CeMV infection was seen in this cohort of animals. Opportunistic infections likely contributed to stranding and/or death in all these animals, and while a thorough discussion of these is out of the scope of the study, examples of these coexisting disease processes have been reported in the literature [3,14,15].

This study did not investigate the effects of chemical pollutants on the immune function of these animals, but further studies should integrate this information in order to better delineate immune status in animals with CeMV. A few animals included in this study had known high levels of certain pollutants per parallel studies, e.g. heavy metals in dolphins stranded in Italy, and elevated blubber and hepatic levels of polychlorinated biphenyls, organochlorides pesticides, polycyclic aromatic hydrocarbons, persistent organic pollutants, mercury, and selenium in dolphins stranded in the Canary Islands [58,59]. Future studies on CeMV may benefit from integrative eco-toxico-pathologic analyses with individual and population level emphasis [60].

CeMV is showing a steady host and geographic range expansion, but precise measurements of CeMV incidence and mortality are lacking [1,8,46]. There is almost a complete lack of clinico-epidemiological information on most CeMV-infected dolphins [37], so determining the precise chronology and progression of pathologic changes is difficult. Although chronological data for CeMV infection in these animals is not available, our findings agree with the generally accepted pathophysiological mechanisms described for MeV and CDV infections [28].

In conclusion, our results suggest CeMV-AP may vary depending on virulence of virus strain and host species. The host age and immune status also likely play important roles. We observed pneumotropism and widespread lymphohistiocytic tropism of DMV in striped dolphins and bottlenose dolphins, as well as of GD-CeMV in Guiana dolphins. DMV infections showed remarkable neurotropism, characterized by meningoencephalitis, in contrast to GD-CeMV infections. The lymphoid system was involved in all three species, with consistent lymphoid depletion. Multinucleate giant cells/syncytia and characteristic viral inclusion bodies were variably observed in these organs. Overall, there was widespread lymphohistiocytic, epithelial, and neuronal/neuroglial CeMV-antigen detection with some individual, host species, and CeMV strain differences. Preexisting and opportunistic infections were common, particularly endoparasitism, followed by bacterial, fungal, and viral infections, and in certain cases contributed significantly to stranding and/or demise. These results lend support to the hypothesis that preexisting TH2-predominant responses could have rendered these dolphins, particularly Guiana dolphins, more susceptible to CeMV-infection. Thefindings also contribute to understanding convergences and divergences of CeMV infections between CeMV strains, hosts, and different geographic locations, thereby setting the basis for future neuro-immunopathological and neuro-immunopathogenetic comparative investigations.

## Supporting information

S1 Fig Bottlenose dolphin (*Tursiops truncatus*) brain; Mediosagittal aspect. A) Areas for neuroanatomical sampling (indicated as black rectangles) in striped dolphins (*Stenella coeruleoalba*) and bottlenose dolphins from the Canary Islands (Spain). AcL, anterior cerebellar lobe; H, hypothalamus; Hy, hypophysis; Met, metencephalon; My, myelencephalon; OrL, orbital lobe; oc, optic chiasm; OccL, occipital lobe; PcL, posterior cerebellar lobe; PL, parietal lobe; T, thalamus; TL, temporal lobe; Ve, vermis. Brain diagram adapted from Oelschläger, H. & Oelschläger, J.S. (2009).
(TIF)

S1 Table Template for recording histopathological findings in the central nervous system, including prosencephalon, mesencephalon and rhombencephalon. (DOCX)

S2 Table Template for recording histopathological findings in the respiratory system. (DOCX)

S3 Table Template for recording histopathological findings in the lymphoid system (lymph nodes, spleen). (DOCX)

S4 Table Gross and microscopic pathologic findings, and most probable cause(s) of stranding and/or death (COD) in Guiana dolphins (*Sotalia guianensis*), striped dolphins (*Stenella coeruleoalba*) and bottlenose dolphins (*Tursiops truncatus*) included in this study. (DOCX)

S5 Table Main microscopic findings in central nervous system of striped dolphins (*Stenella coeruleoalba*) and bottlenose dolphins (*Tursiops truncatus*) from Canary Islands (Spain) and Italy, and Guiana dolphins (*Sotalia guianensis*) from Brazil. (DOCX)

S6 Table Main microscopic findings in prescapular, pulmonary, mediastinal and mesenteric lymph nodes of striped dolphins (*Stenella coeruleoalba*) and bottlenose dolphins (*Tursiops truncatus*) from Canary Islands (Spain) and Italy, and Guiana dolphins (*Sotalia guianensis*) from Brazil. (DOCX)

S7 Table Main microscopic findings in spleen of striped dolphins (*Stenella coeruleoalba*) and bottlenose dolphins (*Tursiops truncatus*) from Canary Islands (Spain) and Italy, and Guiana dolphins (*Sotalia guianensis*) from Brazil. (DOCX)

S8 Table Main microscopic findings in the respiratory system of striped dolphins (*Stenella coeruleoalba*) and Atlantic bottlenose dolphins (*Tursiops truncatus*) from the Canary Islands (Spain) and Italy, and Guiana dolphins (*Sotalia guianensis*) from Brazil. (DOCX)

## References

[1] Van Bressem MF, Duignan PJ, Banyard A, Barbieri M, Colegrove KM, De Guise S, et al Cetacean morbillivirus: current knowledge and future directions. Viruses. 2014; 6 (12): 5145–5181. 10.3390/v6125145 25533660 PMC4276946

[2] Di Guardo G, Cocumelli C, Scholl F, Di Francesco CE, Speranza R, Pennelli M, et al Morbilliviral encephalitis in a striped dolphin *Stenella coeruleoalba* calf from Italy. Dis Aquat Organ. 2011; 95 (3): 247–251. 10.3354/dao02355 21932537

[3] Di Guardo G, Di Francesco CE, Eleni C, Cocumelli C, Scholl F, Casalone C, et al Morbillivirus infection in cetaceans stranded along the Italian coastline: pathological, immunohistochemical and biomolecular findings. Res Vet Sci. 2013; 94 (1): 132–137. 10.1016/j.rvsc.2012.07.030 22921372

[4] Domingo M, Vilafranca M, Visa J, Prats N, Trudgett A, Visser I. Evidence for chronic morbillivirus infection in the Mediterranean striped dolphin (*Stenella coeruleoalba*). Vet Microbiol. 1995; 44 (2–4): 229–239. 8588317 10.1016/0378-1135(95)00016-4

[5] Soto S, Alba A, Ganges L, Vidal E, Raga JA, Alegre F, et al Post-epizootic chronic dolphin morbillivirus infection in Mediterranean striped dolphins *Stenella coeruleoalba*. Dis Aquat Organ. 2011; 96 (3): 187–194. 10.3354/dao02387 22132497

[6] Groch KR, Colosio AC, Marcondes MC, Zucca D, Diaz-Delgado J, Niemeyer C, et al Novel cetacean morbillivirus in Guiana dolphin, Brazil. Emerg Infect Dis. 2014; 20 (3): 511–513. 10.3201/eid2003.131557 24565559 PMC3944878

[7] Shimizu Y, Ohishi K, Suzuki R, Tajima Y, Yamada T, Kakizoe Y, et al Amino acid sequence variations of signaling lymphocyte activation molecule and mortality caused by morbillivirus infection in cetaceans. Microbiol Immunol. 2013; 57 (9): 624–632. 10.1111/1348-0421.12078 23815475

[8] Groch KR, Santos-Neto EB, Diaz-Delgado J, Ikeda JMP, Carvalho RR, Oliveira RB, et al Guiana dolphin unusual mortality event and link to cetacean morbillivirus, Brazil. Emerg Infect Dis. 2018; 24 (7): 1349–1354. 10.3201/eid2407.180139 29912687 PMC6038766

[9] von Messling V, Springfeld C, Devaux P, Cattaneo R. A ferret model of canine distemper virus virulence and immunosuppression. J Virol. 2003; 77 (23): 12579–12591. 10.1128/JVI.77.23.12579-12591.2003 14610181 PMC262577

[10] Yanagi Y, Takeda M, Ohno S. Measles virus: cellular receptors, tropism and pathogenesis. J Gen Virol. 2006; 87 (Pt 10): 2767–2779. 10.1099/vir.0.82221-0 16963735

[11] Nielsen L, Sogaard M, Jensen TH, Andersen MK, Aasted B, Blixenkrone-Moller M. Lymphotropism and host responses during acute wild-type canine distemper virus infections in a highly susceptible natural host. J Gen Virol. 2009; 90 (Pt 9): 2157–2165. 10.1099/vir.0.010744-0 19494053

[12] Kennedy S, Smyth JA, Cush PF, McCullough SJ, Allan GM, McQuaid S. Viral distemper now found in porpoises. Nature. 1988; 336: 6194. 10.1038/336021a0 3185717

[13] Di Guardo G, Agrimi U, Morelli L, Cardeti G, Terracciano G, Kennedy S. Post-mortem investigations on cetaceans found stranded on the coasts of Italy between 1990 and 1993. Vet Rec. 1995; 136 (17): 439–442. 7631479 10.1136/vr.136.17.439

[14] Domingo M, Visa J, Pumarola M, Marco AJ, Ferrer L, Rabanal R, et al Pathologic and immunocytochemical studies of morbillivirus infection in striped dolphins (*Stenella coeruleoalba*). Vet Pathol. 1992; 29 (1): 1–10. 10.1177/030098589202900101 1557861

[15] Duignan PJ, Geraci JR, Raga JA, Calzada N. Pathology of morbillivirus infection in striped dolphins (*Stenella coeruleoalba*) from Valencia and Murcia, Spain. Can J Vet Res. 1992; 56 (3): 242–248. 1423061 PMC1263546

[16] Lipscomb TP, Schulman FY, Moffett D, Kennedy S. Morbilliviral disease in Atlantic bottlenose dolphins (*Tursiops truncatus*) from the 1987–1988 epizootic. J Wildl Dis. 1994; 30 (4): 567–571. 10.7589/0090-3558-30.4.567 7760492

[17] Di Guardo G, Mazzariol S. Cetacean Morbillivirus-Associated Pathology: Knowns and Unknowns. Front Microbiol. 2016; 7: 112 10.3389/fmicb.2016.00112 26903991 PMC4744835

[18] Geraci JR, Lounsbury VJ.Marine Mammals Ashore: a field guide for strandings. Baltimore, MD: National Aquarium in Baltimore; 2005.

[19] Centelleghe C, Beffagna G, Zanetti R, Zappulli V, Di Guardo G, Mazzariol S. Molecular analysis of dolphin morbillivirus: A new sensitive detection method based on nested RT-PCR. J Virol Methods. 2016; 235: 85–91. 10.1016/j.jviromet.2016.05.005 27220282

[20] Sierra E, Sánchez S, Saliki JT, Blas-Machado U, Arbelo M, Zucca D, et al Retrospective study of etiologic agents associated with nonsuppurative meningoencephalitis in stranded cetaceans in the Canary Islands. J Clin Microbiol. 2014; 52 (7): 2390–2397. 10.1128/JCM.02906-13 24759718 PMC4097689

[21] Sierra E, Zucca D, Arbelo M, Garcia-Alvarez N, Andrada M, Deniz S, et al Fatal systemic morbillivirus infection in bottlenose dolphin, canary islands, Spain. Emerg Infect Dis. 2014; 20 (2): 269–271. 10.3201/eid2002.131463 24447792 PMC3901504

[22] Barrett T, Visser IKG, Mamaev L, Goatley L, Bressem M-F, Osterhaus ADME. Dolphin and porpoise morbilliviruses are genetically distinct from phocine distemper virus. Virology. 1993; 193 (2): 1010–1012. 8460473 10.1006/viro.1993.1217

[23] Frisk AL, König M, Moritz A, Baumgärtner W. Detection of canine distemper virus nucleoprotein RNA by reverse transcription-PCR using serum, whole blood, and cerebrospinal fluid from dogs with distemper. J Clin Microbiol. 1999; 37 (11): 3634–3643. 10523566 10.1128/jcm.37.11.3634-3643.1999 PMC85712

[24] Grant RJ, Banyard AC, Barrett T, Saliki JT, Romero CH. Real-time RT-PCR assays for the rapid and differential detection of dolphin and porpoise morbilliviruses. J Virol Methods. 2009; 156 (1–2): 117–123. 10.1016/j.jviromet.2008.11.008 19084557

[25] Nozawa Y, Ono N, Abe M, Sakuma H, Wakasa H. An immunohistochemical study of Warthin-Finkeldey cells in measles. Pathol Int. 1994; 44 (6): 442–447. 8055110 10.1111/j.1440-1827.1994.tb01708.x

[26] Moussallem TM, Guedes F, Fernandes ER, Pagliari C, Lancellotti CLP, de Andrade HF, et al Lung involvement in childhood measles: severe immune dysfunction revealed by quantitative immunohistochemistry Hum pathol. 2007; 38 (8): 1239–1247. 10.1016/j.humpath.2007.01.015 17499339

[27] Ciaccio C. Beitrag zur pathologischen Anatomie und zur Mikrobiologie der Masern. Virchows Arch A Pathol Anat Histopathol. 1910; 199: 378.

[28] Moss WJ. Measles. The Lancet. 2017; 390 (10111): 2490–2502. 10.1016/S0140-6736(17)31463-0 28673424

[29] Beineke A, Puff C, Seehusen F, Baumgärtner W. Pathogenesis and immunopathology of systemic and nervous canine distemper. Vet Immunol Immunopathol. 2009; 127 (1–2): 1–18. 10.1016/j.vetimm.2008.09.023 19019458

[30] Cosby SL, Duprex WP, Hamill LA, Ludlow M, McQuaid S. Approaches in the understanding of morbillivirus neurovirulence. J Neurovirol. 2002; 8 (Suppl 2): 85–90. 12491157 10.1080/13550280290167975

[31] Guzman-Verri C, Gonzalez-Barrientos R, Hernandez-Mora G, Morales JA, Baquero-Calvo E, Chaves-Olarte E, et al *Brucella ceti* and brucellosis in cetaceans. Front Cell Infect Microbiol. 2012; 2: 3 10.3389/fcimb.2012.00003 22919595 PMC3417395

[32] Stephens N, Duignan PJ, Wang J, Bingham J, Finn H, Bejder Ls, et al Cetacean morbillivirus in coastal Indo-Pacific bottlenose dolphins, Western Australia. Emerg Infect Dis. 2014; 20 (4): 666–670. 10.3201/eid2004.131714 24656203 PMC3966363

[33] Taubenberger JK, Tsai M, Krafft AE, Lichy JH, Reid AH, Schulman FY, et al Two morbilliviruses implicated in bottlenose dolphin epizootics. Emerg Infect Dis. 1996; 2 (3): 213–216. 10.3201/eid0203.960308 8903232 PMC2626800

[34] Reidarson TH, McBain J, House C, King DP, Stott JL, Krafft A, et al Morbillivirus infection in stranded common dolphins from the Pacific Ocean. J Wildl Dis. 1998; 34 (4): 771–776. 10.7589/0090-3558-34.4.771 9813847

[35] Bossart GD, Reif JS, Schaefer AM, Goldstein J, Fair PA, Saliki JT. Morbillivirus infection in free-ranging Atlantic bottlenose dolphins (*Tursiops truncatus*) from the Southeastern United States: seroepidemiologic and pathologic evidence of subclinical infection. Vet Microbiol. 2010; 143 (2–4): 160–166. 10.1016/j.vetmic.2009.11.024 20005646

[36] Canturri A, Cuvertoret M, Pérez L, Ganges L, Jeffers A, McMenamy MJ, et al Cetacean morbillivirus infection and central nervous system aspergillosis in absence of histopathologic morbilliviral lesions and immunohistochemical CeMV-labelling in the Mediterranean striped dolphin (*Stenella coeruleoalba*). J Comp Pathol. 2018; 158: 113.

[37] Van Elk CE, Van de Bildt MWG, Jauniaux T, Hiemstra S, Van Run PRWA, Foster G, et al Is dolphin morbillivirus virulent for white-beaked dolphins (*Lagenorhynchus albirostris*)? Vet Pathol. 2014; 51 (6): 1174–1182. 10.1177/0300985813516643 24399208

[38] Luca R, Giacominelli-Stuffler R, Mazzariol S, Roperto S, Cocumelli C, G DIG. Neuronal and astrocytic involvement in striped dolphins (*Stenella coeruleoalba*) with morbilliviral encephalitis. Acta virol. 2017; 61 (4): 495–497. 10.4149/av_2017_414 29186969

[39] Griffin DE. Measles virus and the nervous system. Handbook of Clinical Neurology. 2014; 123: 577–590. 10.1016/B978-0-444-53488-0.00027-4 25015505

[40] Bitnun A, Shannon P, Durward A, Rota PA, Bellini WJ, Graham C, et al Measles inclusion-body encephalitis caused by the vaccine strain of measles virus. Clin Infect Dis. 1999; 29 (4): 855–861. 10.1086/520449 10589903

[41] Greene CE, Vandevelde M. Canine Distemper In: Greene CE, editor. Infectious Diseases of the Dog and Cat-E-Book Elsevier Health Sciences; 2013 pp. 25–42.

[42] Summers BA, Appel MJ. Aspects of canine distemper virus and measles virus encephalomyelitis. Neuropathol Appl Neurobiol. 1994; 20 (6): 525–534. 7898614 10.1111/j.1365-2990.1994.tb01006.x PMC7194305

[43] Nesseler A, Baumgartner W, Gaedke K, Zurbriggen A. Abundant expression of viral nucleoprotein mRNA and restricted translation of the corresponding viral protein in inclusion body polioencephalitis of canine distemper. J Comp Pathol. 1997; 116 (3): 291–301. 9147247 10.1016/s0021-9975(97)80004-7

[44] Lempp C, Spitzbarth I, Puff C, Cana A, Kegler K, Techangamsuwan S, et al New aspects of the pathogenesis of canine distemper leukoencephalitis. Viruses. 2014; 6 (7): 2571–2601. 10.3390/v6072571 24992230 PMC4113784

[45] Uchida K, Muranaka M, Horii Y, Murakami N, Yamaguchi R, Tateyama S. Non-purulent meningoencephalomyelitis of a Pacific striped dolphin (*Lagenorhynchus obliquidens*). The first evidence of morbillivirus infection in a dolphin at the Pacific Ocean around Japan. J Vet Med Sci. 1999; 61 (2): 159–162. 10081755 10.1292/jvms.61.159

[46] Sierra E, Fernández A, Zucca D, Câmara N, Felipe-Jiménez I, Suárez-Santana C, et al Morbillivirus infection in Risso’s dolphin *Grampus griseus*: a phylogenetic and pathological study of cases from the Canary Islands. Dis Aquat Organ. 2018; D3248. 10.3354/dao03248 30154276

[47] Higgins RJ, Krakowka SG, Metzler AE, Koestner A. Primary demyelination in experimental canine distemper virus induced encephalomyelitis in gnotobiotic dogs. Sequential immunologic and morphologic findings. Acta Neuropathol. 1982; 58 (1): 1–8. 7136512 10.1007/BF00692691 PMC7086558

[48] Blakemore WF, Summers BA, Appel MG. Evidence of oligodendrocyte infection and degeneration in canine distemper encephalomyelitis. Acta Neuropathol. 1989; 77 (5): 550–553. 2718748 10.1007/BF00687258

[49] Summers BA, Appel MJG. Demyelination in canine distemper encephalitis: an ultrastructural analysis. J Neurocytol. 1987; 16 (6): 871–881. 3450794 10.1007/BF01611991 PMC7089302

[50] Enders JF, Mc C.K., Mitus A, Cheatham WJ. Isolation of measles virus at autopsy in cases of giant-cell pneumonia without rash. N Engl J Med. 1959; 261: 875–881. 10.1056/NEJM195910292611801 13820247

[51] Lecis R, Tocchetti M, Rotta A, Naitana S, Ganges L, Pittau M, et al First gammaherpesvirus detection in a free-living Mediterranean bottlenose dolphin. J Zoo Wildl Med. 2014; 45 (4): 922–925. 10.1638/2014-0019.1 25632684

[52] Guarner J., Brandt ME. Histopathologic diagnosis of fungal infections in the 21st century. Clin Microbiol Rev. 2011; 24: 247–280. 10.1128/CMR.00053-10 21482725 PMC3122495

[53] Ioachim HL, Medeiros LJ. Measles lymphadenitis In: Ioachim HL, Medeiros LJ, editors. Ioachim's lymph node pathology: Lippincott Williams & Wilkins; 2009 pp. 97–99.

[54] McChesney MB, Miller CJ, Rota PA, Antipa L, Lerche NW, Ahmed R, et al Experimental measles. I. Pathogenesis in the normal and the immunized host. Virology 1997; 233 (1): 74–84. 10.1006/viro.1997.8576 9201218

[55] McChesney MB, Oldstone MB. Virus-induced immunosuppression: infections with measles virus and human immunodeficiency virus. Advances in immunology; 1989 pp. 335–380. 2665441 10.1016/s0065-2776(08)60696-3

[56] Iwatsuki K, Okita M, Ochikubo F, Gemma T, Shin YS, Miyashita N, et al Immunohistochemical analysis of the lymphoid organs of dogs naturally infected with canine distemper virus. J Comp Pathol. 1995; 113 (2): 185–190. 8543675 10.1016/s0021-9975(05)80033-7

[57] Nii S, Kamahora J, Mori Y, Takahashi M, Nishimura S, Okuno Y. Experimental pathology of measles in monkeys. Biken's Journal. 1963; 6 (4): 271–297. 14186824

[58] Garcia-Alvarez N, Martin V, Fernandez A, Almunia J, Xuriach A, Arbelo M, et al Levels and profiles of POPs (organochlorine pesticides, PCBs, and PAHs) in free-ranging common bottlenose dolphins of the Canary Islands, Spain. Sci Total Environ. 2014; 493: 22–31. 10.1016/j.scitotenv.2014.05.125 24937489

[59] Garcia-Alvarez N, Fernandez A, Boada LD, Zumbado M, Zaccaroni A, Arbelo M, et al Mercury and selenium status of bottlenose dolphins (*Tursiops truncatus*): A study in stranded animals on the Canary Islands. Sci Total Environ. 2015; 536: 489–498. 10.1016/j.scitotenv.2015.07.040 26232758

[60] Marsili L, D'Agostino A, Bucalossi D, Malatesta T, Fossi MC. Theoretical models to evaluate hazard due to organochlorine compounds (OCs) in Mediterranean striped dolphin (*Stenella coeruleoalba*). Chemosphere. 2004; 56 (8): 791–801. 10.1016/j.chemosphere.2004.03.014 15251294

