# Supplementary figures and images for: Comparative histopathologic and viral immunohistochemical studies on CeMV infection among Western Mediterranean, Northeast-Central, and Southwestern Atlantic cetaceans
Source: PLoS One. 2019 Mar 20;14(3):e0213363. doi: 10.1371/journal.pone.0213363 (PMC6426187; doi:10.1371/journal.pone.0213363)

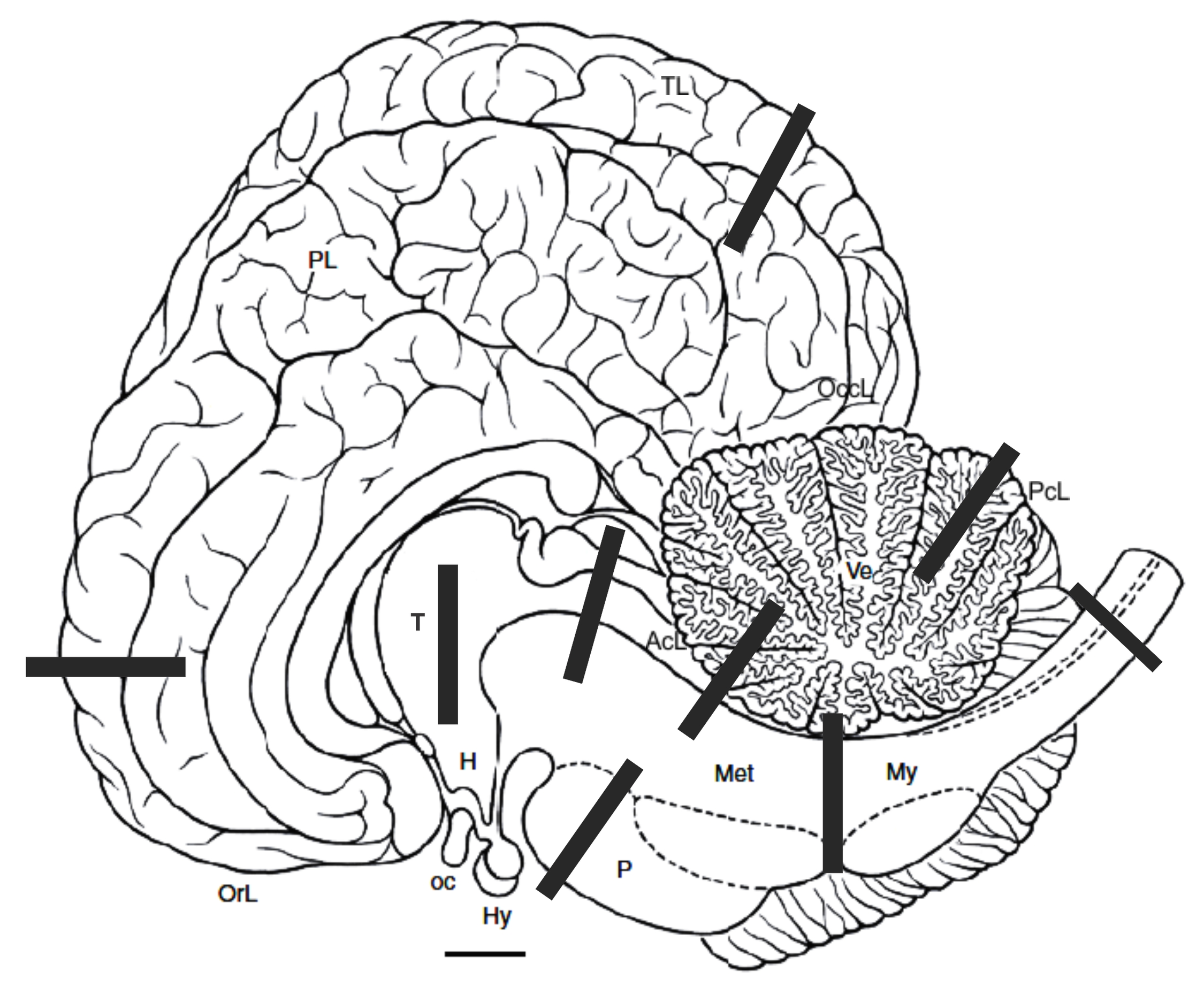

Supplement: S1 Fig — A) Areas for neuroanatomical sampling (indicated as black rectangles) in striped dolphins (Stenella coeruleoalba) and bottlenose dolphins from the Canary Islands (Spain). AcL, anterior cerebellar lobe; H, hypothalamus; Hy, hypophysis; Met, metencephalon; My, myelencephalon; OrL, orbital lobe; oc, optic chiasm; OccL, occipital lobe; PcL, posterior cerebellar lobe; PL, parietal lobe; T, thalamus; TL, temporal lobe; Ve, vermis. Brain diagram adapted from Oelschläger, H. & Oelschläger, J.S. (2009). (TIF) [file pone.0213363.s001.tif]
