# Supplementary material for: Comparative histopathologic and viral immunohistochemical studies on CeMV infection among Western Mediterranean, Northeast-Central, and Southwestern Atlantic cetaceans
Source: PLoS One. 2019 Mar 20;14(3):e0213363. doi: 10.1371/journal.pone.0213363 (PMC6426187; doi:10.1371/journal.pone.0213363)
Supplement: S1 Table — (DOCX) [file pone.0213363.s002.docx]

**S1 Table**. Template for recording histopathological findings in the central nervous system, including prosencephalon, mesencephalon and rhombencephalon.

| **Central Nervous System** | | | | | | |
| --- | --- | --- | --- | --- | --- | --- |
|  | | **Prosencephalon** | | **Mesencephalon** | **Rhombencephalon** | |
|  | | **Diencephalon** | **Telencephalon** |  | **Metencephalon** | **Myelencephalon** |
| ***Meningeal and neuroparenchymal vasculature*** | | | | | | |
| Congestion | |  |  |  |  |  |
| Endothelial hypertrophy | |  |  |  |  |  |
| Reactive neovascularization | |  |  |  |  |  |
| Perivascular swelling of astrocytic feet | |  |  |  |  |  |
| Perivascular hemorrhage | |  |  |  |  |  |
| Vasculitis/perivasculitis | |  |  |  |  |  |
| Perivascular cuffing | |  |  |  |  |  |
| Lymphocytes | |  |  |  |  |  |
| Lymphocytolysis | |  |  |  |  |  |
| Plasma cells | |  |  |  |  |  |
| Macrophages | |  |  |  |  |  |
| Neutrophils | |  |  |  |  |  |
| Eosinophils | |  |  |  |  |  |
| Basophils | |  |  |  |  |  |
| Mast cells | |  |  |  |  |  |
| Multinucleate giant cell/Syncytia | |  |  |  |  |  |
| Proliferation of adventitial cells or resident histiocytes | |  |  |  |  |  |
| Thrombosis | |  |  |  |  |  |
| Fibrosis | |  |  |  |  |  |
| Necrosis | |  |  |  |  |  |
| Fibrin | |  |  |  |  |  |
| Necrotizing vasculitis | |  |  |  |  |  |
| Leukocytosis | |  |  |  |  |  |
|  | |  |  |  |  |  |
| ***Meninges*** | | | | | | |
| Perivascular swelling of astrocytic feet | |  |  |  |  |  |
| Lymphocytes | |  |  |  |  |  |
| Plasma cells | |  |  |  |  |  |
| Macrophages | |  |  |  |  |  |
| Neutrophils | |  |  |  |  |  |
| Eosinophils | |  |  |  |  |  |
| Mott cell | |  |  |  |  |  |
| Hemorrhage | |  |  |  |  |  |
| Multinucleate giant cell/Syncytia | |  |  |  |  |  |
| Edema | |  |  |  |  |  |
| Fibrosis | |  |  |  |  |  |
| Necrosis | |  |  |  |  |  |
| Fibrin | |  |  |  |  |  |
| Bacteria | |  |  |  |  |  |
| Calcification | |  |  |  |  |  |
| ***Neuroparenchyma*** | | | | | | |
| **Neurons** | |  |  |  |  |  |
|  | Nuclear margination |  |  |  |  |  |
|  | Central chromatolysis |  |  |  |  |  |
|  | Peripheral chromatolysis |  |  |  |  |  |
|  | Neuronal atrophy |  |  |  |  |  |
|  | Acidophilic degeneration |  |  |  |  |  |
|  | Necrosis |  |  |  |  |  |
|  | Liquefactive necrosis |  |  |  |  |  |
|  | Necrosis with neuronophagia |  |  |  |  |  |
|  | Satellitosis |  |  |  |  |  |
|  | Spinal ganglion (satellitosis) |  |  |  |  |  |
|  | Neuronophagic nodules |  |  |  |  |  |
|  | Vacuolar degeneration |  |  |  |  |  |
|  | Pigments (ceroid, lipofuscin) |  |  |  |  |  |
|  | Neuromelanin |  |  |  |  |  |
|  | Siderotic pigmentation |  |  |  |  |  |
|  | Viral inclusion bodies |  |  |  |  |  |
|  | Non-viral inclusion bodies (Lafora, etc.) |  |  |  |  |  |
|  | Swollen axons (spheroids) |  |  |  |  |  |
|  | Axon loss |  |  |  |  |  |
|  | Digestion chamber |  |  |  |  |  |
|  | Wallerian degeneration |  |  |  |  |  |
| **Astrocytes** | |  |  |  |  |  |
|  | Perivascular astrocytic swelling |  |  |  |  |  |
|  | Glia limitans |  |  |  |  |  |
|  | Bergmann’s glia |  |  |  |  |  |
|  | Degeneration, necrosis, loss |  |  |  |  |  |
|  | Gemistocytes |  |  |  |  |  |
|  | Astrogliosis (proliferation) |  |  |  |  |  |
|  | Astrocytosis |  |  |  |  |  |
|  | Viral inclusion bodies |  |  |  |  |  |
|  | Alzheimer type II astrocytes |  |  |  |  |  |
|  | Phagocytosis |  |  |  |  |  |
|  | Rosenthal fibers |  |  |  |  |  |
| **Oligodendrocytes/Schwann cells** | |  |  |  |  |  |
|  | Hydropic change, swelling |  |  |  |  |  |
|  | Oligodendrogliosis |  |  |  |  |  |
|  | Necrosis |  |  |  |  |  |
|  | Loss |  |  |  |  |  |
|  | Mitosis |  |  |  |  |  |
|  | Interfascicular gliosis |  |  |  |  |  |
|  | Viral inclusion bodies |  |  |  |  |  |
|  | Schwann cell hyperplasia |  |  |  |  |  |
|  | Schwann cell phagocytosis |  |  |  |  |  |
|  | Bungner’s bands |  |  |  |  |  |
|  | Endoneurial fibrosis |  |  |  |  |  |
|  | Renaut bodies |  |  |  |  |  |
|  | Myelinophagy |  |  |  |  |  |
|  | Myelinic edema |  |  |  |  |  |
| **Ependymal cells** | |  |  |  |  |  |
|  | Atrophy |  |  |  |  |  |
|  | Tearing |  |  |  |  |  |
|  | Discontinuity |  |  |  |  |  |
|  | Ependymitis |  |  |  |  |  |
| **Choroid plexus** | |  |  |  |  |  |
|  | Atrophy |  |  |  |  |  |
|  | Tearing |  |  |  |  |  |
|  | Discontinuity |  |  |  |  |  |
|  | Inflammation |  |  |  |  |  |
|  | Fibrosis/hyalinization |  |  |  |  |  |
|  | Congestion, edema, hemorrhage |  |  |  |  |  |
|  | Extramedullary hematopoyesis |  |  |  |  |  |
| **Microglia** | |  |  |  |  |  |
|  | Reactive hypertrophy |  |  |  |  |  |
|  | Reactive hyperplasia (microgliosis - Rod cells) |  |  |  |  |  |
|  | Microglial nodules |  |  |  |  |  |
|  | Phagocytosis - Gitter cells |  |  |  |  |  |
|  | Perivascular microgliosis with pigment (ceroid/lipofuscin) |  |  |  |  |  |
|  | Neuronophagia |  |  |  |  |  |
| **Interstitium** | |  |  |  |  |  |
|  | Multinucleate giant cell/Syncytia |  |  |  |  |  |
|  | Myelinic edema (spongiosis) |  |  |  |  |  |
|  | Hemorrhage |  |  |  |  |  |
|  | Cavitation/rarefaction/necrosis |  |  |  |  |  |
|  | Intravascular/parenchymal hyphae |  |  |  |  |  |
|  | Spinal ganglion (satellitosis) |  |  |  |  |  |
