# Supplementary material for: Comparative histopathologic and viral immunohistochemical studies on CeMV infection among Western Mediterranean, Northeast-Central, and Southwestern Atlantic cetaceans
Source: PLoS One. 2019 Mar 20;14(3):e0213363. doi: 10.1371/journal.pone.0213363 (PMC6426187; doi:10.1371/journal.pone.0213363)
Supplement: S2 Table — (DOCX) [file pone.0213363.s003.docx]

**S2 Table**. Template for recording histopathological findings in the respiratory system

| **Distal respiratory system** | | | | | | |
| --- | --- | --- | --- | --- | --- | --- |
|  | **Trachea** | **Bronchi** | **Bronchioles** | **Alveoli** | **Interstitium** | **Pleura** |
| ***Vasculature*** | | | | | | |
| Congestion |  |  |  |  |  |  |
| Angiomatosis |  |  |  |  |  |  |
| Endothelial hypertrophy |  |  |  |  |  |  |
| Tunica media hypertrophy/hyperplasia |  |  |  |  |  |  |
| Vasculitis/perivasculitis |  |  |  |  |  |  |
| Thrombosis |  |  |  |  |  |  |
| Perivascular edema |  |  |  |  |  |  |
| Perivascular hemorrhage |  |  |  |  |  |  |
| Arteriosclerosis |  |  |  |  |  |  |
| Endarteritis | | | | | | |
| Hemosiderosis | | | | | | |
|  | | | | | | |
| **Mucosa/Epithelium** | | | | | | |
| Degeneration |  |  |  |  |  |  |
| Necrosis |  |  |  |  |  |  |
| Sloughing/loss |  |  |  |  |  |  |
| Type II pneumocyte hyperplasia |  |  |  |  |  |  |
| MGCS |  |  |  |  |  |  |
| Metaplasia |  |  |  |  |  |  |
| INCIBs |  |  |  |  |  |  |
|  | | | | | | |
| **Submucosa** | | | | | | |
| Mineralization |  |  |  |  |  |  |
| Edema |  |  |  |  |  |  |
| Fibrin |  |  |  |  |  |  |
| Hemorrhage |  |  |  |  |  |  |
| Neutrophils |  |  |  |  |  |  |
| Eosinophils |  |  |  |  |  |  |
| Macrophages |  |  |  |  |  |  |
| Lymphocytes |  |  |  |  |  |  |
| Plasma cells |  |  |  |  |  |  |
| Hemorrhage |  |  |  |  |  |  |
| Fibrosis |  |  |  |  |  |  |
| Chondronecrosis/-lysis |  |  |  |  |  |  |
| Fibromuscular hyperplasia |  |  |  |  |  |  |
|  | | | | | | |
| **Lumen** | | | | | | |
| Edema |  |  |  |  |  |  |
| Fibrin |  |  |  |  |  |  |
| Hemorrhage |  |  |  |  |  |  |
| Neutrophils |  |  |  |  |  |  |
| Eosinophils |  |  |  |  |  |  |
| Macrophages |  |  |  |  |  |  |
| Lymphocytes |  |  |  |  |  |  |
| Plasma cells |  |  |  |  |  |  |
| Keratin squames/meconium |  |  |  |  |  |  |
| Multinucleate giant cell/Syncytia |  |  |  |  |  |  |
| Necrotic cell debris |  |  |  |  |  |  |
| Bacteria |  |  |  |  |  |  |
| Parasites |  |  |  |  |  |  |
| Fungi |  |  |  |  |  |  |
| Emphysema | | | | | | |
| Abscess | | | | | | |
| Bronchiectasis | | | | | | |
| Ceroid-like lipid globules | | | | | | |
| Aspirated particles | | | | | | |
| Mucus | | | | | | |
| Cholesterol clefts | | | | | | |
| Hemosiderophages | | | | | | |
| Bronchoconstriction | | | | | | |
| Atelectasia | | | | | | |
| Large calcifications | | | | | | |
|  | | | | | | |
| **Interstitium** | | | | | | |
| Edema |  |  |  |  |  |  |
| Fibrin |  |  |  |  |  |  |
| Hemorrhage |  |  |  |  |  |  |
| Neutrophils |  |  |  |  |  |  |
| Eosinophils |  |  |  |  |  |  |
| Macrophages |  |  |  |  |  |  |
| Lymphocytes |  |  |  |  |  |  |
| Plasma cells |  |  |  |  |  |  |
| Necrotic cell debris |  |  |  |  |  |  |
| Hemosiderosis |  |  |  |  |  |  |
| Fibrosis |  |  |  |  |  |  |
| Severe remodeling |  |  |  |  |  |  |
| Sclerotic granulomas |  |  |  |  |  |  |
| Calcifications |  |  |  |  |  |  |
|  | | | | | | |
| **Pleura** | | | | | | |
| Inflammation |  |  |  |  |  |  |
| Fibrosis |  |  |  |  |  |  |
|  |  |  |  |  |  |  |
