# Supplementary material for: Comparative histopathologic and viral immunohistochemical studies on CeMV infection among Western Mediterranean, Northeast-Central, and Southwestern Atlantic cetaceans
Source: PLoS One. 2019 Mar 20;14(3):e0213363. doi: 10.1371/journal.pone.0213363 (PMC6426187; doi:10.1371/journal.pone.0213363)
Supplement: S3 Table — (DOCX) [file pone.0213363.s004.docx]

**S3 Table.** Template for recording histopathological findings in the lymphoid system (lymph nodes, spleen)

|  | | **Lymph node** | | | | | | **Spleen** | | | | | | |  |
| --- | --- | --- | --- | --- | --- | --- | --- | --- | --- | --- | --- | --- | --- | --- | --- |
|  |  | **Follicles** | | | **Paracortex** | **Medullary cords** | **Sinuses** | | **White pulp** | | | | **Red pulp** | | |
|  |  | Primary | Secondary | |  |  |  |  | Follicle | | | PALS | Sinuses | Cords | |
|  |  |  | GC | MZ |  |  |  |  | GC | MZ | MGZ |  |  |  |  |
| **Congestion** | |  |  |  |  |  |  | |  |  |  |  |  |  | |
| **Reactive hyperplasia** | |  |  |  |  |  |  | |  |  |  |  |  |  | |
| **Depletion** | |  |  |  |  |  |  | |  |  |  |  |  |  | |
| **Lymphocytolysis** | |  |  |  |  |  |  | |  |  |  |  |  |  | |
| **Necrosis** | |  |  |  |  |  |  | |  |  |  |  |  |  | |
| **Edema** | |  |  |  |  |  |  | |  |  |  |  |  |  | |
| **Fibrin** | |  |  |  |  |  |  | |  |  |  |  |  |  | |
| **Erythrocytosis** | |  |  |  |  |  |  | |  |  |  |  |  |  | |
| **Erythrophagocytosis** | |  |  |  |  |  |  | |  |  |  |  |  |  | |
| **Leukocytosis** | |  |  |  |  |  |  | |  |  |  |  |  |  | |
| **Leukophagocytosis** | |  |  |  |  |  |  | |  |  |  |  |  |  | |
| **Histiocytosis** | |  |  |  |  |  |  | |  |  |  |  |  |  | |
| **Hemosiderosis** | |  |  |  |  |  |  | |  |  |  |  |  |  | |
| **Multinucleate giant cells/Syncytia** | |  |  |  |  |  |  | |  |  |  |  |  |  | |
| **Inflammation** | |  |  |  |  |  |  | |  |  |  |  |  |  | |
|  | Lymphocytes |  |  |  |  |  |  | |  |  |  |  |  |  | |
|  | Plasma cells |  |  |  |  |  |  | |  |  |  |  |  |  | |
|  | Mott cells |  |  |  |  |  |  | |  |  |  |  |  |  | |
|  | Macrophages |  |  |  |  |  |  | |  |  |  |  |  |  | |
|  | Neutrophils |  |  |  |  |  |  | |  |  |  |  |  |  | |
|  | Eosinophils |  |  |  |  |  |  | |  |  |  |  |  |  | |
|  | Basophils |  |  |  |  |  |  | |  |  |  |  |  |  | |
|  | Mast cells |  |  |  |  |  |  | |  |  |  |  |  |  | |
| **Fibrosis** | |  |  |  |  |  |  | |  |  |  |  |  |  | |
| **Hyalinosis** | |  |  |  |  |  |  | |  |  |  |  |  |  | |
| **Amyloid** | |  |  |  |  |  |  | |  |  |  |  |  |  | |
| **Mineralization** | |  |  |  |  |  |  | |  |  |  |  |  |  | |
| **Gas/Fat bubbles** | |  |  |  |  |  |  | |  |  |  |  |  |  | |
| **Hematopoyesis** | |  |  |  |  |  |  | |  |  |  |  |  |  | |
| **Capsular hemorrhage** | |  |  |  |  |  |  | |  |  |  |  |  |  | |
| **Siderocalcinosis** | |  |  |  |  |  |  | |  |  |  |  |  |  | |
| **INCIBs** | |  |  |  |  |  |  | |  |  |  |  |  |  | |
| Vascularization/angiomatosis | |  |  |  |  |  |  | |  |  |  |  |  |  | |
| Fibrinoid vascular necrosis | |  |  |  |  |  |  | |  |  |  |  |  |  | |
| Vascularization/angiomatosis | |  |  |  |  |  |  | |  |  |  |  |  |  | |
| Ceroid (yellow pigment) | |  |  |  |  |  |  | |  |  |  |  |  |  | |
| Melanosis/melanomacrophages | |  |  |  |  |  |  | |  |  |  |  |  |  | |

GC, germinal center; MZ, mantle zone; MGZ, marginal zone; PALS, periarteriolar lymphoid sheath.
