# Supplementary material for: Comparative histopathologic and viral immunohistochemical studies on CeMV infection among Western Mediterranean, Northeast-Central, and Southwestern Atlantic cetaceans
Source: PLoS One. 2019 Mar 20;14(3):e0213363. doi: 10.1371/journal.pone.0213363 (PMC6426187; doi:10.1371/journal.pone.0213363)
Supplement: S4 Table — (DOCX) [file pone.0213363.s005.docx]

**S4 Table.** Gross and microscopic pathologic findings, and most probable cause(s) of stranding and/or death (COD) in Guiana dolphins (*Sotalia guianensis*), striped dolphins (*Stenella coeruleoalba*) and bottlenose dolphins (*Tursiops truncatus*) included in this study. PASLNS: pathology associated with significant loss of nutritional status. PAGNS: Pathology associated with good nutritional status.

| **No** | **Gross** | **Microscopic** | **COD** |
| --- | --- | --- | --- |
| 1 | (Lactating) Diffuse axial skeletal muscle atrophy; Proliferative pleuritis; Pulmonary edema; Hemorrhagic enteritis; Hepatomegaly and congestion. | **Lung**: Marked, multifocal, chronic bronchointerstitial pneumonia and proliferative pleuritis with sclerosis, type II pneumocyte hyperplasia, MGCS, rare INCIBs, calcified nematode debris and edema; MATMHH.  **Mammary gland**: Mid, multifocal, chronic lymphoplasmacytic and histiocytic mastitis with acinar ectasia, inspissated secretion, scattered necrosis, ceroid pigment and moderate epithelial INCIBs.  **Heart:** Mild, focal, subacute fibrinous pericarditis; Mild, multifocal myocardial fibrosis.  **Kidney**: Mild, multifocal, chronic membranous glomerulonephritis with glomerulocysts, tubular proteinosis, protein casts, and scattered tubuloepithelial necrosis.  **Pulmonary lymph node**: Mild, multifocal, chronic eosinophilic lymphadenitis with necrosis, fibrosis and hemosiderosis; Diffuse lymphoid depletion.  **Mediastinal lymph node:** Mild, multifocal, chronic eosinophilic lymphadenitis.  **Spleen**: Diffuse congestion and multifocal, acute capsular hemorrhage; Extramedullary hematopoiesis.  **Adrenal**: Mild, multifocal, acute corticomedullary hemorrhage.  **Aorta**: Mild, segmental, chronic proliferative endarteritis.  **Liver**: Moderate, multifocal, chronic bile duct adenomatous hyperplasia.  **Uterus**: Moderate, multifocal, chronic arteriosclerosis and arterial elastosis.  **Glandular stomach:** Mild, diffuse mucosal hyperplasia; MATMHH.  **Large intestine, Thyroid, Skin, Trachea, Cerebrum, Cerebellum, Spinal cord, Skeletal muscle:** NSLO. | Acute systemic CeMV infection |
| 2 | Pulmonary edema; Hydropericardium; Hepatic lipidosis; Gastric ulcers. | **Lung**: Mild, multifocal, acute interstitial pneumonia with marked alveolar edema, hemorrhage and histiocytosis, MGCS and keratin spicules.  **Kidney**: Mild, multifocal, acute tubular degeneration and necrosis; Mild, multifocal, acute tubular proteinosis and protein casts; Marked, focal, acute perirrenal hemorrhage.  **Pulmonary lymph node:** Moderate, diffuse cortical and paracortical lymphoid depletion with lymphocytolysis.  **Prescapular lymph node**: Diffuse congestion with focal acute hemorrhage and erythrophagocytosis; Sinus vascularization.  **Spleen**: Mild, multifocal lymphoid depletion with sinus histiocytosis; Extramedullary hematopoiesis.  **Heart:** Moderate, multifocal, acute subendocardial and epicardial hemorrhage; Focal mural vasculitis.  **Adrenal**: Marked, multifocal, acute corticomedullary hemorrhagic necrosis.  **Esophagus:** Focal acute hemorrhage in serosa.  **Urinary bladder**: Edema and focal acute hemorrhage in serosa.  **Penis**: Urethral luminal hemorrhage with single cell epithelial necrosis/apoptosis.  **Cerebrum, cerebellum:** Diffuse leptomeningeal congestion and perivascular edema in neuroparenchyma.  **Keratinized and Pyloric stomach, Tongue, Aorta, Small intestine, Pancreas, Trachea:** NSLO. | Acute systemic CeMV infection |
| 3 | Focal mandibular subcutaneous hematoma; Lack of ingesta. | **Lung**: Severe, disseminated, subacute to chronic fibrinonecrosuppurative bronchointerstitial pneumonia with chrondronecrosis, chondrolysis, bronchial sclerosis, numerous hyphae, larval and adult nematodes, mixed bacteria, type II pneumocyte hyperplasia with MGCS, vasculitis, thrombosis and ischemic necrosis, hemorrhage, edema and fibrosis; Multifocal endarteritis and MATMHH.  **Cerebrum**: Marked, multifocal to coalescing, acute hemorrhagic necrosis of neuroparenchyma with multifocal obliterative thromboemboli (infarction).  **Kidney**: Mild, focal, acute thromboembolic nephritis with intravascular fungal hyphae; Moderate, multifocal, chronic lymphoplasmacytic and histiocytic interstitial nephritis with fibrosis and intralesional nematodes; Moderate, multifocal, chronic membranous glomerulonephritis with Bowman’s capsule fibrosis, glomerular atrophy, loss and glomerulocysts and tubular proteinosis and protein casts; Multifocal acute tubular degeneration and necrosis; Marked medullary tubular mineralization.  **Mediastinal lymph node**: Moderate, diffuse cortical and paracortical lymphoid depletion with diffuse sinus histiocytosis, hemosiderosis, edema and scattered MGCS; Mild to moderate, multifocal, chronic (nodular) eosinophilic lymphadenitis with necrosis.  **Spleen**: Diffuse sinus histiocytosis and mild lymphoid depletion; Extramedullary hematopoiesis.  **Liver**: Mild, multifocal, chronic lymphoplasmacytic and histiocytic pericholangitis with mild fibrosis, mild bile duct hyperplasia and sinusoidal leukocytosis; Scattered portal endarteritis and MATMHH.  **Skin**: Minimal, multifocal epidermal hyperplasia with rare intracytoplasmic inclusions (suggestive of *Cetacean Poxvirus*).  **Thyroid**: Congestion and focal fibrinocellular thrombus.  **Bladder**: Moderate, multifocal, chronic mural fibrosis with myocyte atrophy and loss and minimal fibrosis.  **Skeletal muscle**: Rare hypercontracted myofibers.  **Glandular stomach**: Mild, diffuse mucosal hyperplasia with focal intracrypt degenerated nematode larvae.  **Adrenal**: Focal granuloma.  **Pancreas**: Focal pleocellular periductitis.  **Cerebellum, Spinal cord, Testicle, Small intestine, Heart, Keratinized stomach, Ovary**: NSLO. | Subacute systemic CeMV infection and multisystemic mycosis |
| 4 | Multifocal ulcerated cutaneous infarcts; Ascites; Pulmonary edema; Fibrinosuppurative vulvitis; Acute epidural hematoma; Lack of ingesta. | **Lung**: Marked, acute, diffuse alveolar edema with hemorrhage and acute interstitial pneumonia with MGCS, occasional keratin squames and aspirated material.  **Mediastinal, Mesenteric lymph node**: Moderate to marked, diffuse lymphoid depletion with sinus histiocytosis and MGCS.  **Skin**: Marked, focally extensive suppurative to pyogranulomatous dermatitis with focal deep dermal necrotizing vasculitis, thrombosis and necrosis (dermocutaneous infarction); Multifocal epidermal hyperplasia with intra- intercellular edema and peripheral INIBs.  **Thymus**: Mild lymphoid depletion.  **Kidney**: Marked, multifocal, acute perirrenal edema and hemorrhage.  **Colon**: Mild, multifocal, subacute neutrophilic colitis with cryptitis/crypt abscesses and GALT lymphoid depletion.  **Tongue**: Scattered hydropic change in mucosal epithelium.  **Cerebrum**: Mild, multifocal leptomeningeal perivascular edema and astrocytosis.  **Eye**: Minimal, focal, acute neutrophilic choroiditis and neutrophilia.  **Bladder, Penis, Ovary, Trachea, Pancreas, Heart, Keratinized and Glandular stomach, Aorta, Small intestine, Uterus, Skeletal muscle, Cerebellum, Palate, Brainstem, Spinal cord, Heart**: NSLO. | Acute systemic CeMV infection |
| 5 | Ascites; Verminous pleuropneumonia; Pulmonary edema; Left ventricle myocardial hypertrophy; Acute hepatic passive congestion; Multifocal intestinal hemorrhage; Multifocal ulcerative gastritis; Multifocal, chronic interstitial nephritis; Abdominal lymphadenomegaly; Lack of ingesta. | **Lung**: Mild, multifocal, chronic bronchointerstitial pneumonia with type II pneumocytes, scattered MGCS, nematodes, calcification and fibrosis.  **Mediastinal, Mesenteric lymph nodes**: Diffuse lymphoid depletion, lymphocytolysis with MGCS, sinus erythrocytosis and erythrophagocytosis.  **Liver**: Mild to moderate, multifocal, acute centrilobular and paracentral hepatocyte degeneration, necrosis, loss and hemorrhage; Mild, diffuse hepatocellular atrophy and microvacuolar change; Minimal, focal subcapsular lymphocytic hepatitis.  **Cerebrum**: Multifocal, acute hemorrhage and perivascular edema.  **Trachea**: Minimal, focal, lymphocytic tracheitis.  **Skeletal muscle**: Multifocal, acute myocyte degeneration and necrosis.  **Kidney**: Mild, multifocal, acute tubular degeneration and necrosis.  **Bladder**: Multifocal ICIBs.  **Spleen**: Multifocal, acute capsular hemorrhage; Mild lymphoid (follicular) depletion.  **Heart, Small intestine, Skeletal muscle, Tongue, Cerebellum, Testicle, Skin, Aorta, Palate**: NSLO. | Acute systemic CeMV infection |
| 6 | Hemoperitoneum; Icterus; Multifocal epaxial myopathy; Focal bilateral subscapular hemorrhage; Cephalic linear traumatic wounding (anthropogenic cut); Lack of ingesta. | **Liver**: Marked, diffuse hepatic lipidosis with necrosis and loss; Mild, multifocal, chronic lymphocytic periportal hepatitis.  **Cerebrum, Brainstem**: Mild leptomeningeal and neuroparenchymal perivascular edema.  **Lymph node**: Mild, diffuse lymphoid depletion and mild sinus histiocytosis.  **Lung**: Minimal, multifocal, acute suppurative bronchopneumonia with alveolar histiocytosis, scattered MGCS and edema; Mild, multifocal atelectasia; Moderate, multifocal bronchial/bronchiolar calcifications.  **Kidney**: Mild, diffuse tubuloepithelial vacuolar change; Marked perirrenal edema.  **Skeletal muscle**: Multifocal acute myocyte degeneration and necrosis.  **Skin**: Moderate, diffuse, chronic regular to irregular epidermal hyperplasia with intra- and extracellular edema and scattered ICIBs (compatible with *CetaceanPoxvirus*).  **Tongue, Heart, Adrenal, Cerebellum, Spinal cord, Small intestine, Bladder, Glandular stomach, Tongue, Prostate, Colon**: NSLO. | Acute systemic CeMV infection |
| 7 | (Pregnant) Proliferative fibrosing (villous) pleuritis; Focal bilateral subscapular hemorrhage. | **Lung**: Moderate to marked, multifocal, chronic bronchointerstitial pneumonia with type II pneumocyte hyperplasia, scattered nematode larvae and marked fibrosis; Marked MATMHH.  **Kidney**: Moderate to marked, multifocal to coalescing, chronic interstitial nephritis with fibrosis, Bowman’s capsule fibrosis, glomerulocysts, glomerular atrophy and loss, tubular proteinosis and protein casts; Multifocal acute tubuloepithelial necrosis; Tubular medullary mineralization.  **Skeletal muscle**: Marked, focally extensive, acute perimuscular/fascial hemorrhage and scattered myocyte degeneration and necrosis.  **Heart**: Mild, focal, chronic epicarditis.  **Skin**: Moderate, multifocal irregular epidermal hyperplasia with ICIBs and hydropic change (compatible with *CetaceanPoxvirus*).  **Cerebrum**: Mild, multifocal, acute perivascular edema with astrocytic ballooning.  **Liver**: Mild to moderate, multifocal hemosiderosis.  **Adrenal, Esophagus, Skeletal muscle, Cerebellum, Spinal cord**: NSLO | Acute systemic CeMV infection |
| 8 | Proliferative fibrosing (villous) pleuritis; Hepatic lipidosis; Congestive splenomegaly; Lack of ingesta. | **Lung**: Marked, multifocal, chronic bronchointerstitial pneumonia with marked alveolar histiocytosis, MGCS, type II pneumocyte hyperplasia, nematodes, interstitial and pleural fibrosis; MATMHH.  **Mediastinal lymph node**: Marked, diffuse, chronic lymphoid depletion with fibrosis, sinus histiocytosis, hemosiderosis and rare MGCS; Mild to moderate, multifocal, chronic (nodular) eosinophilic lymphadenitis with necrosis.  **Mesenteric lymph node:** Cortical lymphoid depletion with hyalinosis, sinus histiocytosis and hemosiderosis.  **Kidney**: Mild, multifocal, chronic interstitial nephritis with fibrosis, Bowman’s capsule fibrosis, glomerular atrophy, glomerulocysts and scattered tubular proteinosis; Scattered acute hemorrhage.  **Brainstem**: Mild, multifocal leptomeningeal and neuroparenchymal perivascular edema with astrocytosis.  **Cerebrum**: Scattered leptomeningeal acute hemorrhage; Mild, multifocal leukocytosis.  **Cerebellum**: Scattered white matter spongiosis, perivascular edema and astrocytosis.  **Heart**: Scattered acute hemorrhage; Mild, multifocal neutrophilia.  **Spleen**: Moderate to marked, diffuse hemosiderosis and sinus histiocytosis; Mild, diffuse lymphoid depletion.  **Liver**: Mild to moderate multifocal cholestasis.  **Diaphragm**: Scattered hyaline myodegeneration.  **Small intestine, Skeletal muscle, Keratinized and Glandular stomach, Bladder, Spinal cord, Adrenal**: NSLO. | Acute systemic CeMV infection |
| 9 | Ascites; Focal, bilateral subscapular hemorrhage; Bilateral pulmonary emphysema with left focal rupture and hemorrhage; Suppurative bronchopneumonia; Fibrous pleuritis; Focal cardiac mural apical hemorrhage; Hydropericardium; Multifocal hemorrhagic gastritis; Lack of ingesta. | **Lung**: Moderate to marked, diffuse, chronic suppurative bronchointerstitial pneumonia with interstitial and pleural fibrosis and severe, locally extensive, acute hemorrhage, thrombosis and necrosis and rupture, calcifications, nematodes and bacteria; Marked alveolar histiocytosis with cholesterol clefts and granulomas; Angiomatosis, chronic endarteritis and MATMHH.  **Cerebrum**: Severe, multifocal, acute leptomeningeal hemorrhage, perivascular edema and acute neuronal necrosis; Focal lymphocytic leptomeningitis and mild gliosis.  **Kidney**: Mild to moderate, multifocal, chronic interstitial nephritis with glomerular atrophy, loss and glomerulocysts.  **Mediastinal, Mesenteric lymph nodes**: Mild to moderate, multifocal, subacute to chronic (nodular) eosinophilic lymphadenitis with necrosis; Diffuse lymphoid depletion, sinus histiocytosis, hemosiderosis and scattered MGCS.  **Spleen**: Moderate, diffuse sinus histiocytosis and hemosiderosis.  **Adrenal, Glandular stomach, Thyroid, Aorta, Small intestine, Colon, Testicle**: NSLO. | Acute systemic CeMV infection |
| 10 | Marked, multifocal ulcerative glossitis, palatitis and oropharyngitis; Multifocal rostrum skin erosions; Focal proliferative skin lesion; Mandibular lateral deviation; Generalized icterus; Focal, chronic (healed) rib fracture; Parasitic pleuropneumonia; Marked pyloric *Braunina cordiformis* parasitosis with scattered hemorrhage. | **Lung**: Marked, multifocal to coalescing, chronic bronchointerstitial pneumonia with type II pneumocyte hyperplasia, INCIBs, alveolar histiocytosis, nematodes, marked sclerosing interstitial and pleural fibrosis; Angiomatosis and MATMHH.  **Cerebrum**: Focal neuroparenchymal hemorrhage and rarefaction; Minimal, focal lymphocytic meningitis and mild neutrophilia; Mild, multifocal, perivascular edema.  **Eye**: Rare ICIBs in astrocytes/neurons of retina.  **Large intestine**: Marked, multifocal lymphoid depletion of GALT.  **Mesenteric lymph node**: Diffuse lymphoid depletion; Mild, multifocal, chronic eosinophilic lymphadenitis with necrosis; Moderate, diffuse hemosiderosis.  **Bladder**: Mild, multifocal, chronic superficial lymphocytic cystitis with INCIBs.  **Mediastinal lymph node**: Marked, multifocal cortical depletion with lymphocytolysis and paracortical hyperplasia, sinus histiocytosis, scattered MGCS and hemosiderosis; Extramedullary hematopoiesis.  **Glandular stomach**: Mild, focal, chronic lymphocytic serositis.  **Palate/oropharynx**: Marked, multifocal salivary gland ectasia with inspissated calcified secretion and mild syaloadenitis; Multifocal, subacute ulcers.  **Penis**: Mild, multifocal regular and irregular mucosal hyperplasia; Multifocal ICIBs in penile urethra.  **Tongue**: Marked, focal, subacute ulcer with mild interstitial glossitis and scattered myocyte degeneration and necrosis.  **Kidney**: Mild, multifocal, chronic lymphoplasmacytic interstitial nephritis and membranous glomerulopathy with Bowman’s capsule fibrosis, glomerular atrophy and loss, glomerulocysts and mild tubular proteinosis; Tubular medullary mineralization.  **Liver**: Mild, multifocal, acute centrilobular hepatocellular degeneration and loss with vacuolar change and multifocal cholestasis.  **Spleen**: Marked, diffuse sinus histiocytosis, hemosiderosis and scattered MGCS; Mild, multifocal eosinophilic splenitis.  **Skin lesions**: Moderate to marked, focally extensive, chronic irregular proliferative dermatitis with intracellular edema, ICIBs (compatible with *CetaceanPoxvirus*) and dermoepidermal clefting and scattered keratin pearls.  **Heart**: Mild, multifocal neutrophilia.  **Spinal cord**: Mild, multifocal neutrophilia.  **Adrenal, Testicle, Epididymis, Pancreas, Prostate, Diaphragm, Trachea, Aorta, Small and Large intestine, Cerebellum, Small intestine**: NSLO. | Acute systemic CeMV infection |
| 11 | Diffuse cyanotic mucous membranes; Hemothorax; Generalized icterus; Parasitic hemorrhagic pneumonia; Suppurative bronchopneumonia; Pulmonary edema; Severe hepatic lipidosis and passive congestion; Congestive splenomegaly; Brain congestion with focal epidural hematoma; Lack of ingesta. | **Liver**: Marked, multifocal random to massive, acute necrotizing hepatitis with hemorrhage, thrombosis and cholestasis; Mild, multifocal, chronic lymphocytic pericholangitis.  **Lung**: Marked, multifocal, acute suppurative to pyogranulomatous and necrotizing bronchopneumonia with occlusive bronchitis, myriads of Zygomycete-like hyphae, adult and larval nematodes, type II pneumocyte hyperplasia, MGCS with INCIBs, bacteria, edema and hemorrhage; Moderate, multifocal alveolar, bronchiolar and bronchial mineralization.  **Kidney**: Mild, multifocal, acute tubular necrosis with rare INCIBs, hemoglobin casts, tubular proteinosis and cellular casts; Scattered glomerulocysts and rare interstitial fibrosis.  **Spleen**: Marked, focally extensive, acute hemorrhagic necrosis, sinus histiocytosis and hemosiderosis.  **Prescapular, Mediastinal lymph node**: Diffuse lymphoid depletion with sinus histiocytosis, hemosiderosis, leukocytosis, mild fibrin and edema; Mild, diffuse medullary cord lymphoid hyperplasia and plasmacytosis.  **Mesenteric lymph node**: Marked, diffuse lymphoid depletion with histiocytosis, hemosiderosis and fibrosis; Mild, multifocal, subacute eosinophilic nodular lymphadenitis with necrosis.  **Cerebrum, cerebellum**: Multifocal, acute meningeal congestion with scattered acute hemorrhage.  **Tongue**: Scattered ICIBs in mucosal epithelium.  **Skin**: Focal epidermal hyperplasia.  **Keratinized stomach**: Mild, multifocal mucosal hyperplasia with diffuse hyperkeratosis.  **Skeletal muscle**: Scattered, acute myocyte degeneration.  **Heart, Thyroid, Aorta, Colon, Glandular stomach, Urinary bladder, Spinal cord, Adrenal gland**: NSLO. | Subacute systemic CeMV infection |
| 12 | Numerous intra-/interspecific scarred markings; Live stranding-related cutaneous erosions; Multifocal maxillary and mandibular trauma with hemorrhage and soft tissue loss; Focal proliferative/verrucous lesion in caudal peduncle; Severe parasitization by *Phyllobotrium delphini* plerocercoids in subcutis of caudoventral abdomen; Marked bilateral suprascapular edema; Multifocal nodular granulomatous gastritis by *Pholeter gastrophilus* (glandular stomach); Severe peritoneal parasitization by *Monorygma grimaldi* plerocercoids; Moderate, diffuse pleural lymphangiectasia; bilateral diffuse pulmonary atelectasia; Generalized congestion. | **Cerebrum**, **Cerebellum**, **Medulla oblongata**, **Spinal cord**: Mild to moderate, multifocal, chronic lymphocytic meningoencephalitis with perivascular cuffing, astrocytosis/gliosis, microgliosis/rod cells, glial nodules, satellitosis, rare neuronophagia and MGCS.  **Skin**: Mild, multifocal, chronic epidermal regular to irregular hyperplasia with rare eosinophilic ICIBs (compatible with *CetaceanPoxvirus*); Focal, acute superficial dermal microthrombus; Mild, multifocal, acute deep dermal hemorrhage with microthrombi; Focal microgranuloma; Marked, focally extensive, subacute proliferative and pustular dermatitis with vasculitis/perivasculitis, thrombosis, numerous ICIBs (compatible with *CetaceanPoxvirus*), hyperkeratosis and necrosis.  **Skeletal muscle**: Scattered acute single cell segmental hyaline degeneration and necrosis.  **Lung**: Mild, multifocal, acute to subacute neutrophilic and histiocytic bronchointerstitial pneumonia with rare type II pneumocyte hyperplasia and MGCS, edema and hemorrhage; Mild, multifocal, chronic lymphocytic bronchitis with fibrosis; Diffuse congestion; Multifocal atelectasia; Moderate, multifocal mucosal mineralization; Scattered bronchoconstriction.  **Prescapular lymph node**: Mild, diffuse lymphoid hyperplasia with medullary cord plasmacytosis, sinus histiocytosis, erythrocytosis, erythrophagia, leukocytosis, leukophagia, rare MGCS, hemosiderosis.  **Liver**: Mild to moderate, multifocal, chronic lymphoplasmacytic pericholangitis with rare ICIBs (bile duct epithelium) to chronic reactive hepatitis and rare single cell hepatocellular degeneration; Mild, multifocal Ito cell and Kupffer hypertrophy/hyperplasia with vacuolated cytoplasm and presumed eosinophilic ICIBs and hemosiderosis; Mild bile duct hyperplasia.  **Pancreas**: Moderate, multifocal eosinophilic INIBs in endocrine epithelium.  **Keratinized stomach**: Mild, multifocal, chronic irregular mucosal hyperplasia.  **Glandular stomach**: Mild, multifocal lymphocytic gastritis with scattered neutrophilic cryptitis and rare microhemorrhage.  **Pyloric stomach**: Mild, multifocal, chronic lymphoplasmacytic gastritis.  **Small and large intestine**: Mild, multifocal, chronic lymphoplasmacytic and eosinophilic enterocolitis with apical fibrosis.  **Mesenteric lymph node**: Mild, diffuse lymphoid depletion; Moderate, multifocal, subacute eosinophilic lymphadenitis with sinus histiocytosis, marked sinus erythrocytosis with erythrophagia, hemosiderosis and leukocytosis.  **Kidney**: Mild, multifocal, chronic membranoproliferative glomerulopathy with Bowman’s capsule fibrosis, rare glomerulosclerosis, scattered tubular proteinosis and mineralization; Mild, multifocal, acute tubular degeneration; Diffuse congestion.  **Urinary bladder**: Focally extensive, acute serosal hemorrhage; Mild, focal lymphoplasmacytic perivascular serositis.  **Spleen**: Mild, multifocal lymphoid reactive hyperplasia with follicular depletion, hyalinosis and amyloidosis; Mild multifocal capsular and vascular siderocalcinosis, focally associated with granulomatous reaction; Diffuse congestion and multifocal acute capsular hemorrhage; Extramedullary hematopoiesis.  **Adrenal gland**: Multifocal nodular and diffuse cortical hyperplasia; Multifocal acute periadrenal hemorrhage; Multifocal myxoid degeneration.  **Subcutaneous fat**: Mild, diffuse edema and scattered acute hemorrhage.  **Hypophysis**: Scattered adenohypophyseal cysts.  **Thyroid**: Multifocal acute interstitial and peripheral hemorrhage.  **Testicle:** NSLO. | Chronic systemic CeMV infection |
| 13 | Mild epiobiosis by *Xenobalanus* sp. in the caudal fin; Numerous intra-/interspecific interaction marks; Live stranding-related cutaneous erosions and lacerations throughout the rostrum and ventral surface; Multifocal 1-2 cm in diameter, depigmented foci throughout the body; Mild parasitization by *P. delphini* plerocercoids in the anogenital subcutis; Bilateral purulent and proliferative scapulo-humeral osteoarthritis; Small amount of ingesta; Marked, focal ulcerative (5 cm in diameter) gastritis with numerous anchored *Anisakis* sp. nematodes in the keratinized stomach; Multifocal erosive and hemorrhagic gastritis (glandular stomach); Multifocal nodular granulomatous pyloric gastritis by *P. gastrophilus* trematodes; Mild parasitization by adult cestodes (Diphyllobothriidae) in the pyloric stomach and small intestine; Moderate parasitization by *M. grimaldi* plerocercoids throughout abdominal serosae; Moderate to severe parasitization by *Stenurus* sp. nematodes with scattered calcified granulomas; bilateral pulmonary edema; Generalized lymphadenomegaly; Bilateral adrenomegaly and congestion; Bilateral, mild to moderate pterygoid sinus parasitization by *Nasitrema* sp. and *Crassicauda grampicola*; Multifocal leptomeningeal fibrosis; Scattered gas bubbles in leptomeningeal vasculature. | **Skin**: Mild, multifocal irregular epidermal hyperplasia with mild intracellular edema.  **Skeletal muscle**: Scattered single cell myodegeneration and segmental hyaline necrosis with myoglobin globules and interstitial edema.  **Lung**: Marked, multifocal, acute suppurative and histiocytic bronchointerstitial pneumonia with bronchitis necrosis, fibrin, edema, hemorrhage, MGCS with INCIBs, epithelial necrosis, scattered type II pneumocyte hyperplasia, metaplasia, adult and larval *Stenurus* sp. nematodes; Marked, focal, acute vasculitis with fibrinocellular thrombosis and bacteria; Multifocal atelectasia; Mild, multifocal mineralizations; Extramedullary hematopoiesis.  **Mediastinal lymph node**: Moderate, multifocal, chronic histiocytic lymphadenitis with diffuse lymphoid depletion and numerous MGCS with rare ICIBs and edema, necrosis, leukocytosis, histiocytosis; Vascularization.  **Diaphragm**: Scattered single cell acute myodegeneration with hypercontraction and segmental hyaline necrosis; Multifocal acute hemorrhage.  **Liver**: Marked, focally extensive, subacute to chronic necrotizing, granulomatous and proliferative cholangitis with necrotizing vasculitis and thrombosis, numerous INCIBs in bile epithelium and granulation tissue; Scattered acute random neutrophilic infiltrates with histiocytes and lymphoplasmacytic pericholangitis; Mild, diffuse hepatocellular atrophy, vacuolar change and congestion; Mild multifocal Kupffer cell hypertrophy with hemosiderosis.  **Pancreas**: Marked, multifocal, subacute to chronic necrotizing pancreatitis and ductitis with numerous MGCS, occasional intraepithelial INCIBs, necrotizing vasculitis, fibrinoid wall necrosis, edema, hemorrhage and granulation tissue.  **Mesenteric lymph node**: Moderate to marked, histiocytic lymphadenitis with numerous MGCS with rare INCIBs and lymphoid depletion with lymphocytolysis, edema, sinus leukocytosis and histiocytosis.  **Keratinized stomach**: Marked, focally extensive, chronic proliferative and fibrosing gastritis with MGCS and adult anisakid nematode remnants associated with localized granulomatous reaction; Marked, diffuse fibropapillomatous hyperplasia.  **Glandular stomach**: Mild, multifocal, chronic lymphocytic gastritis with mucous cell hyperplasia.  **Pyloric stomach**: Marked, multifocal, chronic proliferative and fibrosing gastritis with scattered MGCS, anisakid remnants and MATMHH.  **Kidney**: Mild to moderate, multifocal, acute tubular degeneration; Diffuse congestion with scattered acute hemorrhage; Scattered INCIBs in renicular urothelium.  **Adrenal gland**: Moderate, multifocal, acute subcapsular and scattered parenchymal hemorrhage with single cell acute cortical necrosis; Scattered lymphoplasmacytic cortico-medullary interstitial infiltrates; Diffuse congestion and rare fibrin microthrombi.  **Prescapular lymph node:** severe follicular depletion with MGCS, hyalinosis and fibrosis; Paracortical hyperplasia; MATMHH and mild vascularization.  **Scapulohumeral synovium**: Severe, focally extensive, chronic necrosuppurative and granulomatous synovitis with fibroplasia, rare bacteria and numerous MGCS.  **Cerebellum**: Marked, multifocal, subacute to chronic lymphohistiocytic meningoencephalitis with perivascular cuffing, gliosis and perivascular edema.  **Spinal cord**: Marked, multifocal, chronic lymphohistiocytic meningopoliomyelitis with perivascular cuffing, marked gliosis, glial nodules, white matter spongiosis and polirradiculoneuritis.  **Vestibulocochlear nerve**: Marked, multifocal, chronic lymphohistiocytic cranial neuritis with perivascular cuffing, spongiosis and Wallerian degeneration.  **Cerebrum**: Marked, multifocal, chronic lymphohistiocytic to granulomatous meningoencephalitis with gliosis, MGCS, satellitosis, glial degeneration and necrosis, rarefaction/liquefactive necrosis, hemorrhage and vasculitis.  **Brainstem, pons**: Marked, multifocal, chronic lymphohistiocytic meningoencephalitis and granulomatous ventriculitis with MGCS, perivascular cuffing, gliosis, hemorrhage, spongiosis and Wallerian degeneration.  **Hypophysis**: Marked, multifocal, chronic lymphohistiocytic meningitis and neurohypophisitis with astrocytosis; Focal lymphocytic adenohypophysitis. | Subacute systemic CeMV and suspect *Brucella* coinfection |
| 14 | Mild epiobiosis by *Syncyamus* sp. along the blowhole; Multifocal linear and triangular net-like erosions and lacerations throughout the body surface; Focal skin erosion caudal to blowhole; Focal mandibular and maxillary fracture with tooth loss and hemorrhage; Focal rib fracture; Scarce ingesta (few otoliths); Marked bilateral emphysema with rib impressions; Multifocal subpleural granulomas and atelectasia. | **Cerebrum:** Marked, multifocal, chronic lymphocytic meningoencephalitis with perivascular cuffing, gliosis, satellitosis, neuroglial and neuronal degeneration, neuronophagia, perivascular edema, spongiosis and rare INCIBs.  **Cerebellum**: Mild, focal, chronic lymphocytic meningitis, mild gliosis and scattered white matter spongiosis.  **Spinal cord**: Mild, focal, chronic lymphocytic poliomyelitis with perivascular cuffing; Multifocal acute epidural hemorrhage.  **Pons, thalamus**: Mild, multifocal, chronic lymphocytic meningoencephalitis with gliosis, astrocytosis, glial nodules.  **Hypophysis**: Multifocal acute hemorrhage.  **Skin**: Minimal epidermal hyperplasia and rare ICIBs (suggestive of *CetaceanPoxvirus*).  **Skeletal muscle**: Rare hypercontracted myofibers; Scattered atrophied myofiber groups.  **Rectum**: Moderate, multifocal, chronic lymphohistiocytic and eosinophilic cryptitis with dilatation and metacestodes.  **Liver**: Mild, diffuse hepatocellular atrophy and sinusoidal congestion; Scattered portal lymphocytes.  **Vagina**: Mild, multifocal lymphocytic nodular vaginitis.  **Glandular stomach**: Minimal, multifocal lymphocytic gastritis.  **Colon**: Minimal to mild, mutifocal, chronic colitis.  **Pyloric stomach**: Mild, multifocal, chronic lymphoplasmacytic nodular gastritis.  **Kidney**: Diffuse congestion.  **Hypophysis**: Multifocal lymphocytic meningitis.  **Spleen**: Mild, diffuse lymphoid reactive hyperplasia; Diffuse congestion.  **Mediastinal lymph node**: Mild, diffuse lymphoid reactive hyperplasia; Mild, multifocal eosinophilic lymphadenitis with sinus histiocytosis.  **Lung**: Diffuse alveolar emphysema; Minimal to mild, multifocal lymphocytic interstitial pneumonia; Moderate bronchiolar mineralization.  **Urinary bladder, Uterine horn, Ovary, Urethra, Adrenal gland, Thymus, Vestibulocochlear nerve**: NSLO | Entanglement; trauma; Acute systemic CeMV infection  (Fishing interaction) |
| 15 | Maxillary and mandibular skin erosion; Multifocal live stranding-related linear erosions, abrasions and lacerations on the ventral peduncle and anogenital region; Marked bilateral thoracolumbar subcutaneous edema; Moderate infestation by *P. delphini* merocercoids in the anogenital region and peduncle; Focal transverse maxillary and mandibular fracture (rostral third); Multifocal erosivo-ulcerative glossitis; Scarce ingesta (squid beaks); Multifocal erosive-ulcerative (glandular and pyloric) gastritis; Moderate parasitization by *M. grimaldi* merocercoids throughout abdominal serosae; Bilateral patchy pulmonary atelectasia; Mild bronchial parasitization by *Stenurus* sp. nematodes; Generalized lymphadenomegaly. | **Skin**: Moderate, multifocal, chronic granulomatous deep dermatitis and panniculitis with abundant phagocytosed and extracellular ceroid-like yellow pigment and hemorrhage; Scattered epithelial hyperplasia.  **Skeletal muscle**: Scattered acute myocyte hypercontraction and rare segmental hyaline degeneration.  **Lung**: Moderate, multifocal, chronic lymphoplasmacytic bronchointerstitial pneumonia with focal bronchial sclerosis and intralesional nematode remnants, alveolar histiocytes, scattered MGCS with INCIBs and edema; Multifocal angiomatosis; Multifocal bronchiolar mineralization.  **Mediastinal lymph node**: Mild to moderate, multifocal follicular depletion with hyalinosis, mild paracortical hyperplasia and hemosiderosis.  **Prescapular lymph node**: Mild to moderate, diffuse paracortical and medullary hyperplasia with sinus histiocytosis, erythrocytosis and histiocytosis.  **Heart**: Scattered hypereosinophilic cardiomyocytes; Congestion.  **Liver**: Mild, multifocal, chronic lymphoplasmacytic pericholangitis and cholangitis with mild focal bile duct hyperplasia and focal intraductal metacestodes; Moderate, diffuse congestion with scattered hemorrhage and centrilobular hepatocellular atrophy.  **Pancreas**: Diffuse zymogen granule depletion.  **Keratinized stomach**: Mild, multifocal mucosal hyperplasia with intracellular edema and microvesicles.  **Glandular stomach**: Focal subacute necroerosive apical gastritis with hemorrhage; Focal serosal lymphocytic perivasculitis.  **Pyloric stomach, Small and Large intestine**: Mild, multifocal, chronic lymphocytic and eosinophilic gastritis and enterocolitis; Severe, focally extensive suppurative colonic mesenteritis with vasculitis and necrosis.  **Mesenteric lymph node**: Mild, multifocal eosinophilic lymphadenitis; Mild, multifocal follicular lymphoid depletion.  **Kidney**: Minimal, multifocal, chronic lymphocytic interstitial nephritis with rare glomerulosclerosis; Mild, multifocal, acute tubular degeneration.  **Thyroid**: Diffuse congestion and scattered acute hemorrhage.  **Spleen**: Mild, multifocal lymphoid reactive hyperplasia with follicular depletion and hyalinosis, sinus histiocytosis and hemosiderosis; Congestion.  **Paravertebral/retroperitoneal lymph nodes**: Moderate, diffuse, lymphoid hyperplasia with plasmacytosis and histiocytosis; Multifocal, acute pericapsular hemorrhage.  **Tongue**: Mild, multifocal ulcerative glossitis with hemorrhage, mild hyperplasia and intracellular edema, fibrin microthrombi and vascular necrosis.  **Cerebrum, thalamus, cerebellum**: Moderate to marked (thalamus, cerebellum), multifocal, chronic lymphocytic meningoencephalitis with perivascular cuffing, gliosis, satellitosis, perivascular edema, spongiosis, Wallerian degeneration and rare INCIBs.  **Spinal cord**: Mild, multifocal lymphocytic poliomyelitis; Multifocal meningeal/epidural acute hemorrhage.  **Vestibulocochlear nerve**: Minimal, multifocal, chronic lymphocytic neuritis.  **Adenohypophysis**: Diffuse congestion. | Acute systemic CeMV infection |
| 16 | Marked bilateral cervicothoracic edema; Mild subcutaneous (anogenital and peduncle) parasitization by *P. delphini* merocercoids; Teeth loss; Lack of ingesta; Multifocal, chronic granulomatous (glandular and pyloric) gastritis with *P. gastrophilus* trematodes and mild luminal obliteration; Mild intestinal infestation by adult Diphyllobothridae cestodes; Hepatic congestion; Severe pancreatic infestation by Brachycladiidae trematodes in pancreatic ducts and duodenal ampulla, stomach and intestine; Moderate parasitization by *M. grimaldi* merocercoids throughout abdominal serosae; Moderate, multifocal, acute renal subcapsular hemorrhages. | **Cerebrum**: Moderate to marked, multifocal, chronic lymphocytic meningoencephalitis with perivascular cuffing, gliosis and rare INCIBs.  **Skeletal muscle**: Mild, multifocal, acute hypercontraction and segmental hyaline degeneration.  **Lung**: Marked, diffuse, acute pulmonary edema with hemorrhage; Mild, multifocal, chronic lymphocytic interstitial pneumonia with mild fibrosis; Diffuse congestion; Multifocal bronchiolar mineralizations.  **Mediastinal lymph node**: Mild, diffuse lymphoid depletion and sinus edema; congestion.  **Prescapular lymph node**: Mild lymphoid reactive hyperplasia with scattered follicular depletion.  **Pancreatic lymph node**: Mild, multifocal, chronic eosinophilic and histiocytic lymphadenitis with numerous Brachycladiidae trematode eggs and fibrosis; Diffuse lymphoid depletion with histiocytosis and hemosiderosis.  **Liver**: Minimal, multifocal, chronic lymphocytic pericholangitis; Diffuse congestion.  **Small and large intestine**: Scattered lymphocytic and eosinophilic infiltrates.  **Glandular stomach**: Minimal, multifocal, chronic lymphocytic gastritis with superficial *P. gastrophilus* trematode eggs; Focal arteriosclerosis (elastosis) with obliteration.  **Pyloric stomach**: Marked, focally extensive, chronic pyogranulomatous gastritis with fibrosis, necrosis, adult trematodes and numerous *P. gastrophilus* eggs and mucosal hyperplasia.  **Pancreas**: Severe, chronic pancreatic ductitis with obliteration and fibrosis.  **Main pancreatic duct:** Chronic lymphohistiocytic ductitis with mucinous cystic hyperplasia and cryptitis and Brachycladiidae trematode eggs.  **Mesenteric lymph node**: Diffuse lymphoid depletion with follicular depletion, scattered hyalinosis and sinus histiocytosis; Mild, multifocal, chronic eosinophilic nodular lymphadenitis with necrosis.  **Kidney**: Scattered acute tubular degeneration; Diffuse congestion.  **Thyroid**: Diffuse congestion.  **Adrenal**: Scattered lymphocytic infiltrates.  **Spleen**: Mild, multifocal follicular lymphoid depletion; Diffuse congestion.  **Urinary bladder, Skin**: NSLO. | Chronic localized brain CeMV infection |
| 17 | Numerous intra-/interspecific interaction and active stranding-related marks; Moderate infestation by *P. delphini* merocercoids in anogenital and peduncle;  Multifocal hemorrhage in ventral cervical and cephalic musculature; Tooth wear and loss; Multifocal hyperkeratosis in the oropharynx and esophagus; Lack of ingesta; Focal luminal wire and multiple mucosa ulcers in the keratinized stomach  Multifocal hemorrhagic ulcers and focal granuloma with adult *P. gastrophilus* trematodes in pyloric stomach; Moderate pancreatic infestation by *Brachycladiidae* trematodes; Hepatic congestion; Moderate parasitization by *M. grimaldi* plerocercoids throughout abdominal serosae; Multifocal granulomatous bronchopneumonia with adult nematodes; Multifocal emphysema and atelectasia; Focal accessory spleen; Luminal hemorrhagic coagule in right pterygoid sinus. | **Skin**: Mild, multifocal, acute hemorrhage in subcutis; Minimal epidermal hyperplasia.  **Skeletal muscle**: Scattered, multifocal myocyte necrosis with satellitosis and rare phagocytosis.  **Mammary gland muscle**: Numerous intrasarcoplasmic protozoal cysts (*Sarcocystis* sp.); Multifocal acute hemorrhage.  **Lung**: Moderate, multifocal, chronic bronchointerstitial pneumonia with scattered bronchial sclerosis, chondroid necrosis, fibrosis, severe remodeling, epithelial metaplasia and nematode remnants; Abundant bronchiolar mineralizations; Alveolar edema and hemorrhage with aspirated squames; Angiomatosis.  **Mediastinal lymph node**: Diffuse lymphoid depletion with follicular hyalinosis and mild histiocytosis; Mild to moderate, multifocal, chronic eosinophilic lymphadenitis and fibrosis.  **Prescapular lymph node**: Diffuse cortical depletion with mild paracortical and medullary cord hyperplasia (plasmacytosis), marked sinus histiocytosis and scattered MGCS.  **Heart**: Moderate, multifocal, acute cardiomyocyte degeneration, scattered microhemorrhage and contraction band necrosis.  **Liver**: Mild, multifocal, chronic lymphohistiocytic pericholangitis with hemosiderosis, mild portal fibrosis and bile duct hyperplasia; Multifocal acute centrilobular congestion.  **Pancreas**: Severe chronic proliferative and lymphoplasmacytic pancreatic ductitis with cystadenomatous mucosal hyperplasia and adult/larval *Brachycladiidae* trematodes and focal metacestode; Multifocal parenchymal hemorrhage.  **Pyloric stomach**: Marked, focal, chronic granulomatous and fibrosing gastritis with numerous *P. gastrophilus* trematode eggs and adults, MATMHH and diffuse mucosal hyperplasia; Hyperplastic and lymphoplasmacytic gastritis.  **Glandular stomach**: Moderate, focal, chronic granulomatous and fibrosing nodular gastritis with *P. gastrophilus* trematode eggs, focal anisakid remnants and diffuse mucosal hyperplasia.  **Pyloric stomach, Small intestine**: Mild, multifocal, chronic lymphoplasmacytic gastroenteritis with scattered necrosis; Occasional mineralization.  **Small intestine**: Mild, multifocal, chronic eosinophilic and lymphoplasmacytic enteritis.  **Mesenteric lymph node**: Moderate, diffuse, chronic eosinophilic lymphadenitis, sinus histiocytosis; follicular/cortical depletion mild.  **Kidney**: Diffuse congestion with multifocal acute tubular degeneration, scattered microhemorrhage, hemoglobin casts and mild interrenicular edema.  **Urinary bladder**: Minimal, multifocal, acute cystitis with hemorrhage, apical epithelial necrosis and exocytosing neutrophils and rare lymphocytes.  **Hypophysis**: Diffuse congestion and multifocal meningeal hemorrhage; Marked, multifocal mineralization.  **Cerebrum**: Moderate, multifocal, chronic lymphocytic meningoencephalitis with acute neuronal and neuroglial degeneration, spongiosis, perivascular cuffing, edema, congestion and hemorrhage; Focal perivascular histiocytosis with ceroid pigment.  **Choroid plexus**: Scattered perivascular lymphocytic infiltrates; Hyalinosis.  **Cerebellum**: Minimal, multifocal encephalitis with perivascular cuffing, gliosis, hemorrhage, neuronal degeneration, Bergmann’s glia hyperplasia.  **Oral mucosa**: Mild hyperplasia with scattered hydropic degeneration; Scattered submucosal lymphocytic infiltrates.  **Adrenal gland**: Diffuse congestion, scattered acute hemorrhage and multifocal acute cortical degeneration.  **Mammary gland**: Minimal, multifocal, chronic lymphocytic mastitis with scattered neutrophilic exudate in galactophorous ducts and few MGCS.  **Spleen**: Mild follicular depletion and hyalinosis; Diffuse congestion with scattered capsular hemorrhage; Minimal extramedullary hematopoiesis. | Chronic localized brain CeMV infection |
| 18 | Multifocal edema and hematomas in periocular, otic and ventrocervical subcutis; Mild epibiosis by *Xenobalanus* sp. in caudal fin; Moderate parasitization by *P. delphini* merocercoids in ventroabdominal and perigenital areas; Multifocal petechiae in melon’s fat; Multifocal ulcerative glossitis (left canthus); Multifocal linear ulcers in keratinized stomach; Multifocal petechiae and ecchymoses in glandular stomach; Small amount of partially digested ingesta (squid beaks) in keratinized and glandular stomach; Multifocal pancreatic petechiae; Severe infestation by *M. grimaldi* merocercoids in caudal axial muscles; Multifocal chronic interstitial nephritis; Generalized lymphadenomegaly. | **Cerebrum, thalamus**: Mild to marked (thalamus), multifocal, chronic lymphocytic meningoencephalitis with marked gliosis and glial nodules, neuroglial degeneration, perivascular edema, hemorrhage, neuroparenchymal rarefaction, spongiosis.  **Cerebellum, pons**: Minimal to marked (pons), multifocal, chronic lymphocytic meningoencephalitis with white matter spongiosis and perivascular cuffing.  **Spinal cord**: Marked, multifocal lymphocytic polioleukomyelomeningitis with marked gliosis, perivascular cuffing, hemorrhage and meningoradiculitis.  **Skin**: Moderate, focal, chronic pyogranulomatous dermatitis with *Pennella* sp. remnant; Mild, multifocal epidermal hyperplasia with multifocal ICIBs (compatible with *CetaceanPoxvirus*).  **Skeletal muscle**: Scattered acute single cell hypercontraction; Scattered single cell atrophy; Focal myocyte basophilic degeneration.  **Lung**: Marked, multifocal, chronic suppurative and eosinophilic to pyogranulomatous and sclerosing bronchopneumonia with interstitial lymphoplasmacytic infiltrates, type II pneumocyte hyperplasia, scattered MGCS, edema, fibrin, fibrosis, chrondronecrosis and loss and bronchiolitis obliterans; Angiomatosis and MATMHH; Moderate bronchiolar mineralizations.  **Adrenal gland**: Multifocal, acute cortical hemorrhage with single cell degeneration; Scattered cortical myxoid degeneration.  **Tongue**: Mild, multifocal, subacute ulcerative glossitis with scattered microthrombi, necrosis, hemorrhage and superficial bacteria.  **Placenta**: Minimal to mild, multifocal lymphocytic placentitis with multifocal hemorrhage, necrosis and equivocal ICIBs.  **Kidney**: Mild, multifocal, acute tubular degeneration; Rare tubular proteinosis; Mild, multifocal chronic lymphocytic interstitial nephritis with fibrosis.  **Pancreas**: Multifocal, acute hemorrhage and necrosis; Mild, multifocal lymphoplasmacytic interstitial pancreatitis with follicle formation and focal intraductal Brachycladiidae trematode egg; Multifocal, acute hemorrhage.  **Pancreatic duct**: Marked, segmental, chronic proliferative ductitis with adult Brachycladiidae trematode.  **Hypophysis**: Diffuse congestion and scattered peripheral hemorrhage.  **Keratinized stomach**: Focal, acute serosal hemorrhage; Mild, multifocal mucosal hyperplasia.  **Oropharynx**: Mild, multifocal, chronic lymphoplasmacytic oropharyngitis with mild mucosal hyperplasia.  **Tonsil**: Mild, multifocal, chronic lymphoplasmacytic tonsillitis with mild epithelial hyperplasia, focal ulcer and glandular ectasia.  **Esophagus**: Multifocal, acute hemorrhage; Scattered perivascular lymphocytic infiltrates in submucosa.  **Glandular and Pyloric stomach**: Mild, multifocal, chronic lymphoplasmacytic gastritis with mucosal hyperplasia and focal intralesional *P. gastrophilus* trematode egg and hemorrhage  **Perirrenal lymph node**: Marked, multifocal, chronic granulomatous lymphadenitis with abundant iron-porphyrin (trematode) pigment, Paracortical hyperplasia and hemorrhage; Diffuse lymphoid hyperplasia; Marked sinus erythrocytosis and sinus histiocytosis with rare Brachycladiidae trematode eggs.  **Spleen**: Mild, diffuse lymphoid depletion; Multifocal acute hemorrhage; Mild, multifocal fibrosis and rare hemosiderosis; Focal mineralization.  **Trachea**: Mild, multifocal, chronic lymphoplasmacytic tracheitis.  **Mediastinal lymph node**: Diffuse eosinophilic lymphadenitis with scattered granulomas, leukophagia, erythrophagocytosis; Mild diffuse paracortical lymphoid hyperplasia, sinus edema, histiocytosis, and rare MGCS.  **Peritracheal lymph node**: Eosinophilic lymphadenitis with mild sinus histiocytosis and hemosiderosis; Mild diffuse lymphoid hyperplasia.  **Choroid plexus**: Diffuse congestion; Scattered subepithelial mineralization.  **Pregnant uterus**: Marked, focal, acute mucosa hemorrhage.  **Prescapular lymph node**: Mild, diffuse eosinophilic lymphadenitis with scattered granulomatous inflammatory foci associated with ceroid pigment, sinus histiocytosis and leukophagocytosis; Mild, diffuse follicular depletion.  **Prescapular lymph node**: Granulomatous lymphadenitis with yellow globular phagocytosed and extracellular material, sinus histiocytosis and hemorrhage; Diffuse lymphoid hyperplasia; Multifocal (perinodal) basophilic myocyte degeneration.  **Urinary bladder**: Scattered exocytosing neutrophils and rare ICIBs.  **Small and Large intestine**: Mild, multifocal, chronic eosinophilic and lymphoplasmacytic enterocolitis with scattered aucte hemorrhage.  **Trachea**: Mild, diffuse lymphoplasmacytic tracheitis.  **Larynx**: Focal luminal cestode merocercoid.  **Pancreatic lymph node**: Mild, multifocal eosinophilic lymphadenitis, interstitial fibrosis, scattered sinus *Brachychladiidae* trematode eggs; Mild, diffuse lymphoid reactive hyperplasia.  **Liver**: Mild, multifocal, chronic portal hepatitis with fibrosis.  **Choledochus duct**: Lymphocytic ductitis with fibrosis and intraductal adult Brachycladiidae.  **Mesenteric lymph node**: Mild, multifocal eosinophilic lymphadenitis with sinus histiocytosis and hemosiderosis.  **Cervical lymph node**: Mild, multifocal, chronic eosinophilic lymphadenitis with mild medullary cord lymphoid hyperplasia and sinus histiocytosis.  **Ovary,** **Umbilicus**: NSLO. | Chronic systemic CeMV infection |
| 19 | Multifocal hematomas and suffusive hemorrhage in right retro-infraorbital, ventrocervical, pectoral and fins, dorsal thorax and ventral abdomen, compatible with blunt polytraumatism; Ulcer and soft tissue loss of rostrum with hemorrhage;  Multifocal intraspecific interaction dorsal, bilateral marks; Irregular, linear non-parallel cuts with hyperemia; Multiple squid tentacle marks on right buccal commissure; Mild epibiosis by Xe*nobalanus* sp. in caudal fluke; Moderate subcutaneous parasitization by *P. delphini* merocercoides in anogenital area;  Multifocal subcutaneous and muscle hemorrhage in right occipital, temporal, right mandible and basooccipital areas; Mild serosanguineous fluid in left scapulohumeral joint; Moderate ascites; Generalized icterus; Multifocal tongue ulcers; Scarce digested ingesta (squid beaks, otoliths); Multifocal erosions in keratinized and glandular stomachs with occasional ulceration and hemorrhage;  Moderate parasitization by anisakids and cestodes in pyloric stomach; Diffuse reticular pattern and dilated bile ducts;  Multifocal hemorrhage; Moderate parasitization by *Brachycladiidae* trematodes in pancreas and main pancreatic duct; Moderate infestation by *M. grimaldi* merocercoids throughout abdominal serosae; Diffuse emphysema with multifocal atelectasia, parenchymal hemorrhage and subpleural and parenchymal granulomas associated with mild bronchial nematodiasis (left lung); Diffuse congestion of right lung.  Dilated right cardiac ventricle with flaccid walls; Multifocal interrenicular and parenchymal granulomas; Mild red-tinged urine; Generalized lymphadenomegaly;  Pyogranulomatous pulmonary lymphadenitis; Subcapsular and parenchymal pancreatic/gastrohepatic lymph node hemorrhage; Severe, suffusive intracranial and dural ventrocranial hemorrhage in cerebrum and leptomeningeal congestion. | **Cerebrum, Cerebellum, Spinal cord**: Marked, multifocal, chronic lymphocytic meningoencephalomyelitis, poliomyelomeningitis and radiculoneuritis with MGCS, INCIBS, perivascular cuffing, gliosis, satellitosis, neuronophagia and hemorrhage.  **Lung**: Marked, multifocal, chronic pyogranulomatous bronchopneumonia with adults and larval nematodes, bacteria and hemorrhage; Moderate, multifocal, subacute interstitial pneumonia with type II pneumocyte hyperplasia, scattered MGCS and INCIBs and mild fibrosis; Moderate bronchiolar mineralization.  **Skin**: Mild, multifocal, chronic regular epidermal hyperplasia with rare ICIBs (compatible with *CetaceanPoxvirus*); Superficial dermal congestion.  **Skeletal muscle** (**including diaphragm)**: Mild, multifocal, acute myocyte degeneration with segmental hyaline necrosis.  **Laryngeal tonsil**: Moderate, multifocal, chronic tonsillitis with lymphoid depletion, lymphocytolysis and mild neutrophilic crypt exudate and necrosis.  **Adrenal gland**: Diffuse congestion with scattered hemorrhage and single cell cortical degeneration.  **Epididymis**: Minimal, multifocal luminal neutrophilic exudate with scattered single cell necrosis.  **Keratinized stomach**: Multifocal, irregular, chronic mucosal hyperplasia.  **Glandular stomach**: Mild to moderate, multifocal, acute to subacute erosive-hemorrhagic gastritis.  **Tongue:** Marked, focally extensive, chronic pyogranulomatous and fibrosing glossitis with osseous foreign body, myofiber degeneration, necrosis, loss and regeneration; Mild mucosal hyperplasia with rare INCIBs; Lumina hemorrhage.  **Penis, Urethra**: Mild, multifocal mucosal hyperplasia with scattered hydropic change and minimal lymphocytic infiltrates.  **Liver**: Diffuse congestion, hepatocellular single cell necrosis and abundant cytoplasmic eosinophilic globules and *pink points*; Focal acute fibrinocellular thrombus with vascular necrosis; Mild, multifocal, acute bile duct epithelial necrosis with scattered INCIBs and minimal lymphocytic and neutrophilic pericholangitis.  **Heart**: Mild valvular endocardiosis (myxoid valvular degeneration); Mild, multifocal, acute cardiomyocyte degeneration with contraction band necrosis; Scattered epicardial acute hemorrhage.  **Eye**: Rare eosinophilic INIBs eosinophilic inclusion bodies.  **Kidney**: Marked, multifocal, acute pyelonephritis with necrosis, cellular casts, rare syncytia, tubular proteinosis and potential ICIBS; Multifocal, acute tubular necrosis with hemoglobinuria; Scattered glomerulosclerosis; Scattered tubular regeneration with tubular cysts and cholesterol clefts; Mild, multifocal tubular mineralization.  **Small intestine**: Mild, multifocal, chronic eosinophilic enteritis.  **Rectum**: Mild, multifocal, chronic eosinophilic proctitis with crypt neutrophilic exudate, merocercoids and hyalinosis.  **Pancreatic lymph node**: Moderate, multifocal, chronic pyogranulomatous and eosinophilic lymphadenitis with hemorrhage; Rare sinus mineralization. **Perythyroid, Prescapular lymph node**: Mild, diffuse, chronic eosinophilic lymphadenitis with sinus histiocytosis.  **Mesenteric, Gastrohepatic lymph node**: Mild, diffuse, chronic eosinophilic lymphadenitis with marked lymphoid depletion, sinus histiocytosis and hemosiderosis.  **Retropharyngeal lymph node**: Mild, multifocal, chronic lymphoid depletion with scattered lymphocytolysis; Diffuse sinus histiocytosis.  **Spleen**: Mild, diffuse lymphoid depletion with lymphocytolysis, histiocytosis and hemosiderosis; Extramedullary hematopoiesis.  **Urinary bladder**: Arterial medial siderocalcinosis.  **Testicle**: NSLO | Acute systemic CeMV infection |
| 20 | Multifocal cutaneous excoriations in lateroventral aspects, perigenital, buccal, and periorbital; Bilateral ocular sinking; Multifocal hemorrhage in mesenteric lymph node; Diffuse hepatomegaly; Ascites; Diffuse bilateral pulmonary congestion and edema; Brain congestion with scattered hemorrhage. | **Cerebrum**: Mild, multifocal lymphohistiocytic encephalitis with focal necrosis, scattered INCIBs and gliosis; Moderate, multifocal acute meningeal and perivascular parenchymal hemorrhage and edema.  **Spleen**: Diffuse congestion; Mild, diffuse lymphoid reactive hyperplasia with occasional follicular lymphoid depletion, follicular hyalinosis and sinus histiocytosis; Moderate, multifocal perivascular siderocalcinosis; Mild, focal, chronic granulomatous serositis.  **Lung**: Mild, multifocal, chronic lymphocytic bronchointerstitial pneumonia; Diffuse congestion and mild edema. | Chronic localized brain CeMV infection |
| 21 | NR | **Liver**: Mild, multifocal, chronic lymphoplasmacytic pericholangitis with sinusoid leukocytosis, hemosiderosis and lipofuscin; Mild, diffuse congestion with scattered centrilobular hemorrhage.  **Large intestine**: Mild to moderate, multifocal, chronic eosinophilic colitis with follicular hyalinosis.  **Spleen**: Mild, multifocal lymphoid hyperplasia with follicular hyalinosis, sinus histiocytosis, rare MGCS, hemosiderosis; Mild congestion and multifocal, acute hemorrhage; Focal vascular siderocalcinosis; Minimal, multifocal eosinophilic splenitis; Mild, multifocal arteriolosclerosis and arteriosclerosis.  **Skin**: Mild, multifocal epidermal hyperplasia with rare ICIBs (suggestive of *CetaceanPoxvirus*).  **Heart**: Mild congestion; Scattered hypercontracted cardiomyocytes.  **Lung**: Mild, multifocal, chronic lymphocytic interstitial pneumonia with alveolar histiocytosis, mild fibrosis, hemosiderophages, multifocal chondrodegeneration and chrondronecrosis; Mild edema; Mild mineralization; Minimal angiomatosis.  **Testicle, epididymis**: Minimal to mild, multifocal, chronic interstitial lymphocytic orchitis with scattered seminiferous cell necrosis and hemorrhage.  **Cerebrum (cortex)**: Minimal, focal lymphocytic perivascular cuffing; Mild, multifocal astrocytosis with acute neuronal degeneration and satellitosis; Mild, multifocal spongiosis in white matter.  **Adrenal gland**: Focal adrenocortical cyst with marked subacute hemorrhage and fibrosing adrenalitis and ischemic necrosis; Mild, multifocal arteriolosclerosis and arteriosclerosis.  **Kidney**: Mild, multifocal, chronic mesangial/membranous glomerulopathy with Bowman’s capsule fibrosis, tubular ectasia, proteinosis, rare myo/hemoglobin casts, hyaline droplets, nephron atrophy and scattered tubular regeneration; Mild, multifocal, acute tubular degeneration and necrosis with rare INCIBs; Marked, multifocal, chronic medullary tubular mineralization; Rare INCIBs in urothelium. | Acute systemic CeMV infection |
| 22 | NR | **Liver**: Minimal to mild, multifocal, chronic lymphoplasmacytic pericholangitis with mild leukocytosis; Mild, multifocal to diffuse hepatocyte vacuolar change; Mild, diffuse congestion.  **Skin**: Minimal, multifocal epidermal hyperplasia with scattered ICIBs in basal keratinocytes.  **Heart**: Mild, multifocal, chronic interstitial myocardial fibrosis; Scattered cardiomyocyte vacuolization.  **Lung**: Minimal to mild, chronic interstitial pneumonia with mild fibrosis.  **Ovary**: Minimal, focal lymphohistiocytic interstitial infiltrate.  **Mediastinal lymph node**: Moderate, diffuse lymphoid hyperplasia with moderate sinus histiocytosis and scattered sinus *Brachycladiidae* trematode eggs.  **Spleen**: Mild, diffuse lymphoid hyperplasia, sinus histiocytosis and minimal hemosiderosis; Mild, diffuse congestion.  **Kidney**: Mild congestion; Mild, multifocal tubular proteinosis and rare tubular mineralization.  **Pancreas, Uterus, Small intestine, Cerebrum (cortex)**: NSLO. | Chronic systemic CeMV infection |
| 23 | NR | **Cerebrum, Cerebellum**: Minimal (cerebellum) to mild (cerebrum), multifocal lymphocytic meningoencephalitis with perivascular cuffing, gliosis/astrocytosis, rare INCIBs; Diffuse congestion and perivascular edema.  **Lung**: Mild to moderate, multifocal, chronic lymphocytic bronchointerstitial pneumonia with bronchitis, BALT hyperplasia, pleuritis, interstitial and pleural fibrosis; Moderate to marked, diffuse edema with alveolar histiocytosis; Mild, multifocal angiomatosis; Mild, multifocal bronchiolar mineralization.  **Pulmonary lymph node**: Mild to moderate, diffuse lymphoid reactive hyperplasia with sinus histiocytosis and rare MGCS. | Chronic systemic CeMV infection |
| 24 | NR | **Liver**: Mild to moderate, multifocal, chronic lymphoplasmacytic pericholangitis with moderate to marked sinusoid leukocytosis (identify these cells; mimicking reactive hepatitis), Kupffer cell hypertrophy, hemosiderosis and cholestasis (rule out mercury); Mild, diffuse congestion.  **Lung**: Mild, multifocal, chronic bronchointerstitial pneumonia and mild edema.  **Spleen**: Mild, multifocal lymphoid hyperplasia with moderate, diffuse sinus hemosiderosis and histiocytosis.  **Cerebellum**: Minimal, multifocal perivascular edema with astrocytic swelling.  **Kidney**: Scattered chronic interstitial lymphocytic infiltrates; Scattered tubular ectasia, proteinosis and tubular mineralization with tubuloepithelial degeneration and loss; Congestion.  **Colon, Heart**: NSLO. | Chronic systemic CeMV infection |
| 25 | Multifocal intraspecific cutaneous erosions and lacerations; Cephalic laterodorsal cutaneous proliferative/ulcerative lesions; Tattoo-like skin lesion on right lateral peduncle; *P. delphini* calcified pyogranulomas in caudoventral subcutis; Mild, diffuse hyperplastic stomatitis, palatitis and pharyngitis; Pulmonary edema; Prescapular lymphadenomegaly; Tricavitary effusion (hydrothorax, hydropericardium, ascites); Right lung necrossupurative bronchopneumonia with hemorrhage; Right pulmonary lymphadenomegaly; Pale and emphysematous left lung; Endocardial and epicardial petechiae; Focal *M. grimaldii* granulomatous abdominal serositis; Diffuse hepatomegaly and splenomegaly with petechiae; Few anisakids in keratinized stomach; Lack of ingesta; Diffuse gastric hyperemia; Mild, multifocal granulomatous pyloric gastritis by *P. gastrophilus*; Diffuse serocatarrhal enteritis with mucosa/submucosa edema; Mesenteric lymphadenomegaly; Mild, bilateral nephromegaly with edema and congestion;  Diffuse cerebral edema with hyperemia/congestion and mild ventricular dilatation (acquired hydrocephalus); Suppurative nodular choroid plexitis and ventriculitis. | **Pharynx**: Mild to moderate, multifocal, chronic erosive pharyngitis with salivary gland ectasia.  **Tongue, Salivary gland**: Mild, multifocal, chronic glossitis with crypt inspissation by mineralized secretory material and rare INCIBs.  **Skin**: Multifocal granulomatous dermatitis and panniculitis with foreign body, fibrosis and moderate irregular epidermal hyperplasia; Marked, multifocal, chronic pyogranulomatous dermatitis and panniculitis with presumed mineralized nematode eggs and overlying epidermal hyperplasia.  **Glandular stomach**: Minimal, multifocal, chronic lymphocytic gastritis with rare INCIBs.  **Pyloric stomach**: Minimal to mild, multifocal, chronic lymphocytic gastritis with single cell epithelial necrosis and INCIBs; Mild, focal, chronic granulomatous gastritis with mineralization.  **Lung**: Marked, multifocal to coalescing, subacute to chronic suppurative bronchopneumonia with bronchiectasis, abscess formation, necrosis and Gram-positive bacteria with Splendore-Hoeppli reaction, type II pneumocyte hyperplasia, marked histiocytosis, MGCS with rare INCIBs, bronchial sclerosis, mucosal hyperplasia, lymphoid proliferation, marked fibrosis and remodeling; Angiomatosis and MATMHH.  **Skeletal muscle**: Acute, multifocal myocyte degeneration.  **Thyroid**: Mild congestion; Mild, multifocal interstitial fibrosis.  **Kidney**: Minimal to mild, multifocal, acute neutrophilic glomerulitis and tubulitis; Scattered acute tubular degeneration; Mild, multifocal tubular proteinosis and scattered medullary mineralization, congestion.  **Cerebellum**, **Cerebrum**, **brainstem/medulla oblongata**: Marked, multifocal, subacute to chronic lymphoplasmacytic, neutrophilic and histiocytic leptomeningitis with leptomeningeal vasculitis and endophlebitis, rare intramacrophagic bacteria, perivascular cuffing, gliosis and rare INCIBs.  **Choroid plexus of lateral and fourth ventricles:** Marked, multifocal, acute fibrinosuppurative and necrotizing plexus choroiditis and ventriculitis with necrotizing vasculitis, bacteria and edema, hyalinosis, vascular ectasia (cystic dilatation).  **Prescapular lymph node**: Diffuse lymphoid reactive hyperplasia with sinus histiocytosis  **Mesenteric, Colonic lymph node**: Marked, diffuse, chronic lymphoid depletion with hyalinosis and marked fibrosclerosis; Scattered mineralizations.  **Liver**: Moderate, multifocal, subacute lymphoplasmacytic and histiocytic periportal hepatitis with fibrosis, bile duct hyperplasia and cholestasis; Multifocal acute centrilobular degeneration, necrosis, loss and lobular collapse with hemorrhage, scattered thrombosis, hemosiderosis and cholestasis; MATMHH.  **Spleen**: Diffuse lymphoid reactive hyperplasia with sinus histiocytosis and hemosiderosis; Extramedullary hematopoiesis.  **Large intestine**: Mild, multifocal, chronic lymphoplasmacytic colitis with follicular hyalinosis.  **Small and large intestine**: Moderate, diffuse lymphoplasmacytic enteritis with villar atrophy, epithelial loss and GALT depletion  **Urinary bladder**: Multifocal erosion with leukocytosis and rare INIBs.  **Palate, Esophagus, Heart, Pancreas**: NSLO. | Septic shock by systemic *Staphylococcus aureus* and subacute CeMV infection |
| 26 | Emaciated; Mild parasitosis by *Pennella* sp.; *P. delphini* pyogranulomas in caudoventral subcutis*; M. grimaldii* in muscle, mammary gland and peritoneal serosae; Mediastinal lymphadenomegaly; Hydropericardium; Chronic ulcerative gastritis with anisakids and *P. gastrophilus*; Lack of ingesta; moderate intestinal and bile tree parasitization by Diphyllobothriidae. | **Spinal cord**: Minimal to mild, multifocal lymphocytic polioleukomeningomyelitis and ganglioneuritis with mild gliosis and perivascular cuffing.  **Pulmonary lymph node**: Moderate to marked, diffuse cortical and paracortical lymphoid depletion; Mild, multifocal, chronic eosinophilic lymphadenitis with focal granuloma, lymphangitis and intralesional nematode larvae and scattered MGCS.  **Lung**: Marked, multifocal granulomatous bronchopneumonia with nematodes; Abundant bronchiolar mineralization.  **Spleen**: Mild, diffuse PALS and cord lymphoid hyperplasia with marked hemosiderosis and histiocytosis.  **Cerebrum**: Mild, multifocal lymphocytic encephalitis with neuronal degeneration and necrosis, INCIBs, gliosis, glial nodules and spongiosis.  **Pectoral muscle**: Mild, multifocal, acute segmental myonecrosis.  **Mediastinal, Mesenteric, Parotid lymph nodes**: Mild, multifocal, chronic eosinophilic lymphadenitis and mild, diffuse lymphoid depletion.  **Liver**: Mild, multifocal, chronic lymphocytic periportal hepatitis with mild bile duct hyperplasia.  **Glandular stomach**: Mild, multifocal, chronic erosive-ulcerative gastritis.  **Keratinized stomach**: Mild, multifocal, chronic granulomatous gastritis with *P. gastrophilus*.  **Small and large intestine**: Mild to moderate, multifocal, chronic eosinophilic and lymphoplasmacytic enterocolitis with lymphangiectasia.  **Kidney**: Diffuse congestion.  **Heart, Ovary, Uterus, Adrenal gland**: NSLO | Acute systemic CeMV infection |
| 27 | Emaciated; Three asymmetric circular lesions are observed, with jagged edges affecting skin, subcutaneous muscular bundles and skeleton, with loss of tissues in the left lateral region of the neck, dorsally and laterally the dorsal fin and both sides of the tail peduncle; *P. delphini* plerocercoids in skin and blubber; Mild, multifocal, chronic abdominal polyserositis with adhesions and scattered hemorrhage; Accessory spleen; Pulmonary and mediastinal lymphadenomegaly; Pyogranulomatous and necrotizing pneumonia; Lack of ingesta; Gastric anisakidiasis; presence of a 20 cm-long green net in the keratinized stomach; Brain congestion with multifocal hemorrhage. | **Lung**: Marked, multifocal to coalescing, subacute necrosuppurative to pyogranulomatous and necrotizing bronchopneumonia with vasculitis, thrombosis and myriads of hyphae.  **Cerebrum**: Moderate, multifocal, acute suppurative thromboembolic encephalitis with hyphae and hemorrhage.  **Spleen**: Mild, multifocal hemosiderosis.  **Pectoral muscle**: Mild, multifocal, acute segmental myonecrosis; Scattered myofiber regeneration with mild interstitial fibrosis.  **Mediastinal lymph node:** Mild, diffuse lymphoid depletion.  **Spleen**: Lymphoid reactive hyperplasia.  **Kidney**: Multifocal tubular (medullary) mineralization.  **Ovary**: Multifocal fibrosis.  **Adrenal gland, Pancreas, Thyroid, Spinal cord, Cerebellum**: NSLO. | Pulmonary and thromboembolic cerebral hyphate mycosis; Subacute systemic CeMV infection |

MGCS, Multinucleate giant cells/Syncytia; MATMHH, Multifocal arterial tunica media hypertrophy/hyperplasia; NSLO, No significant lesions observed; GALT, Gut-associated lymphoid tissue; INIBs, intranuclear inclusion bodies; ICIBs, Intracytoplasmic inclusion bodies; NR, not reported; PALS, periarteriolar lymphoid sheath.
