# Supplementary material for: Comparative histopathologic and viral immunohistochemical studies on CeMV infection among Western Mediterranean, Northeast-Central, and Southwestern Atlantic cetaceans
Source: PLoS One. 2019 Mar 20;14(3):e0213363. doi: 10.1371/journal.pone.0213363 (PMC6426187; doi:10.1371/journal.pone.0213363)
Supplement: S5 Table — (DOCX) [file pone.0213363.s006.docx]

**S5 Table.** Main microscopic findings in central nervous system of striped dolphins (*Stenella coeruleoalba*) and bottlenose dolphins (*Tursiops truncatus*) from Canary Islands (Spain) and Italy, and Guiana dolphins (*Sotalia guianensis*) from Brazil.

|  | **Canary Islands** | | | **Italy** | | | **Brazil** | | |
| --- | --- | --- | --- | --- | --- | --- | --- | --- | --- |
|  | A | E | % | A | E | % | A | E | % |
| **Meninges and neuroparenchyma vasculature** |  |  |  |  |  |  |  |  |  |
| Congestion | 45 | 45 | 100 | 13 | 15 | 87 | 22 | 25 | 88 |
| Endothelial hypertrophy | 45 | 45 | 100 | 11 | 15 | 73 | 19 | 25 | 76 |
| Reactive neovascularization | 3 | 45 | 7 | 6 | 15 | 40 | 0 | 25 | 0 |
| Perivascular swelling of astrocytic feet | 41 | 45 | 91 | 12 | 15 | 80 | 20 | 25 | 80 |
| Perivascular hemorrhage | 34 | 45 | 76 | 12 | 15 | 80 | 10 | 25 | 40 |
| Vasculitis/perivasculitis | 30 | 45 | 67 | 7 | 15 | 47 | 0 | 25 | 0 |
| Perivascular cuffing (VRS) | 44 | 45 | 98 | 11 | 15 | 73 | 0 | 25 | 0 |
| Lymphocytes | 44 | 45 | 98 | 9 | 15 | 60 | 0 | 25 | 0 |
| Lymphocytolysis | 15 | 45 | 33 | 0 | 15 | 0 | 0 | 25 | 0 |
| Plasma cells | 27 | 45 | 60 | 8 | 15 | 53 | 0 | 25 | 0 |
| Macrophages | 26 | 45 | 58 | 7 | 15 | 47 | 0 | 25 | 0 |
| Neutrophils | 1 | 45 | 2 | 4 | 15 | 27 | 0 | 25 | 0 |
| Eosinophils | 0 | 45 | 0 | 0 | 15 | 0 | 0 | 25 | 0 |
| Proliferation of adventitial cells (VRS) | 19 | 45 | 42 | 6 | 15 | 40 | 0 | 25 | 0 |
| Thrombosis | 4 | 45 | 9 | 0 | 15 | 0 | 1 | 25 | 4 |
| Fibrosis | 4 | 45 | 9 | 0 | 15 | 0 | 1 | 25 | 4 |
| Necrosis | 8 | 45 | 18 | 0 | 15 | 0 | 0 | 25 | 0 |
| Fibrin | 5 | 45 | 11 | 4 | 15 | 27 | 0 | 25 | 0 |
| Necrotizing vasculitis | 8 | 45 | 18 | 3 | 15 | 20 | 0 | 25 | 0 |
| Leukocytosis | 20 | 45 | 44 | 8 | 15 | 53 | 7 | 25 | 28 |
| **Meninges** |  |  |  |  |  |  |  |  |  |
| Perivascular swelling of astrocytic feet | 39 | 45 | 87 | 4 | 15 | 27 | 2 | 25 | 8 |
| Lymphocytes | 38 | 45 | 84 | 7 | 15 | 47 | 1 | 25 | 4 |
| Plasma cells | 24 | 45 | 53 | 7 | 15 | 47 | 1 | 25 | 4 |
| Macrophages | 23 | 45 | 51 | 7 | 15 | 47 | 0 | 25 | 0 |
| Neutrophils | 5 | 45 | 11 | 4 | 15 | 27 | 0 | 25 | 0 |
| Mott cell | 1 | 45 | 2 | 0 | 15 | 0 | 0 | 25 | 0 |
| Hemorrhage | 12 | 45 | 27 | 1 | 15 | 7 | 2 | 25 | 8 |
| Multinucleate giant cell/Syncytia | 11 | 45 | 24 | 0 | 15 | 0 | 0 | 25 | 0 |
| Edema | 12 | 45 | 27 | 5 | 15 | 33 | 0 | 25 | 0 |
| Fibrosis | 7 | 45 | 16 | 0 | 15 | 0 | 3 | 25 | 12 |
| Necrosis | 6 | 45 | 13 | 4 | 15 | 27 | 0 | 25 | 0 |
| Fibrin | 5 | 45 | 11 | 5 | 15 | 33 | 0 | 25 | 0 |
| Bacteria | 3 | 45 | 7 | 4 | 15 | 27 | 0 | 25 | 0 |
| Calcification | 0 | 45 | 0 | 0 | 15 | 0 | 1 | 25 | 4 |
| **Neuron** |  |  |  |  |  |  |  |  |  |
| Nuclear margination | 34 | 45 | 76 | 9 | 15 | 60 | 2 | 25 | 8 |
| Central chromatolysis | 29 | 45 | 64 | 8 | 15 | 53 | 0 | 25 | 0 |
| Peripheral chromatolysis | 27 | 45 | 60 | 7 | 15 | 47 | 0 | 25 | 0 |
| Acidophilic degeneration | 24 | 45 | 53 | 3 | 15 | 20 | 2 | 25 | 8 |
| Necrosis | 25 | 45 | 56 | 8 | 15 | 53 | 0 | 25 | 0 |
| Liquefactive necrosis | 5 | 45 | 11 | 0 | 15 | 0 | 1 | 25 | 4 |
| Necrosis with neuronophagia | 23 | 45 | 51 | 1 | 15 | 7 | 0 | 25 | 0 |
| Satellitosis | 35 | 45 | 78 | 7 | 15 | 47 | 3 | 25 | 12 |
| Spinal ganglion (satellitosis) |  |  |  |  |  |  |  |  |  |
| Neuronophagic nodules | 28 | 45 | 62 | 2 | 15 | 13 | 0 | 25 | 0 |
| Vacuolar degeneration | 15 | 45 | 33 | 1 | 15 | 7 | 0 | 25 | 0 |
| Pigments (ceroid, lipofuscin) | 2 | 45 | 4 | 1 | 15 | 7 | 1 | 25 | 4 |
| Neuromelanin | 2 | 45 | 4 | 2 | 15 | 13 | 0 | 25 | 0 |
| Viral inclusion bodies | 14 | 45 | 31 | 3 | 15 | 20 | 0 | 25 | 0 |
| Non-viral inclusion bodies (Lafora, etc.) | 2 | 45 | 4 | 0 | 15 | 0 | 1 | 25 | 4 |
| Swollen axons (spheroids) | 34 | 45 | 76 | 3 | 15 | 20 | 1 | 25 | 4 |
| Axon loss | 32 | 45 | 71 | 2 | 15 | 13 | 0 | 25 | 0 |
| Digestion chamber | 22 | 45 | 49 | 2 | 15 | 13 | 0 | 25 | 0 |
| Wallerian degeneration | 18 | 45 | 40 | 2 | 15 | 13 | 0 | 25 | 0 |
| **Astrocytes** |  |  |  |  |  |  |  |  |  |
| Perivascular astrocytic swelling | 40 | 45 | 89 | 11 | 15 | 73 | 20 | 25 | 80 |
| Glia limitans | 26 | 45 | 58 | 5 | 11 | 45 | 2 | 25 | 8 |
| Bergmann’s glia hyperplasia | 6 | 8 | 75 | 1 | 15 | 7 | 0 | 25 | 0 |
| Degeneration, necrosis, loss | 22 | 45 | 49 | 0 | 15 | 0 | 0 | 25 | 0 |
| Gemistocytes | 27 | 45 | 60 | 4 | 15 | 27 | 4 | 25 | 16 |
| Reactive astrogliosis (proliferation) | 34 | 45 | 76 | 9 | 15 | 60 | 7 | 25 | 28 |
| Viral inclusion bodies | 3 | 45 | 7 | 4 | 15 | 27 | 0 | 25 | 0 |
| Alzheimer type II astrocytes | 23 | 45 | 51 | 6 | 15 | 40 | 0 | 25 | 0 |
| Phagocytosis | 1 | 45 | 2 | 0 | 15 | 0 | 0 | 25 | 0 |
| Astrocytosis | 34 | 45 | 76 | 2 | 15 | 13 | 3 | 25 | 12 |
| **Oligodendrocytes/Schwann cells** |  |  |  |  |  |  |  |  |  |
| Hydropic change, swelling | 30 | 45 | 67 | 3 | 15 | 20 | 9 | 25 | 36 |
| Oligodendrogliosis | 31 | 45 | 69 | 10 | 15 | 67 | 2 | 25 | 8 |
| Necrosis | 23 | 45 | 51 | 4 | 15 | 27 | 0 | 25 | 0 |
| Loss | 15 | 45 | 33 | 2 | 15 | 13 | 0 | 25 | 0 |
| Viral inclusion bodies | 3 | 45 | 7 | 1 | 15 | 7 | 0 | 25 | 0 |
| Schwann cell hyperplasia | 5 | 45 | 11 | 1 | 15 | 7 | 0 | 25 | 0 |
| Myelinophagy | 4 | 45 | 9 | 0 | 15 | 0 | 0 | 25 | 0 |
| **Ependymal cells** |  |  |  |  |  |  |  |  |  |
| Atrophy | 3 | 45 | 7 | 0 | 15 | 0 | 0 | 25 | 0 |
| Tearing | 3 | 45 | 7 | 0 | 15 | 0 | 0 | 25 | 0 |
| Discontinuity | 5 | 45 | 11 | 3 | 15 | 20 | 0 | 25 | 0 |
| Ependymitis | 0 | 45 | 0 | 3 | 15 | 20 | 0 | 25 | 0 |
| **Choroid plexus** |  |  |  |  |  |  |  |  |  |
| Tearing | 2 | 45 | 4 | 0 | 15 | 0 | 0 | 25 | 0 |
| Discontinuity | 6 | 45 | 13 | 0 | 15 | 0 | 0 | 25 | 0 |
| Inflammation | 8 | 45 | 18 | 0 | 15 | 0 | 0 | 25 | 0 |
| Fibrosis/hyalinization | 5 | 45 | 11 | 2 | 15 | 13 | 1 | 25 | 4 |
| Congestion, edema, hemorrhage | 2 | 45 | 4 | 0 | 15 | 0 | 0 | 25 | 0 |
| Mineralization | 1 | 45 | 2 | 0 | 15 | 0 | 0 | 25 | 0 |
| **Microglia** |  |  |  |  |  |  |  |  |  |
| Reactive hypertrophy/hyperplasia – Rod cells | 35 | 45 | 78 | 10 | 15 | 67 | 2 | 25 | 8 |
| Microglial nodules | 23 | 45 | 51 | 3 | 15 | 20 | 0 | 25 | 0 |
| Phagocytosis – Gitter cells | 9 | 45 | 20 | 1 | 15 | 7 | 1 | 25 | 4 |
| Perivascular microgliosis with ceroid/lipofuscin | 21 | 45 | 47 | 6 | 15 | 40 | 4 | 25 | 16 |
| Neuronophagia | 5 | 45 | 11 | 2 | 15 | 13 | 0 | 25 | 0 |
| **Interstitium** |  |  |  |  |  |  |  |  |  |
| MGCS | 8 | 45 | 18 | 1 | 15 | 7 | 0 | 25 | 0 |
| Myelinic edema (spongiosis) | 38 | 45 | 84 | 10 | 15 | 67 | 5 | 25 | 20 |
| Hemorrhage | 20 | 45 | 44 | 6 | 15 | 40 | 2 | 25 | 8 |
| Cavitation/rarefaction/necrosis | 6 | 45 | 13 | 2 | 15 | 13 | 1 | 25 | 4 |
| Intravascular/parenchymal hyphae | 0 | 45 | 0 | 1 | 15 | 7 | 0 | 25 | 0 |

A, total of tissue sections affected; E, total of tissue sections evaluated; VRS, Virchow-Robin space; MGCS, Multinucleate giant cell/Syncytia.
