# Supplementary material for: Comparative histopathologic and viral immunohistochemical studies on CeMV infection among Western Mediterranean, Northeast-Central, and Southwestern Atlantic cetaceans
Source: PLoS One. 2019 Mar 20;14(3):e0213363. doi: 10.1371/journal.pone.0213363 (PMC6426187; doi:10.1371/journal.pone.0213363)
Supplement: S6 Table — (DOCX) [file pone.0213363.s007.docx]

**S6 Table.** Main microscopic findings in prescapular, pulmonary, mediastinal and mesenteric lymph nodes of striped dolphins (*Stenella coeruleoalba*) and bottlenose dolphins (*Tursiops truncatus*) from Canary Islands (Spain) and Italy, and Guiana dolphins (*Sotalia guianensis*) from Brazil.

|  | **Prescapular lymph node** | | | | | | | | | **Pulmonary lymph node** | | | | | | | | |
| --- | --- | --- | --- | --- | --- | --- | --- | --- | --- | --- | --- | --- | --- | --- | --- | --- | --- | --- |
|  | A | E | % | A | E | % | A | E | % | A | E | % | A | E | % | A | E | % |
|  | Canary Islands | | | Italy | | | Brazil | | | Canary Islands | | | Italy | | | Brazil | | |
| Reactive hyperplasia | 17 | 42 | 40 | NE | NE | NE | 6 | 54 | 11 | 9 | 42 | 21 | 11 | 24 | 46 | NE | NE | NE |
| Depletion | 15 | 42 | 36 | NE | NE | NE | 33 | 54 | 61 | 20 | 42 | 48 | 10 | 24 | 42 | NE | NE | NE |
| Lymphocytolysis | 6 | 42 | 14 | NE | NE | NE | 14 | 54 | 26 | 4 | 42 | 9 | 3 | 24 | 13 | NE | NE | NE |
| Necrosis | 6 | 42 | 14 | NE | NE | NE | 6 | 54 | 11 | 2 | 42 | 5 | 0 | 24 | 0 | NE | NE | NE |
| Edema | 7 | 42 | 17 | NE | NE | NE | 3 | 54 | 6 | 4 | 42 | 9 | 6 | 24 | 25 | NE | NE | NE |
| Fibrin | 3 | 42 | 7 | NE | NE | NE | 0 | 54 | 0 | 0 | 42 | 0 | 1 | 24 | 4 | NE | NE | NE |
| Erythrocytosis | 6 | 42 | 14 | NE | NE | NE | 4 | 54 | 7 | 6 | 42 | 14 | 2 | 24 | 8 | NE | NE | NE |
| Erythrophagocytosis | 5 | 42 | 12 | NE | NE | NE | 4 | 54 | 7 | 3 | 42 | 7 | 2 | 24 | 8 | NE | NE | NE |
| Leukocytosis | 7 | 42 | 17 | NE | NE | NE | 4 | 54 | 7 | 6 | 42 | 14 | 2 | 24 | 8 | NE | NE | NE |
| Leukophagocytosis | 5 | 42 | 12 | NE | NE | NE | 3 | 54 | 6 | 3 | 42 | 7 | 2 | 24 | 8 | NE | NE | NE |
| Histiocytosis | 7 | 42 | 17 | NE | NE | NE | 9 | 54 | 17 | 6 | 42 | 14 | 4 | 24 | 17 | NE | NE | NE |
| Hemosiderosis | 3 | 42 | 7 | NE | NE | NE | 10 | 54 | 19 | 3 | 42 | 7 | 2 | 24 | 8 | NE | NE | NE |
| Congestion | 13 | 42 | 31 | NE | NE | NE | 1 | 54 | 2 | 20 | 42 | 48 | 6 | 24 | 25 | NE | NE | NE |
| MGCS | 11 | 42 | 26 | NE | NE | NE | 9 | 54 | 17 | 8 | 42 | 19 | 7 | 24 | 29 | NE | NE | NE |
| Lymphocytes | 1 | 42 | 2 | NE | NE | NE | 0 | 54 | 0 | 0 | 42 | 0 | 0 | 24 | 0 | NE | NE | NE |
| Plasma cells | 2 | 42 | 5 | NE | NE | NE | 0 | 54 | 0 | 0 | 42 | 0 | 0 | 24 | 0 | NE | NE | NE |
| Mott cells | 1 | 42 | 2 | NE | NE | NE | 0 | 54 | 0 | 0 | 42 | 0 | 0 | 24 | 0 | NE | NE | NE |
| Macrophages | 9 | 42 | 21 | NE | NE | NE | 0 | 54 | 0 | 5 | 42 | 12 | 1 | 24 | 4 | NE | NE | NE |
| Neutrophils | 11 | 42 | 26 | NE | NE | NE | 0 | 54 | 0 | 7 | 42 | 17 | 0 | 24 | 0 | NE | NE | NE |
| Eosinophils | 8 | 42 | 19 | NE | NE | NE | 12 | 54 | 22 | 13 | 42 | 31 | 5 | 24 | 21 | NE | NE | NE |
| Basophils | 0 | 42 | 0 | NE | NE | NE | 0 | 54 | 0 | 0 | 42 | 0 | 0 | 24 | 0 | NE | NE | NE |
| Mast cells | 0 | 42 | 0 | NE | NE | NE | 0 | 54 | 0 | 0 | 42 | 0 | 0 | 24 | 0 | NE | NE | NE |
| Fibrosis | 4 | 42 | 10 | NE | NE | NE | 11 | 54 | 20 | 4 | 42 | 9 | 8 | 24 | 33 | NE | NE | NE |
| Hyalinosis | 7 | 42 | 17 | NE | NE | NE | 12 | 54 | 22 | 6 | 42 | 14 | 6 | 24 | 25 | NE | NE | NE |
| Amyloid | 0 | 42 | 0 | NE | NE | NE | 0 | 54 | 0 | 0 | 42 | 0 | 0 | 24 | 0 | NE | NE | NE |
| Mineralization | 0 | 42 | 0 | NE | NE | NE | 0 | 54 | 0 | 0 | 42 | 0 | 0 | 24 | 0 | NE | NE | NE |
| Hematopoyesis | 0 | 42 | 0 | NE | NE | NE | 2 | 54 | 4 | 0 | 42 | 0 | 1 | 24 | 4 | NE | NE | NE |
| Capsular hemorrhage | 6 | 42 | 14 | NE | NE | NE | 0 | 54 | 0 | 6 | 42 | 14 | 0 | 24 | 0 | NE | NE | NE |
| Siderocalcinosis | 3 | 42 | 7 | NE | NE | NE | 0 | 54 | 0 | 0 | 42 | 0 | 0 | 24 | 0 | NE | NE | NE |
| INCIBs | 2 | 42 | 5 | NE | NE | NE | 13 | 54 | 24 | 5 | 42 | 12 | 2 | 24 | 8 | NE | NE | NE |
| Vascularization/angiomatosis | 2 | 42 | 5 | NE | NE | NE | 0 | 54 | 0 | 0 | 42 | 0 | 0 | 24 | 0 | NE | NE | NE |
| Fibrinoid vascular necrosis | 2 | 42 | 5 | NE | NE | NE | 0 | 54 | 0 | 0 | 42 | 0 | 0 | 24 | 0 | NE | NE | NE |
| Vascularization/angiomatosis | 2 | 42 | 5 | NE | NE | NE | 11 | 54 | 20 | 3 | 42 | 7 | 2 | 42 | 5 | NE | NE | NE |
| Ceroid (yellow pigment) | 2 | 42 | 5 | NE | NE | NE | 0 | 54 | 0 | 0 | 42 | 0 | 0 | 24 | 0 | NE | NE | NE |
| Melanosis/melanomacrophages | 0 | 42 | 0 | NE | NE | NE | 0 | 54 | 0 | 0 | 42 | 0 | 0 | 24 | 0 | NE | NE | NE |
| Starry sky pattern | 8 | 42 | 19 | NE | NE | NE | 0 | 54 | 0 | 2 | 42 | 5 | 0 | 24 | 0 | NE | NE | NE |

A, total of tissue sections affected; E, total of tissue sections evaluated; VRS, Virchow–Robin space; MGCS, Multinucleate giant cell/syncytia/Warthin-Finkeldey cell.

|  | **Mediastinal lymph node** | | | | | | | | | **Mesenteric lymph node** | | | | | | | | |
| --- | --- | --- | --- | --- | --- | --- | --- | --- | --- | --- | --- | --- | --- | --- | --- | --- | --- | --- |
|  | A | E | % | A | E | % | A | E | % | A | E | % | A | E | % | A | E | % |
|  | Canary Islands | | | Italy | | | Brazil | | | Canary Islands | | | Italy | | | Brazil | | |
| Reactive hyperplasia | 9 | 42 | 21 | 3 | 6 | 50 | 8 | 54 | 15 | 10 | 48 | 21 | 0 | 6 | 0 | 0 | 18 | 0 |
| Depletion | 21 | 42 | 50 | 2 | 6 | 33 | 37 | 54 | 69 | 27 | 48 | 56 | 4 | 6 | 67 | 14 | 18 | 78 |
| Lymphocytolysis | 4 | 42 | 10 | 0 | 6 | 0 | 15 | 54 | 28 | 6 | 48 | 13 | 0 | 6 | 0 | 3 | 18 | 17 |
| Necrosis | 2 | 42 | 5 | 0 | 6 | 0 | 14 | 54 | 26 | 6 | 48 | 13 | 0 | 6 | 0 | 2 | 18 | 11 |
| Edema | 4 | 42 | 10 | 0 | 6 | 0 | 7 | 54 | 13 | 12 | 48 | 25 | 0 | 6 | 0 | 1 | 18 | 6 |
| Fibrin | 0 | 42 | 0 | 0 | 6 | 0 | 0 | 54 | 0 | 1 | 48 | 2 | 0 | 6 | 0 | 0 | 18 | 0 |
| Erythrocytosis | 6 | 42 | 14 | 1 | 6 | 17 | 5 | 54 | 9 | 7 | 48 | 15 | 0 | 6 | 0 | 3 | 18 | 17 |
| Erythrophagocytosis | 3 | 42 | 7 | 1 | 6 | 17 | 3 | 54 | 6 | 6 | 48 | 13 | 0 | 6 | 0 | 2 | 18 | 11 |
| Leukocytosis | 6 | 42 | 14 | 1 | 6 | 17 | 6 | 54 | 11 | 8 | 48 | 17 | 0 | 6 | 0 | 3 | 18 | 17 |
| Leukophagocytosis | 3 | 42 | 7 | 1 | 6 | 17 | 3 | 54 | 6 | 6 | 48 | 13 | 0 | 6 | 0 | 1 | 18 | 6 |
| Histiocytosis | 6 | 42 | 14 | 1 | 6 | 17 | 9 | 54 | 17 | 9 | 48 | 19 | 1 | 6 | 17 | 3 | 18 | 17 |
| Hemosiderosis | 3 | 42 | 7 | 1 | 6 | 17 | 8 | 54 | 15 | 4 | 48 | 8 | 0 | 6 | 0 | 4 | 18 | 22 |
| Congestion | 20 | 42 | 48 | 6 | 6 | 100 | 12 | 54 | 22 | 6 | 48 | 13 | 6 | 6 | 100 | 0 | 18 | 0 |
| Bi-/multinucleate cells | 8 | 42 | 19 | 0 | 6 | 0 | 20 | 54 | 37 | 7 | 48 | 15 | 0 | 6 | 0 | 4 | 18 | 22 |
| MGCS | 0 | 42 | 0 | 0 | 6 | 0 | 19 | 54 | 35 | 6 | 48 | 13 | 0 | 6 | 0 | 1 | 18 | 6 |
| Inflammation | 3 | 42 | 7 | 0 | 6 | 0 | 0 | 54 | 0 | 7 | 48 | 15 | 0 | 6 | 0 | 0 | 18 | 0 |
| Lymphocytes | 0 | 42 | 0 | 0 | 6 | 0 | 0 | 54 | 0 | 2 | 48 | 4 | 0 | 6 | 0 | 0 | 18 | 0 |
| Plasma cells | 0 | 42 | 0 | 0 | 6 | 0 | 0 | 54 | 0 | 0 | 48 | 0 | 0 | 6 | 0 | 0 | 18 | 0 |
| Mott cells | 0 | 42 | 0 | 0 | 6 | 0 | 0 | 54 | 0 | 3 | 48 | 6 | 0 | 6 | 0 | 0 | 18 | 0 |
| Macrophages | 5 | 42 | 12 | 0 | 6 | 0 | 0 | 54 | 0 | 3 | 48 | 6 | 0 | 6 | 0 | 0 | 18 | 0 |
| Neutrophils | 7 | 42 | 17 | 0 | 6 | 0 | 2 | 54 | 4 | 8 | 48 | 17 | 0 | 6 | 0 | 0 | 18 | 0 |
| Eosinophils | 13 | 42 | 31 | 1 | 6 | 17 | 17 | 54 | 31 | 27 | 48 | 56 | 1 | 6 | 17 | 5 | 18 | 28 |
| Basophils | 0 | 42 | 0 | 0 | 6 | 0 | 0 | 54 | 0 | 0 | 48 | 0 | 0 | 6 | 0 | 0 | 18 | 0 |
| Mast cells | 0 | 42 | 0 | 0 | 6 | 0 | 0 | 54 | 0 | 0 | 48 | 0 | 0 | 6 | 0 | 0 | 18 | 0 |
| Fibrosis | 4 | 42 | 10 | 0 | 6 | 0 | 22 | 54 | 41 | 11 | 48 | 23 | 0 | 6 | 0 | 5 | 18 | 28 |
| Hyalinosis | 7 | 42 | 17 | 0 | 6 | 0 | 15 | 54 | 28 | 8 | 48 | 17 | 0 | 6 | 0 | 5 | 18 | 28 |
| Amyloid | 0 | 42 | 0 | 0 | 6 | 0 | 0 | 54 | 0 | 1 | 48 | 2 | 0 | 6 | 0 | 0 | 18 | 0 |
| Mineralization | 0 | 42 | 0 | 0 | 6 | 0 | 0 | 54 | 0 | 0 | 48 | 0 | 0 | 6 | 0 | 0 | 18 | 0 |
| Hematopoiesis | 5 | 42 | 12 | 6 | 6 | 100 | 0 | 54 | 0 | 3 | 48 | 6 | 6 | 6 | 100 | 0 | 18 | 0 |
| Capsular hemorrhage | 2 | 42 | 5 | 0 | 6 | 0 | 0 | 54 | 0 | 0 | 48 | 0 | 0 | 6 | 0 | 0 | 18 | 0 |
| Siderocalcinosis | 3 | 42 | 7 | 0 | 6 | 0 | 0 | 54 | 0 | 0 | 48 | 0 | 0 | 6 | 0 | 0 | 18 | 0 |
| INCIBs | 5 | 42 | 12 | 0 | 6 | 0 | 3 | 54 | 6 | 1 | 48 | 2 | 0 | 6 | 0 | 0 | 18 | 0 |
| Vascularization/angiomatosis | 0 | 42 | 0 | 0 | 6 | 0 | 0 | 54 | 0 | 10 | 48 | 21 | 0 | 6 | 0 | 0 | 18 | 0 |
| Capillary mineralization | 0 | 42 | 0 | 0 | 6 | 0 | 0 | 54 | 0 | 3 | 48 | 6 | 0 | 6 | 0 | 0 | 18 | 0 |
| Tunica media HH | 3 | 42 | 7 | 2 | 6 | 3 | 7 | 54 | 13 | 14 | 48 | 29 | 0 | 6 | 0 | 0 | 18 | 0 |
| Arteriosclerosis | 3 | 42 | 7 | 2 | 6 | 3 | 7 | 54 | 13 | 7 | 48 | 15 | 6 | 6 | 100 | 0 | 18 | 0 |
| Fibrinoid vascular necrosis | 2 | 42 | 5 | 0 | 6 | 0 | 0 | 54 | 0 | 2 | 48 | 4 | 0 | 6 | 0 | 0 | 18 | 0 |

A, total of tissue sections affected; E, total of tissue sections evaluated; VRS, Virchow–Robin space; MGCS, Multinucleate giant cell/Syncytia.
