# Supplementary material for: Comparative histopathologic and viral immunohistochemical studies on CeMV infection among Western Mediterranean, Northeast-Central, and Southwestern Atlantic cetaceans
Source: PLoS One. 2019 Mar 20;14(3):e0213363. doi: 10.1371/journal.pone.0213363 (PMC6426187; doi:10.1371/journal.pone.0213363)
Supplement: S7 Table — (DOCX) [file pone.0213363.s008.docx]

**S7 Table.** Main microscopic findings in spleen of striped dolphins (*Stenella coeruleoalba*) and bottlenose dolphins (*Tursiops truncatus*) from Canary Islands (Spain) and Italy, and Guiana dolphins (*Sotalia guianensis*) from Brazil.

|  | **Canary Islands** | | | | **Italy** | | | | **Brazil** | | |
| --- | --- | --- | --- | --- | --- | --- | --- | --- | --- | --- | --- |
|  | A | E | % | A | | E | % | A | | E | % |
| Congestion | 24 | 42 | 57 | 31 | | 42 | 74 | 7 | | 48 | 15 |
| Reactive hyperplasia | 14 | 42 | 33 | 12 | | 42 | 29 | 5 | | 48 | 10 |
| Depletion | 15 | 42 | 36 | 17 | | 42 | 40 | 26 | | 48 | 54 |
| Lymphocytolysis | 3 | 42 | 7 | 0 | | 42 | 0 | 0 | | 48 | 0 |
| Necrosis | 1 | 42 | 2 | 0 | | 42 | 0 | 0 | | 48 | 0 |
| Edema | 1 | 42 | 2 | 0 | | 42 | 0 | 0 | | 48 | 0 |
| Fibrin | 1 | 42 | 2 | 0 | | 42 | 0 | 1 | | 48 | 2 |
| Erythrocytosis | 0 | 42 | 0 | 0 | | 42 | 0 | 0 | | 48 | 0 |
| Erythrophagocytosis | 3 | 42 | 7 | 0 | | 42 | 0 | 1 | | 48 | 2 |
| Leukocytosis | 3 | 42 | 7 | 0 | | 42 | 0 | 0 | | 48 | 0 |
| Leukophagocytosis | 0 | 42 | 0 | 0 | | 42 | 0 | 0 | | 48 | 0 |
| Histiocytosis | 5 | 42 | 12 | 7 | | 42 | 17 | 8 | | 48 | 17 |
| Hemosiderosis | 0 | 42 | 0 | 7 | | 42 | 17 | 11 | | 48 | 23 |
| Hemorrhage | 1 | 42 | 2 | 6 | | 42 | 14 | 9 | | 48 | 19 |
| MGCS | 0 | 42 | 0 | 0 | | 42 | 0 | 5 | | 48 | 10 |
| Macrophagic inflammation | 1 | 42 | 2 | 0 | | 42 | 0 | 0 | | 48 | 0 |
| Neutrophilic inflammation | 0 | 42 | 0 | 0 | | 42 | 0 | 1 | | 48 | 2 |
| Eosinophilic inflammation | 2 | 42 | 5 | 2 | | 42 | 5 | 12 | | 48 | 25 |
| Fibrosis | 2 | 42 | 5 | 0 | | 42 | 0 | 0 | | 48 | 0 |
| Hyalinosis | 13 | 42 | 31 | 11 | | 42 | 26 | 5 | | 48 | 10 |
| Amyloid | 2 | 42 | 5 | 0 | | 42 | 0 | 0 | | 48 | 0 |
| Mineralization | 1 | 42 | 2 | 0 | | 42 | 0 | 0 | | 48 | 0 |
| Hematopoiesis | 8 | 42 | 19 | 6 | | 42 | 14 | 8 | | 48 | 17 |
| Capsular hemorrhage | 18 | 42 | 43 | 0 | | 42 | 0 | 0 | | 48 | 0 |
| Siderocalcinosis | 4 | 42 | 9 | 12 | | 42 | 29 | 0 | | 48 | 0 |
| INCIBs | 3 | 42 | 7 | 0 | | 42 | 0 | 1 | | 48 | 2 |
| Thrombosis | 0 | 42 | 0 | 6 | | 42 | 14 | 0 | | 48 | 0 |

A, total of tissue sections affected; E, total of tissue sections evaluated; VRS, Virchow-Robin space; MGCS, Multinucleate giant cell/Syncytia.
