# Supplementary material for: Comparative histopathologic and viral immunohistochemical studies on CeMV infection among Western Mediterranean, Northeast-Central, and Southwestern Atlantic cetaceans
Source: PLoS One. 2019 Mar 20;14(3):e0213363. doi: 10.1371/journal.pone.0213363 (PMC6426187; doi:10.1371/journal.pone.0213363)
Supplement: S8 Table — (DOCX) [file pone.0213363.s009.docx]

**S8 Table.** Main microscopic findings in the respiratory system of striped dolphins (*Stenella coeruleoalba*) and Atlantic bottlenose dolphins (*Tursiops truncatus*) from the Canary Islands (Spain) and Italy, and Guiana dolphins (*Sotalia guianensis*) from Brazil.

|  | **Canary Islands** | | | **Italy** | | | **Brazil** | | |
| --- | --- | --- | --- | --- | --- | --- | --- | --- | --- |
|  | A | E | % | A | E | % | A | E | % |
| **Vasculature** |  |  |  |  |  |  |  |  |  |
| Congestion | 40 | 40 | 100 | 34 | 41 | 83 | 8 | 59 | 14 |
| Angiomatosis | 12 | 16 | 75 | 14 | 41 | 34 | 17 | 59 | 29 |
| Endothelial hypertrophy | 29 | 40 | 72 | 14 | 41 | 34 | 34 | 59 | 58 |
| Tunica media HH | 13 | 40 | 32 | 7 | 41 | 17 | 17 | 59 | 29 |
| Vasculitis/Perivasculitis | 2 | 40 | 5 | 9 | 41 | 22 | 3 | 59 | 5 |
| Thrombosis | 4 | 40 | 10 | 5 | 41 | 12 | 3 | 59 | 3 |
| Perivascular edema | 9 | 40 | 22 | 0 | 41 | 0 | 0 | 59 | 0 |
| Perivascular hemorrhage | 11 | 40 | 27 | 0 | 41 | 0 | 0 | 59 | 0 |
| Arteriosclerosis | 5 | 40 | 12 | 5 | 41 | 12 | 2 | 59 | 3 |
| Endarteritis | 0 | 40 | 0 | 0 | 41 | 0 | 4 | 59 | 7 |
| **Mucosa epithelium** |  |  |  |  |  |  |  |  |  |
| Degeneration | 20 | 40 | 50 | 14 | 41 | 34 | 31 | 59 | 53 |
| Necrosis | 9 | 40 | 22 | 9 | 41 | 22 | 29 | 59 | 49 |
| Sloughing/loss | 17 | 40 | 42 | 11 | 41 | 27 | 29 | 59 | 49 |
| Type II pneumocyte hyperplasia | 8 | 40 | 20 | 4 | 41 | 9 | 11 | 59 | 19 |
| MGCS | 10 | 40 | 25 | 2 | 41 | 5 | 3 | 59 | 5 |
| Metaplasia | 8 | 40 | 20 | 3 | 41 | 7 | 2 | 59 | 3 |
| INCIBs | 10 | 40 | 25 | 2 | 41 | 5 | 15 | 59 | 17 |
| **Submucosa** |  |  |  |  |  |  |  |  |  |
| Mineralization | 24 | 40 | 60 | 21 | 41 | 51 | 29 | 59 | 49 |
| Edema | 16 | 40 | 40 | 11 | 41 | 27 | 8 | 59 | 14 |
| Fibrin | 2 | 40 | 5 | 4 | 41 | 10 | 3 | 59 | 5 |
| Hemorrhage | 3 | 40 | 7 | 8 | 41 | 20 | 6 | 59 | 10 |
| Neutrophils | 3 | 40 | 7 | 6 | 41 | 15 | 6 | 59 | 10 |
| Eosinophils | 4 | 40 | 10 | 3 | 41 | 7 | 5 | 59 | 8 |
| Macrophages | 14 | 40 | 35 | 14 | 41 | 34 | 19 | 59 | 32 |
| Lymphocytes | 22 | 40 | 55 | 18 | 41 | 44 | 22 | 59 | 37 |
| Plasma cells | 17 | 40 | 42 | 13 | 41 | 32 | 5 | 59 | 8 |
| Hemorrhage | 0 | 40 | 0 | 3 | 41 | 7 | 3 | 59 | 5 |
| Fibrosis | 23 | 40 | 57 | 17 | 41 | 41 | 27 | 59 | 46 |
| Chondronecrosis/-lysis | 10 | 40 | 25 | 3 | 41 | 7 | 4 | 59 | 6 |
| Fibromuscular hyperplasia | 5 | 40 | 12 | 0 | 41 | 0 | 0 | 59 | 0 |
| **Lumen** |  |  |  |  |  |  |  |  |  |
| Edema | 21 | 40 | 52 | 22 | 41 | 54 | 14 | 59 | 24 |
| Fibrin | 5 | 40 | 12 | 8 | 41 | 20 | 10 | 59 | 17 |
| Hemorrhage | 10 | 40 | 25 | 6 | 41 | 15 | 14 | 59 | 24 |
| Neutrophils | 12 | 40 | 30 | 8 | 41 | 20 | 12 | 59 | 20 |
| Eosinophils | 7 | 40 | 17 | 6 | 41 | 15 | 9 | 59 | 15 |
| Macrophages | 13 | 40 | 32 | 15 | 41 | 37 | 13 | 59 | 22 |
| Lymphocytes | 4 | 40 | 10 | 2 | 41 | 5 | 0 | 59 | 0 |
| Plasma cells | 0 | 40 | 0 | 0 | 41 | 0 | 0 | 59 | 0 |
| Keratin squames/meconium | 0 | 40 | 0 | 0 | 41 | 0 | 1 | 59 | 2 |
| MGCS | 8 | 40 | 20 | 2 | 41 | 5 | 6 | 59 | 10 |
| Necrotic cell debris | 11 | 40 | 27 | 3 | 41 | 7 | 12 | 59 | 20 |
| Bacteria | 4 | 40 | 10 | 6 | 41 | 15 | 2 | 59 | 3 |
| Nematodes | 11 | 40 | 27 | 5 | 41 | 12 | 7 | 59 | 12 |
| Fungi | 0 | 40 | 0 | 3 | 41 | 7 | 4 | 59 | 7 |
| Emphysema | 4 | 40 | 10 | 5 | 41 | 12 | 1 | 59 | 2 |
| Abscess | 0 | 40 | 0 | 4 | 41 | 10 | 0 | 59 | 0 |
| Bronchiectasis | 0 | 40 | 0 | 2 | 41 | 5 | 4 | 59 | 7 |
| Ceroid-like lipid globules | 0 | 40 | 0 | 2 | 41 | 5 | 0 | 59 | 0 |
| Necrotic cell debris | 12 | 40 | 30 | 5 | 41 | 12 | 12 | 59 | 20 |
| Aspirated particles | 0 | 40 | 0 | 1 | 41 | 2 | 3 | 59 | 5 |
| Mucus | 0 | 40 | 0 | 0 | 41 | 0 | 2 | 59 | 3 |
| Cholesterol clefts | 0 | 40 | 0 | 0 | 41 | 0 | 1 | 59 | 2 |
| Hemosiderophages | 5 | 40 | 12 | 6 | 41 | 15 | 2 | 59 | 3 |
| Bronchoconstriction | 14 | 40 | 35 | 0 | 41 | 0 | 0 | 59 | 0 |
| Atelectasia | 12 | 40 | 30 | 0 | 41 | 0 | 0 | 59 | 0 |
| Large calcifications | 0 | 40 | 0 | 0 | 41 | 0 | 4 | 59 | 7 |
| **Interstitium** |  |  |  |  |  |  |  |  |  |
| Edema | 3 | 40 | 7 | 4 | 41 | 10 | 5 | 59 | 8 |
| Fibrin | 0 | 40 | 0 | 2 | 41 | 5 | 3 | 59 | 5 |
| Hemorrhage | 0 | 40 | 0 | 2 | 41 | 5 | 3 | 59 | 5 |
| Neutrophils | 0 | 40 | 0 | 2 | 41 | 5 | 4 | 59 | 7 |
| Eosinophils | 0 | 40 | 0 | 1 | 41 | 2 | 4 | 59 | 7 |
| Macrophages | 5 | 40 | 12 | 7 | 41 | 17 | 11 | 59 | 19 |
| Lymphocytes | 9 | 40 | 22 | 8 | 41 | 20 | 11 | 59 | 19 |
| Plasma cells | 6 | 40 | 15 | 7 | 41 | 17 | 5 | 59 | 8 |
| Necrotic cell debris | 3 | 40 | 7 | 2 | 41 | 5 | 4 | 59 | 7 |
| Hemosiderosis | 3 | 40 | 7 | 0 | 41 | 0 | 3 | 59 | 5 |
| Fibrosis | 7 | 40 | 17 | 10 | 41 | 24 | 12 | 59 | 20 |
| Severe remodeling | 4 | 40 | 10 | 1 | 41 | 2 | 8 | 59 | 14 |
| Sclerotic granulomas | 7 | 40 | 17 | 1 | 41 | 0 | 3 | 59 | 5 |
| Large calcification | 0 | 40 | 0 | 0 | 41 | 0 | 1 | 59 | 2 |
| **Pleura** |  |  |  |  |  |  |  |  |  |
| Macrophages | 5 | 40 | 12 | 5 | 41 | 12 | 0 | 59 | 0 |
| Lymphocytes | 5 | 40 | 12 | 6 | 41 | 15 | 0 | 59 | 0 |
| Plasma cells | 2 | 40 | 5 | 6 | 41 | 15 | 0 | 59 | 0 |
| Fibrosis | 6 | 40 | 15 | 3 | 41 | 7 | 4 | 59 | 7 |

A, total of tissue sections affected; E, total of tissue sections evaluated; INCIBs, intranuclear/-cytoplasmic inclusion bodies; MGCS, Multinucleate giant cell/Syncytia.
